# Natural Variation in a Dendritic Scaffold Protein Remodels Experience-Dependent Plasticity by Altering Neuropeptide Expression

**DOI:** 10.1016/j.neuron.2019.10.001

**Published:** 2020-01-08

**Authors:** Isabel Beets, Gaotian Zhang, Lorenz A. Fenk, Changchun Chen, Geoffrey M. Nelson, Marie-Anne Félix, Mario de Bono

**Affiliations:** 1 Cell Biology Division, MRC Laboratory of Molecular Biology, Cambridge CB2 0QH, UK; 2 Institut de Biologie de l’École Normale Supérieure, CNRS, Inserm, PSL Research University, Paris 75005, France

**Keywords:** experience-dependent plasticity, natural variation, neuropeptide, genetic accommodation, *Caenorhabditis elegans,* carbon dioxide sensing, oxygen sensing

## Abstract

The extent to which behavior is shaped by experience varies between individuals. Genetic differences contribute to this variation, but the neural mechanisms are not understood. Here, we dissect natural variation in the behavioral flexibility of two *Caenorhabditis elegans* wild strains. In one strain, a memory of exposure to 21% O_2_ suppresses CO_2_-evoked locomotory arousal; in the other, CO_2_ evokes arousal regardless of previous O_2_ experience. We map that variation to a polymorphic dendritic scaffold protein, ARCP-1, expressed in sensory neurons. ARCP-1 binds the Ca^2+^-dependent phosphodiesterase PDE-1 and co-localizes PDE-1 with molecular sensors for CO_2_ at dendritic ends. Reducing ARCP-1 or PDE-1 activity promotes CO_2_ escape by altering neuropeptide expression in the BAG CO_2_ sensors. Variation in ARCP-1 alters behavioral plasticity in multiple paradigms. Our findings are reminiscent of genetic accommodation, an evolutionary process by which phenotypic flexibility in response to environmental variation is reset by genetic change.

## Highlights

• Behavioral flexibility varies across *Caenorhabditis* and *C. elegans* wild isolates
• A natural polymorphism in ARCP-1 underpins inter-individual variation in plasticity
• ARCP-1 is a dendritic scaffold protein localizing cGMP signaling machinery to cilia
• Disrupting ARCP-1 alters behavioral plasticity by changing neuropeptide expression

## Teaser

Individuals can vary in their capacity to adapt their behavior to changes in the environment. Beets et al. identify a natural genetic polymorphism that modifies behavioral flexibility by altering the neuromodulatory output of primary sensory neurons.

## Introduction

Animals reconfigure their behavior and physiology in response to experience, and many studies highlight mechanisms underlying such plasticity (Bargmann, 2012, Owen and Brenner, 2012). While plasticity is presumed crucial for evolutionary success, it has costs and often varies across species and between individuals (Coppens et al., 2010, Dewitt et al., 1998, Mery, 2013, Niemelä et al., 2013). Variation in behavioral flexibility is thought to underlie inter-individual differences in cognitive ability and capacity to cope with environmental challenges (Coppens et al., 2010, Niemelä et al., 2013). The genetic and cellular basis of inter-individual variation in experience-dependent plasticity is, however, poorly understood.

Genetic accommodation and assimilation are concepts used to describe variation in plasticity on an evolutionary timescale. Waddington and Schmalhausen suggested genetic assimilation occurs when a phenotype initially responsive to the environment becomes fixed in a specific state (Renn and Schumer, 2013, Schmalhausen, 1949, Waddington, 1942, Waddington, 1953). This loss of plasticity may reflect genetic drift or selection against the costs of expressing adaptive behaviors (Niemelä et al., 2013). Studies of genetic assimilation led to the broader concept of genetic accommodation, referring to evolutionary genetic variation leading to any change in the environmental regulation of a phenotype (Crispo, 2007, West-Eberhard, 2005). Many studies in insects, fish, rodents, and primates highlight inter-individual variation in behavioral plasticity; in some cases this has been shown to be heritable (Dingemanse and Wolf, 2013, Izquierdo et al., 2007, Mery et al., 2007), but the mechanisms responsible for these differences remain enigmatic.

Many animals use gradients of respiratory gases to help locate prey, mates, or predators and have evolved sophisticated behavioral responses to environmental changes in oxygen (O_2_) and carbon dioxide (CO_2_) levels (Carrillo and Hallem, 2015, Cummins et al., 2014, Guerenstein and Hildebrand, 2008, Prabhakar and Semenza, 2015). Where studied, behavioral responses to CO_2_ have been shown to depend on environmental context, past experience, and life stage (Carrillo et al., 2013, Fenk and de Bono, 2017, Guillermin et al., 2017, Hallem and Sternberg, 2008, Sachse et al., 2007, Vulesevic et al., 2006). This flexibility makes CO_2_-sensing an attractive paradigm to study natural variation in behavioral plasticity.

CO_2_ responses in *Caenorhabditis elegans* are sculpted by previous O_2_ experience (Carrillo et al., 2013, Fenk and de Bono, 2017, Kodama-Namba et al., 2013). Acclimation to surface O_2_ levels (i.e., 21%) generates a memory that suppresses aversion of high CO_2_. The O_2_ memory is written over hours by O_2_ sensors, called URX, AQR, and PQR, whose activity is tonically stimulated by 21% O_2_ (Busch et al., 2012, Fenk and de Bono, 2017). 21% O_2_ is itself aversive to *C. elegans*, most likely because it signals surface exposure (Gray et al., 2004, Persson et al., 2009). By suppressing CO_2_ aversiveness, *C. elegans* acclimated to 21% O_2_ may increase their chance of escaping the surface into buried environments with elevated CO_2_ (Fenk and de Bono, 2017).

Here, we show that the impact of O_2_ experience on CO_2_ aversion varies across *Caenorhabditis* species and between wild *C. elegans* isolates. By characterizing differences between *C. elegans* isolates, we identify a polymorphism in a dendritic ankyrin-repeat scaffold protein, ARCP-1, that alters plasticity in one strain. ARCP-1 biochemically interacts with the conserved cyclic nucleotide phosphodiesterase PDE-1 and localizes it with molecular sensors for CO_2_ to the dendritic ends of BAG sensory neurons. Disrupting ARCP-1 resets CO_2_ sensitivity and experience-dependent plasticity of CO_2_ escape, in part by altering neuropeptide expression and conferring strong aversion to CO_2_.

## Results

### Natural Variation in Experience-Dependent Plasticity in *Caenorhabditis*

In *C. elegans*, a memory of recent O_2_ levels reprograms aversive responses to CO_2_ (Fenk and de Bono, 2017). We hypothesized this experience-dependent plasticity is evolutionarily variable. To investigate this, we compared the CO_2_ responses of different *Caenorhabditis* species grown at 21% or 7% O_2_ (Figure S1A). Animals were transferred to a thin bacterial lawn in a microfluidic chamber kept at 7% O_2_, stimulated with 3% CO_2_, and their behavioral responses quantified. We used a background level of 7% O_2_ in all assays because *C. elegans* dwell locally at this O_2_ concentration, making locomotory arousal by CO_2_ prominent. By contrast, 21% O_2_ evokes sustained rapid movement, making CO_2_ responses above this high baseline proportionally small. As a representative *C. elegans* strain, we used LSJ1, a wild-type (N2-like) laboratory strain bearing natural alleles of the neuropeptide receptor *npr-1*(*215F*) and the neuroglobin *glb-5*(*Haw*). We did not use the standard N2 strain, because it has acquired mutations in *npr-1* and *glb-5* that confer gas-sensing defects (McGrath et al., 2009, Persson et al., 2009). As expected, *C. elegans* was aroused more strongly by CO_2_ when acclimated to 7% O_2_ (Figure S1B). By contrast, O_2_ experience did not alter the absolute speed of representative strains of *C. latens* and *C. angaria* at 3% CO_2_ (Figure S1B). Because *C. angaria* was not aroused by 3% CO_2_, we tested its response to 5% and 10% CO_2_. These levels evoked locomotory arousal that, as in *C. elegans*, was stronger in animals acclimated to 7% O_2_ (Figure S1C). Thus, *C. angaria* is less sensitive to CO_2_ than *C. elegans*, but its arousal by CO_2_ remains dependent on O_2_ experience. By contrast, CO_2_ responses of *C. latens* were unaffected by previous O_2_ experience at any concentration tested (Figure S1D). Unexpectedly, acclimation to 7% O_2_ suppressed rather than enhanced the locomotory response of *C. nigoni* to CO_2_ (Figure S1B). Thus, the effect of O_2_ memory on CO_2_-evoked behavioral responses is evolutionarily variable.

### Effect of O_2_ Memory on CO_2_ Responses Varies between *C. elegans* Wild Isolates

**Figure 1:**
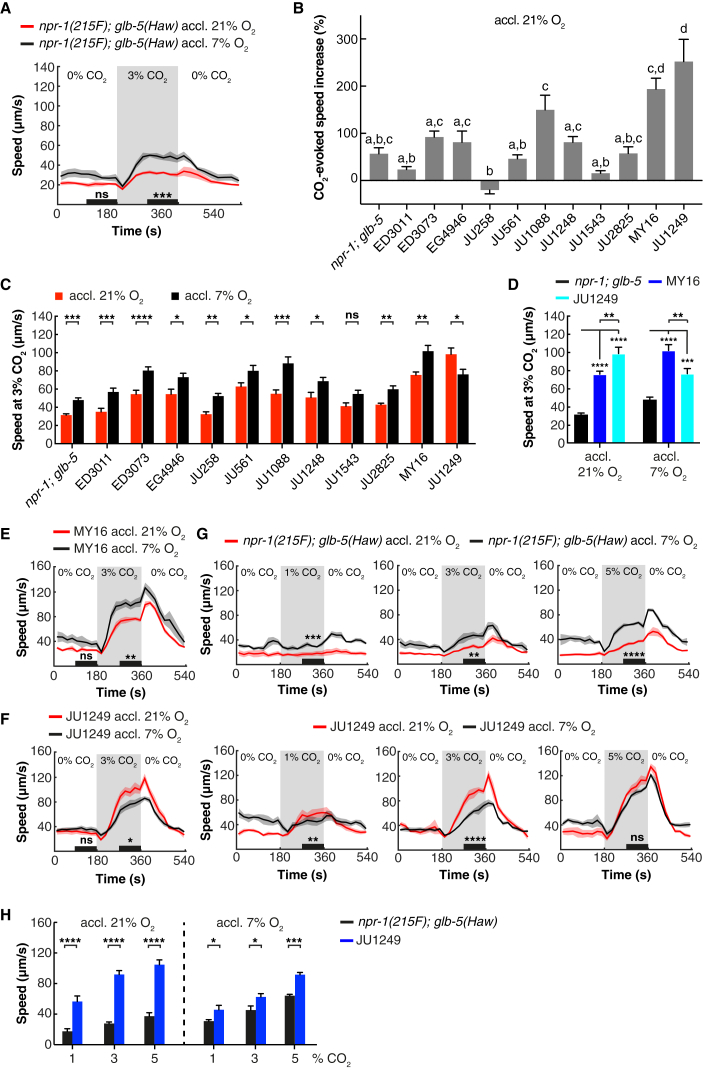
Natural Variation in the Regulation of CO_2_ Escape by Previous O_2_ Experience (A) A *C. elegans* reference strain is more strongly aroused by CO_2_ when acclimated to 7% rather than 21% O_2_. Two-way ANOVA with Šidák test; n = 6 assays. In this, and all subsequent figures, the background O_2_ level in the assay is 7%. (B) Natural variation in the CO_2_ response of *C. elegans* wild isolates acclimated to 21% O_2_. Bars represent average increase in speed ± SEM when CO_2_ rises from 0% to 3%. The CO_2_-evoked speed increase is significantly different (p < 0.05) between isolates labeled with different letters (a–d). One-way ANOVA with Tukey test; n = 6 assays. (C) The effect of O_2_ memory on CO_2_ responses in wild *C. elegans* isolates. Bars show mean ± SEM for time intervals indicated in (A) and Figure S2A. Two-way ANOVA with Šidák test; n = 6 assays. (D) JU1249 and MY16 are more strongly aroused by CO_2_, regardless of previous O_2_ experience. Bars plot mean ± SEM. Two-way ANOVA with Tukey test; n = 6 assays. (E and F) CO_2_ responses of MY16 (E) and JU1249 (F) animals acclimated to 21% or 7% O_2_. Two-way ANOVA with Šidák test; n = 6 assays. (G) Acclimation to 21% O_2_ in JU1249, unlike the reference strain LSJ1, enhances rather than suppresses locomotory arousal at different CO_2_ concentrations. n = 30–61 animals for *npr-1; glb-5*, n = 59–66 animals for JU1249. Mann-Whitney U test. (H) CO_2_ arousal is increased more strongly in JU1249 animals acclimated to 21% rather than 7% O_2_. Bars plot mean ± SEM for time intervals indicated in (G). Two-way ANOVA with Šidák test. n = 4 assays. For (A), (E), and (F), solid lines plot mean and shaded areas show SEM. Black bars indicate time intervals used for statistical comparisons. For (A)–(H), 20–30 animals were assayed in at least 4 trials for each condition. ^∗^p < 0.05; ^∗∗^p < 0.01; ^∗∗∗^p < 0.001; ^∗∗∗∗^p < 0.0001; ns, not significant. See also Figures S1 and S2.

Our findings prompted us to seek intra-species variation in how O_2_ experience influences CO_2_ responses, by studying a genetically diverse collection of wild *C. elegans* isolates (Figure S2A). Most strains responded like the reference strain (Figures 1A–1C and S2A). However, two isolates, the French JU1249 and German MY16 strains, responded more strongly than other isolates to a rise in CO_2_ regardless of O_2_ experience (Figures 1B and 1D). For MY16 CO_2_ aversion was stronger when animals were acclimated to 7% O_2_, recapitulating the cross-modulation of CO_2_ responses observed in most strains (Figures 1C and 1E). By contrast, JU1249 animals acclimated to 21% O_2_ further enhanced rather than suppressed CO_2_ escape (Figures 1C and 1F). To probe further if the O_2_-dependent plasticity of CO_2_ escape had changed in JU1249, we quantified escape responses at different CO_2_ concentrations. *npr-1; glb-5* control animals always responded more strongly to CO_2_ when acclimated to 7% O_2_, but this was not the case for JU1249 at any CO_2_ concentration tested (Figure 1G). CO-evoked arousal was stronger in JU1249 animals acclimated to 21% O_2_ than in those acclimated to 7% O_2_ (Figure 1H), suggesting that JU1249 fails to suppress CO_2_ escape at 21% O_2_.

The increased locomotory arousal of JU1249 and MY16 in response to CO_2_ could reflect reduced inhibitory input from the neural circuit signaling 21% O_2_. To probe this, we asked if these isolates show altered behavioral responses to 21% O_2_. All isolates we tested responded similarly when we switched O_2_ from 7% to 21% (Figure S2B), suggesting they retained a functional O_2_-sensing circuit.

In our assays, we exposed animals acclimated to 21% O_2_ to a downshift to 7% O_2_ 3 min before the CO_2_ stimulus. To ask if this drop in O_2_, rather than O_2_ experience, altered the CO_2_ response in JU1249, we extended the time animals spent at 7% O_2_ prior to receiving the CO_2_ stimulus to 24 min. JU1249 was still more strongly aroused by CO_2_ when acclimated to 21% rather than 7% O_2_; as expected, O_2_ experience had the opposite effect on plasticity in *npr-1; glb-5* controls (Figure S2C). We also compared the behavioral responses of JU1249 and *npr-1; glb-5* animals to a 21% to 7% O_2_ stimulus and found no significant differences (Figure S2D). Thus, the ability of an O_2_ memory to modify CO_2_ escape appears to be altered in JU1249, recapitulating the phenotype observed in *C. nigoni*.

### Natural Variation in the Ankyrin Repeat Protein ARCP-1 Alters Plasticity of CO_2_ Responses

**Figure 2:**
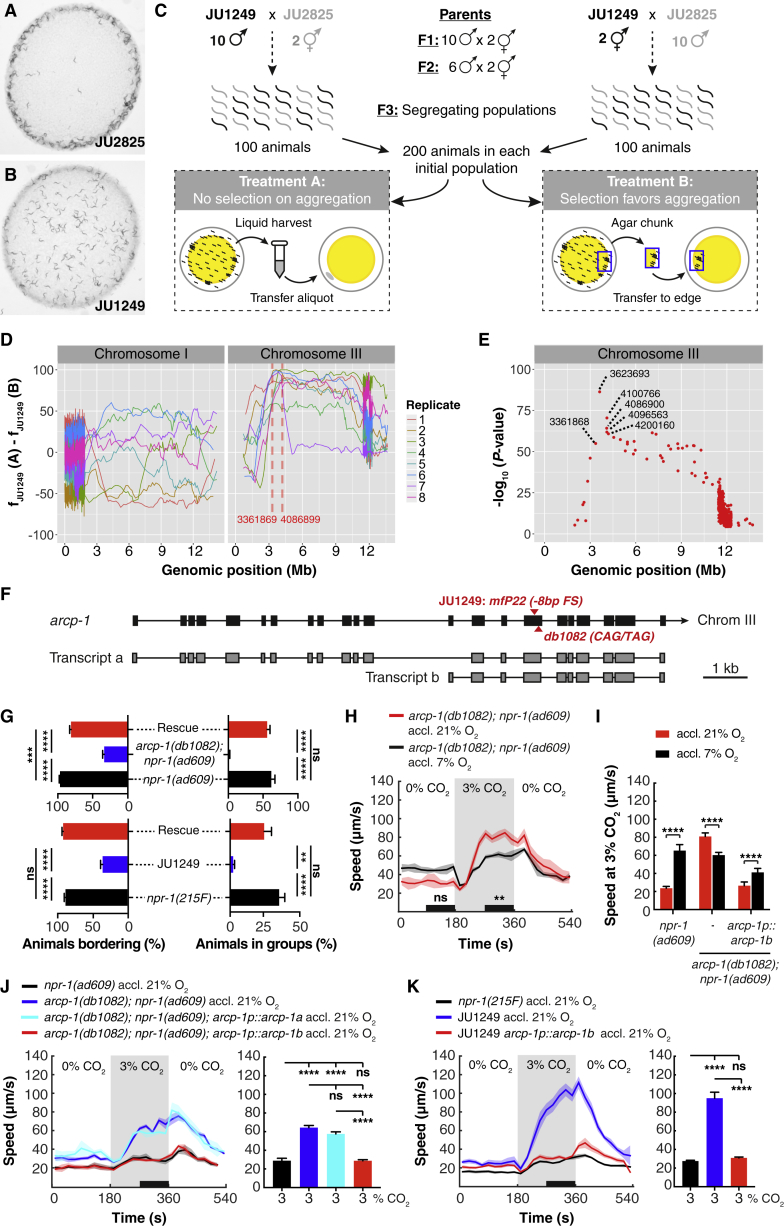
Natural Variation in ARCP-1 Alters CO_2_ Responses (A and B) Individuals of JU2825, like most *C. elegans* wild isolates, aggregate at the border of an *E. coli* lawn (A). By contrast, JU1249 animals disperse across the lawn (B). (C) Selection-based QTL mapping approach to establish the genetic basis of solitary behavior in JU1249. (D) Line plots showing differences in JU1249 allele frequencies between treatment A and B for each replicate pair, using a sliding window 5 single-nucleotide polymorphisms (SNPs) wide and a step size of one SNP. Replicates are indicated by different colors. Chromosome I shows little consistent deviations from equal frequencies in the two treatments, whereas chromosome III shows a strong enrichment at the 3–4 Mb interval. (E) Read-count frequency differences between treatment A and B analyzed for consistency across eight replicates using the Cochran-Mantel-Haenszel test. Only chromosome III is shown. p values are shown as –log_10_ (p value) adjusted by the Bonferroni correction. (F) Gene structure of *arcp-1* (F34D10.6). Boxes represent exons and lines indicate introns. The wild isolate JU1249 has an 8 bp deletion that introduces a frameshift. The *db1082* allele, isolated in a genetic screen for aggregation-defective mutants, replaces a Gln codon with a premature stop codon. (G) Wild-type *arcp-1b* rescues bordering and aggregation phenotypes of JU1249 and *db1082* animals. For each assay, 50–60 animals were transferred to a bacterial lawn and behaviors were scored after 6 h. One-way ANOVA with Tukey test. n ≥ 6 assays. (H) *arcp-1*(*db1082*) animals, like JU1249, fail to suppress CO_2_ responses when acclimated to 21% O_2_. n = 5–6 assays. Two-way ANOVA with Šidák test. (I) Expressing wild-type *arcp-1* restores the O_2_-dependent modulation of CO_2_ responses in *arcp-1; npr-1* mutants. n = 67–105 animals. Mann-Whitney U test. (J) An *arcp-1b* transgene, but not *arcp-1a*, rescues the enhanced locomotory arousal evoked by CO_2_ in *arcp-1; npr-1* animals acclimated to 21% O_2_. n ≥ 4 assays for all genotypes. One-way ANOVA with Tukey test. (K) An *arcp-1b* transgene rescues the enhanced CO_2_ response of JU1249 animals acclimated to 21% O_2_. n = 6 assays. One-way ANOVA with Tukey test. For (H)–(K), each genotype was tested in at least 4 assays with 20–30 animals per trial. Solid lines plot mean; shaded areas show SEM; horizontal black bars indicate time intervals for statistical comparisons; vertical bars plot mean ± SEM. ^∗∗^p < 0.01; ^∗∗∗^p < 0.001; ^∗∗∗∗^p < 0.0001; ns, not significant. See also Figures S3, S4, and S5 and Data S1 and S2.

We sought the genetic changes conferring altered plasticity of CO_2_ responses in JU1249. Besides altering this phenotype, JU1249 exhibited reduced aggregation and bordering behavior on an *E. coli* food lawn compared to other *C. elegans* wild isolates (Figures 2A and 2B). We speculated JU1249 aggregated poorly because increased avoidance of CO_2_ shifted the balance between attraction and repulsion as aerobic animals come together. In this model, the aggregation phenotype, which is easy to score, is linked to altered JU1249 CO_2_ responses.

Before testing this hypothesis, we ruled out the possibility that JU1249 is genetically contaminated by the non-aggregating N2 lab strain, by genotyping the *npr-1*, *glb-5*, and *nath-10* loci, which have acquired polymorphisms during N2 domestication (Duveau and Félix, 2012, McGrath et al., 2009, Persson et al., 2009, Weber et al., 2010). JU1249 exhibited the natural alleles found in other wild isolates at all three loci (Figure S3).

To map the JU1249 aggregation defect, we used a selection-based quantitative trait locus (QTL) mapping approach in which we crossed JU1249 to the aggregating *C. elegans* wild isolate JU2825 (Figure 2A). To find conditions for selection-based QTL mapping, we first defined two treatments that differentially selected for aggregating and solitary animals and performed competition tests between JU1249 and JU2825 under these treatments. Starting with a 50:50% mix of each strain, JU1249 (solitary) outcompeted JU2825 (aggregating) when the populations were transferred by liquid harvest and aliquot (Figure S4A, treatment A), indicating that JU1249 has higher fitness in these conditions than JU2825. When cultivated by transferring an agar chunk from the border of the food lawn, where aggregating animals accumulate (Figure S4A, treatment B), JU2825 outcompeted JU1249, which indicates the aggregation trait in *C. elegans* is selectable. We used treatments A and B as selection regimes on populations of cross-progenies of JU1249 and JU2825 (Figure 2C), sequenced their genomes, and compared allele frequencies of paired replicate populations under the two treatments (Data S1; STAR Methods). Populations selected for aggregation (treatment B) were expected to have higher frequencies of JU2825 alleles at the QTL that affect the variation in aggregation behavior compared to the paired populations (treatment A). Our analysis showed large variation in allele frequencies among replicates, suggesting founder effects due to the moderate population sizes in the first crosses (Figures 2D and S4B). We used two criteria to identify candidate QTL regions associated with the aggregation phenotype. First, we identified regions that show consistent differences in allele frequencies among all replicate pairs for the two treatments (Figures S4B and S4C). Second, we narrowed down these regions by examining replicates for the position of the closest recombination event that was selected (Figure S4D). Based on these criteria, we identified a genomic interval on chromosome III (3361869–4086899 bp) as a candidate region, showing a highly significant difference in allele frequencies among the eight population pairs (Figures 2E and S4C).

The 725 kb QTL region in JU1249 contained 3 polymorphisms in protein-coding genes compared to N2 and JU2825 (Data S1J). An 8 bp deletion (*mfP22*) in the open reading frame of the gene F34D10.6, which we named *arcp-1* (for ankyrin repeat containing protein, see below), stood out as a promising candidate for two reasons. First, *mfP22* is the only polymorphism predicted to abolish protein function (Data S1J and S1K), introducing a frameshift and premature stop codon in both transcripts of the *arcp-1* gene (Figures 2F and S5A). Second, we independently found several alleles of *arcp-1* in a collection of sequenced mutants that suppress aggregation behavior of *npr-1*(*null*) animals, including two that introduced premature stop codons. The number and kind of these alleles made it likely that disrupting *arcp-1* caused an aggregation defect. Consistent with this hypothesis, the aggregation defect of one strain (*db1082* allele) mapped to a 1 Mb interval on chromosome III, centered on *arcp-1* (Figures 2F and S5A). Two mutants from the million mutation project (Thompson et al., 2013), harboring *arcp-1* alleles (*gk856856* and *gk863317*) that introduce premature stop codons, were also defective in aggregation and bordering (Figures S5B and S5C). To show conclusively that mutations in *arcp-1* disrupt aggregation, we performed transgenic rescue experiments. Expressing wild-type *arcp-1* in JU1249 or in *arcp-1*(*db1082*)*; npr-1*(*null*) mutants restored aggregation and bordering behavior (Figure 2G).

To gain insight into the distribution of the *arcp-1*(*mfP22*) polymorphism in *C. elegans*, we examined other wild isolates. Our analysis suggests *mfP22* is a rare allele, because we did not find it in a set of 151 worldwide *C. elegans* isolates, including MY16 (Data S2).

Does disrupting *arcp-1* alter responses to CO_2_? *arcp-1*(*db1082*)*; npr-1*(*null*) animals behaved like JU1249: they showed no overt defect in their response to a 21%-to-7% O_2_ downshift (Figure S5D) but failed to suppress escape from different CO_2_ concentrations when acclimated to 21% O_2_ (Figures 2H, S5E, and S5F). A wild-type *arcp-1* transgene rescued this CO_2_ plasticity defect (Figure 2I). *arcp-1* is thus required for animals acclimated to 21% O_2_ to suppress escape from high CO_2_ environments.

Gene predictions and cDNA cloning revealed *arcp-1a* and *arcp-1b* transcripts that overlap at their 3′ end (Figure 2F; Wormbase WS265). The *db1082* and *mfP22* alleles affect both *arcp-1* transcripts (Figure 2F). Expressing *arcp-1b* fully rescued the heightened CO_2_ response of these animals, whereas a transgene for the longer *arcp-1a* transcript did not (Figures 2J and 2K). A mutation that only disrupted *arcp-1a* also failed to recapitulate the enhanced CO_2_ response and aggregation phenotype of mutants defective in both *arcp-1* transcripts (Figures S5C and S5G). Thus *arcp-1*, and more specifically the product of its *b* transcript, is required for animals to suppress CO_2_ escape following acclimation to 21% O_2_.

### ARCP-1 Acts in BAG Sensory Neurons to Suppress CO_2_ Escape Behavior

**Figure 3:**
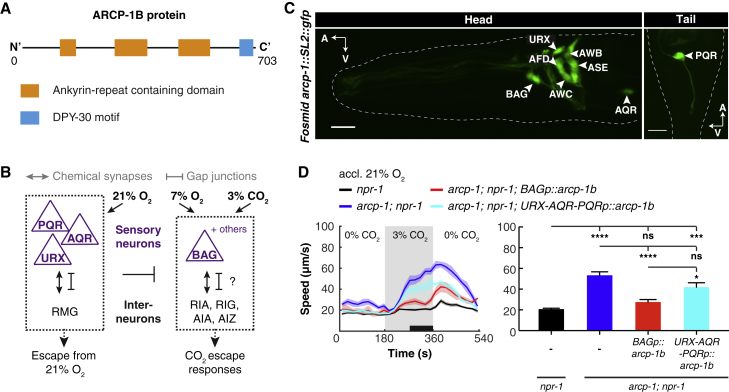
ARCP-1B Acts in BAG Sensors to Suppress CO_2_ Escape Behavior (A) Protein domain architecture of ARCP-1B. (B) Schematic model of the core neural circuits for O_2_ and CO_2_ responses in *C. elegans* (Fenk and de Bono, 2017, Guillermin et al., 2017, Laurent et al., 2015). O_2_-sensing neurons URX, AQR, and PQR tonically signal 21% O_2_. CO_2_ stimuli and O_2_ downshifts are detected by BAG and other neurons. The O_2_ sensors cross-modulate the neural circuit underlying CO_2_ escape. The role of RIA, RIG, AIA, and AIZ in the CO_2_ circuit is hypothesized based on their function in CO_2_ aerotaxis (Guillermin et al., 2017). (C) A fosmid reporter transgene for *arcp-1* is expressed in all major O_2_ and CO_2_ sensors, and other sensory neurons. Scale bar, 10 μm; A, anterior; V, ventral. (D) Cell-specific expression of *arcp-1b* in BAG, using the *flp-17* promoter (*BAGp*), rescues locomotory arousal by CO_2_, whereas expression in URX, AQR, and PQR, using the *gcy-32* promoter (*URX-AQR-PQRp*), does not. One-way ANOVA with Tukey test. n ≥ 5 assays with 20–30 animals per trial. ^∗^p < 0.05; ^∗∗∗^p < 0.001; ^∗∗∗∗^p < 0.0001; ns, not significant. See also Figures S5 and S6.

*arcp-1* encodes an ankyrin repeat protein (Figure 3A) homologous to *C. elegans* ankyrin UNC-44 and vertebrate ankyrins (Otsuka et al., 1995). These proteins are important for the subcellular localization of neural signaling complexes (e.g., anchoring components of the axon initial segment and allowing cyclic nucleotide-gated channels to accumulate in photoreceptor cilia) (Kizhatil et al., 2009, Leterrier et al., 2017, Maniar et al., 2011). Besides ankyrin repeats, ARCP-1 contains a DPY-30 domain (Figure 3A). Both domains are common protein interaction motifs that regulate the function and spatial organization of diverse signaling complexes (Gopal et al., 2012, Jones and Svitkina, 2016, Monteiro and Feng, 2017, Sivadas et al., 2012). ARCP-1’s domain structure suggests it serves a similar role trafficking or localizing signaling proteins in the nervous system.

A fosmid-based bicistronic transgene that co-expressed *arcp-1* and free GFP was expressed in the main CO_2_ and O_2_ sensors: the URX, AQR, PQR, and BAG neurons (Figures 3B and 3C). We also observed expression in a subset of other sensory neurons (i.e., AFD, ASE, AWC, and AWB) (Figure 3C). This raised the possibility that disrupting *arcp-1* modifies plasticity in multiple paradigms. To test this, we assayed *arcp-1* mutants in a salt-based associative learning paradigm (Figure S6A; Beets et al., 2012, Hukema et al., 2008). *arcp-1* mutants were defective in gustatory plasticity: although mock-conditioned animals showed normal attraction to NaCl, upon salt conditioning they failed to downregulate salt chemotaxis behavior (Figure S6B).

To gain insight into *arcp-1* function, we focused on the failure of *arcp-1* mutants to suppress CO_2_ escape when acclimated to 21% O_2_. Because *arcp-1* is expressed in the BAG CO_2_ sensors, we asked if it acts in these neurons to suppress CO_2_ escape. Cell-specific expression of wild-type *arcp-1* in BAG using the *flp-17* promoter (Kim and Li, 2004) rescued the increased locomotory activity of *arcp-1* mutants at 3% CO_2_ (Figure 3D). We also tested if *arcp-1* can act in URX, AQR, and PQR neurons, which sense 21% O_2_, to suppress CO_2_ escape. Expressing *arcp-1* in these neurons, using the *gcy-32* promoter (Yu et al., 1997), did not rescue the CO_2_ phenotype of *arcp-1* mutants (Figure 3D). By contrast, the *arcp-1* aggregation defect could be rescued by expressing *arcp-1* either in BAG or in URX, AQR, and PQR (Figures S5H and S5I). Together, these data show that *arcp-1* functions in gas-sensing neurons and cell-autonomously suppresses CO_2_ escape in the BAG CO_2_ sensors.

### BAG Responses to CO_2_ Are Tuned by ARCP-1

**Figure 4:**
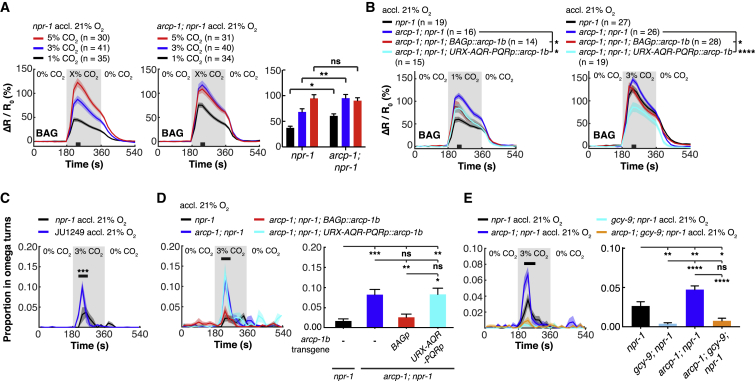
ARCP-1 Suppresses BAG Responses to CO_2_ (A and B) Mean traces of BAG Ca^2+^ activity in *npr-1* and *arcp-1; npr-1* animals in response to different CO_2_ concentrations. Mutants for *arcp-1* show increased Ca^2+^ activity at 1% and 3% CO_2_ (A), which is rescued by expressing *arcp-1* either in BAG (*flp-17p*) or URX, AQR, and PQR (*gcy-32p*) (B). n = number of animals. Two-way ANOVA with Šidák test in (A). One-way ANOVA with Holm-Šidák test in (B). (C–E) CO_2_-evoked turning behavior. (C) Rising CO_2_ levels stimulate stronger turning behavior in JU1249 (n = 85 animals) than in *npr-1*(*215F*) animals (n = 81). Mann-Whitney U test. (D) CO_2_-evoked turning is also increased in *arcp-1*(*db1082*)*; npr-1*(*ad609*) animals. BAG-specific expression of a *flp-17p::arcp-1b* transgene rescues this phenotype, whereas expression of *arcp-1b* in URX, AQR, and PQR (*gcy-32p*) does not. One-way ANOVA with Tukey test. n ≥ 5 assays with 20–30 animals per trial. (E) The increased turning of *arcp-1; npr-1* animals in response to CO_2_ requires the GCY-9 CO_2_ receptor. One-way ANOVA with Tukey test. n = 9 assays with 20–30 animals per trial. For (A)–(E), solid lines plot mean; shaded areas show SEM; black bars indicate time intervals for statistical comparisons; bar graphs plot mean ± SEM for these intervals. ^∗^p < 0.05; ^∗∗^p < 0.01; ^∗∗∗^p < 0.001; ^∗∗∗∗^p < 0.0001; ns, not significant. See also Figure S6.

We investigated if the increased behavioral response of *arcp-1* animals to CO_2_ was associated with increased CO_2_-evoked Ca^2+^ responses in BAG neurons. Using the ratiometric sensor YC3.60, we quantified fluorescence changes at the cell body of BAG in response to CO_2_. Animals acclimated to 21% O_2_ were transferred to a microfluidic chamber kept at 7% O_2_ and stimulated with different CO_2_ concentrations. BAG Ca^2+^ responses evoked by 1% and 3% CO_2_ were significantly higher in *arcp-1* mutants compared to controls (Figure 4A). Unlike for CO_2_ escape, expressing *arcp-1* either in BAG or in URX, AQR, and PQR rescued the CO_2_ Ca^2+^ phenotype in BAG (Figure 4B). At 3% CO_2_, animals with *arcp-1* rescued in URX, AQR, and PQR even showed a smaller increase in Ca^2+^ activity compared to *npr-1* controls, which may be due to an overexpression effect of the *gcy-32p::arcp-1b* transgene (Figure 4B). Because BAG neurons exhibit a phasic-tonic response to CO_2_, we also measured Ca^2+^ responses during prolonged CO_2_ stimulation. BAG tonic responses to 3% CO_2_ were reduced in *arcp-1* mutants, although the effect was small (Figure S6C).

BAG neurons respond not only to a rise in CO_2_, but also to a fall in O_2_ (Zimmer et al., 2009). We asked if the CO_2_ phenotypes of *arcp-1* animals could be indirectly linked to changes in BAG’s ability to respond to O_2_. BAG Ca^2+^ activity at 7% O_2_, measured by YC2.60, was similar for *arcp-1* mutants and *npr-1* controls, although *arcp-1* animals displayed higher Ca^2+^ at 21% O_2_ (Figure S6D). Ca^2+^ responses in URX to a 7% to 21% O_2_ stimulus were unaffected in *arcp-1* animals (Figure S6E).

BAG O_2_ responses are mediated by the guanylate cyclases GCY-31/GCY-33 and are abolished in mutants of these genes (Zimmer et al., 2009). Animals lacking *gcy-33* and *gcy-31*, like *arcp-1* mutants, were aroused more strongly by CO_2_, but the effects on CO_2_ escape were additive in an *arcp-1; gcy-33; gcy-31; npr-1* quadruple mutant (Figure S6F). Moreover, in *gcy-33; gcy-31* mutants, CO_2_ arousal was suppressed when animals were acclimated to 21% O_2_—unlike in *arcp-1* animals (Figure S6G). These results indicate that *arcp-1* can act in a separate genetic pathway from *gcy-33* and *gcy-31* to regulate CO_2_ escape. Together with our rescue and Ca^2+^ imaging data, these findings are consistent with *arcp-1* suppressing CO_2_ escape by inhibiting BAG responses to CO_2_.

### ARCP-1 Inhibits BAG-Mediated Turning Downstream of the CO_2_ Receptor GCY-9

*C. elegans* respond to a rise in CO_2_ not only by becoming aroused and moving faster but also by re-orienting their direction of travel and increasing the frequency of sharp (omega) turns. This behavior is also mediated by BAG (Fenk and de Bono, 2015, Hallem and Sternberg, 2008). Because ARCP-1 acts in BAG to suppress CO_2_-evoked Ca^2+^ responses and locomotory arousal, we asked if it also inhibits CO_2_-evoked turning. Both *arcp-1* mutants and JU1249 showed increased turning in response to a rise in CO_2_ compared to controls (Figures 4C and 4D). This phenotype was rescued by expressing *arcp-1* in BAG, but not by expressing it in URX, AQR, and PQR (Figure 4D).

To gain insight into the molecular functions of *arcp-1*, we examined its effect on CO_2_-evoked turns further. This part of the locomotory response to CO_2_ is driven by cGMP signaling from the guanylyl cyclase receptor GCY-9 in BAG neurons (Fenk and de Bono, 2015, Hallem et al., 2011). GCY-9 is a molecular receptor for CO_2_ and appears to be specifically expressed in BAG (Hallem et al., 2011, Smith et al., 2013). To examine if *arcp-1* regulates turning downstream of GCY-9, we measured CO_2_-evoked turns in a *gcy-9; arcp-1* mutant. Disrupting *gcy-9* abolished turning evoked by 3% CO_2_ in both *npr-1* and *arcp-1; npr-1* animals (Figure 4E), which implies that the mutant’s turning phenotype depends on GCY-9, and ARCP-1 antagonizes GCY-9 signaling in BAG.

### ARCP-1 Localizes Phosphodiesterase PDE-1 to BAG Cilia

**Figure 5:**
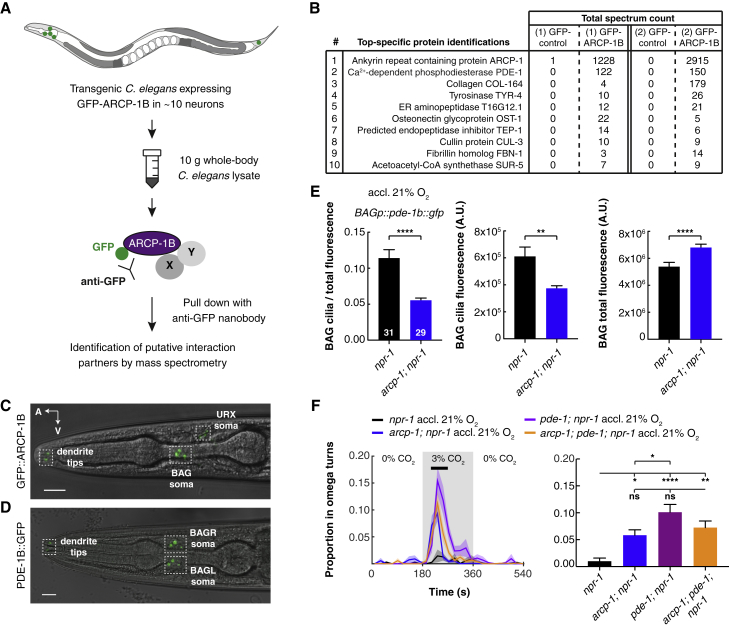
ARCP-1 Is a Scaffolding Protein that Localizes Phosphodiesterase PDE-1 to Dendritic Endings (A) Schematic of coimmunoprecipitation (coIP) approach to identify ARCP-1B interactors, by pull-down of an N-terminal GFP tag. (B) Top ten specific putative interactors of GFP-ARCP-1B identified in two independent coIPs. IPs of other cytoplasmic GFP-tagged proteins provide negative controls. (C and D) GFP-tagged ARCP-1B and PDE-1B proteins are both enriched at the sensory endings of BAG. Scale bar, 10 μm; A, anterior; V, ventral. (E) Disrupting *arcp-1* reduces enrichment of PDE-1, expressed from the *flp-17p*, at BAG cilia. Bars plot mean ± SEM n (in bars) = number of animals. Mann-Whitney U test. (F) *pde-1* mutants phenocopy the increased turning frequency of *arcp-1* mutants in response to CO_2_. *pde-1; arcp-1* double mutants do not show an additive phenotype. Solid lines plot mean; shaded areas show SEM; black bars indicate time intervals for statistical comparisons; bar graphs plot mean ± SEM for these intervals. One-way ANOVA with Tukey test. n ≥ 8 assays with 20–30 animals per trial. ^∗^p < 0.05; ^∗∗^p < 0.01; ^∗∗∗∗^p < 0.0001; ns, not significant. See also Figure S7 and Data S3.

The ankyrin repeats and DPY-30 motif of ARCP-1 suggest it serves as an interaction partner or scaffold for other proteins. To identify its molecular partners, we took a biochemical approach (Figure 5A). We first made a transgenic strain that expressed GFP-ARCP-1B and showed that it rescued the enhanced CO_2_ response of the *arcp-1* mutant (Figure S7A). We then used anti-GFP nanobodies to pull down GFP-ARCP-1B fusion proteins from *C. elegans* lysates and identified putative interacting proteins by mass spectrometry (Figure 5A; Data S3). As a negative control, we immunoprecipitated other GFP-tagged cytoplasmic proteins in parallel. Across two independent experiments, we identified phosphodiesterase 1 (PDE-1) as the top specific hit (i.e., the protein having the highest number of spectral counts in ARCP-1B immunoprecipitates [IPs] while having none in control IPs) (Figure 5B; Data S3).

PDE-1 is a Ca^2+^-activated cyclic guanosine monophosphate (cGMP)/cyclic AMP (cAMP) phosphodiesterase orthologous to mammalian Ca^2+^/calmodulin-dependent PDE1 and is expressed in many neurons, including BAG (Couto et al., 2013, Hallem et al., 2011). As expected from our biochemical data, PDE-1 and ARCP-1 localize to similar compartments in BAG. GFP-tagged ARCP-1B was enriched at sensory endings (Figure 5C), similar to what we observe and what has been reported for PDE-1 (Martínez-Velázquez and Ringstad, 2018; Figure 5D).

The biochemical interaction of ARCP-1 and PDE-1, and their co-localization at dendritic endings, led us to hypothesize that ARCP-1 regulates PDE-1 localization. To test this, we compared enrichment of PDE-1 at BAG cilia in *arcp-1* and control animals. Overall, PDE-1 expression was slightly higher in *arcp-1* mutants, but enrichment of PDE-1 at the cilia was reduced by more than half in these animals (Figure 5E). To extend this observation, we investigated the subcellular localization of other signaling components of the gas-sensing neurons in *arcp-1* mutants. We observed a reduction of GCY-9 levels in BAG cilia, as well as reduced levels of the O_2_-sensing guanylate cyclase GCY-35 at the sensory endings of URX (Figures S7B and S7C). These phenotypes were not due to a general defect in dendritic localization, because *arcp-1* mutants showed normal levels of the cGMP-gated channel subunit TAX-4 and the O_2_-sensing guanylate cyclase GCY-33 in BAG cilia (Figures S7D and S7E). *arcp-1* mutants did not exhibit overt defects in dendritic morphology, based on expression of a *flp-17p::gfp* transgene and DiI filling of amphid sensory neurons (Figures S7F and S7G). Together, our data suggest that ARCP-1 acts as a scaffold that helps co-localize signal transduction components at sensory endings of some neurons.

Our behavioral, Ca^2+^ imaging and cell biological results led us to speculate that ARCP-1 promotes a Ca^2+^-dependent feedback mechanism mediated by PDE-1, which keeps BAG CO_2_ responses in check by degrading cGMP following activation of the CO_2_ receptor GCY-9. If this is correct, disrupting *pde-1* should phenocopy *arcp-1* and increase the frequency of CO_2_-evoked turns. Moreover, the *arcp-1* and *pde-1* phenotypes should not be additive. As predicted, *pde-1* mutants turned more in response to 3% CO_2_ than controls and even *arcp-1* mutants, likely because *pde-1* is more widely expressed and serves broader functions than *arcp-1*. The turning phenotype was comparable for *pde-1*, *arcp-1*, and *pde-1; arcp-1* mutants (Figure 5F). These results are consistent with *pde-1* and *arcp-1* acting in the same genetic pathway to keep CO_2_ responses in check.

### PDE-1 and ARCP-1 Inhibit Expression of FLP-19 Neuropeptides

To investigate further how disrupting *arcp-1* alters BAG function, we specifically labeled these neurons with GFP, used fluorescence-activated cell sorting (FACS) to isolate the fluorescent cells from acutely dissociated *arcp-1;npr-1* and *npr-1* control animals, and profiled their gene expression using RNA sequencing (RNA-seq) (see STAR Methods). Genes that are hallmarks of BAG, such as those involved in CO_2_ signaling (*gcy-9*, *pde-1*, *flp-17*) and BAG cell fate determination (*ets-5*) (Guillermin et al., 2011, Hallem et al., 2011), were among the top enriched genes in our dataset (Data S4). *arcp-1* itself was among the 100 most highly expressed genes in BAG. The BAG profiles highlighted significant gene expression differences between *arcp-1* mutants and controls, notably changes in the abundance of mRNAs encoding neuropeptides, genes involved in ciliary intraflagellar transport, ion channels, and gap junction subunits (see Discussion; Data S4D). These data suggest that loss of ARCP-1 leads to altered gene expression.

**Figure 6:**
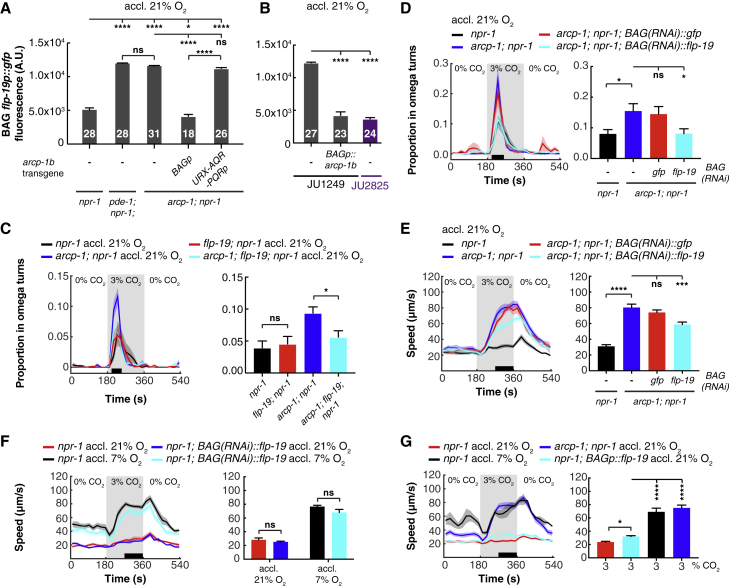
PDE-1 and ARCP-1 Inhibit BAG Expression of FLP-19 Neuropeptides that Potentiate Behavioral Responses to CO_2_ (A) Mean fluorescence ± SEM of a *flp-19* neuropeptide reporter (*flp-19p::gfp*) in BAG, indicating that PDE-1 and ARCP-1 inhibit *flp-19* expression. BAG-specific expression of *arcp-1b*, using the *flp-17* promoter (*BAGp*), rescues this phenotype, whereas expression in URX, AQR and PQR, using the *gcy-32* promoter (*URX-AQR-PQRp*), does not. n (in bars) = number of animals. One-way ANOVA with Tukey test. (B) Mean fluorescence ± SEM of *flp-19* neuropeptide reporter in BAG neurons of JU1249 and JU2825. Increased expression of *flp-19* in JU1249 is rescued by expressing *arcp-1b* from the BAG-specific *flp-17* promoter (*BAGp*). n (in bars) = number of animals. Kruskal-Wallis with Dunn test. (C) Disrupting *flp-19* suppresses the potentiated turning phenotype of *arcp-1; npr-1* animals in response to 3% CO_2_. One-way ANOVA with Holm Šidák test. n = 9 assays. (D) CO_2_-evoked turning of *arcp-1; npr-1* mutants following cell-specific knock down of *flp-19* expression in BAG. Knock down of *flp-19* in the mutant background suppresses turning at 3% CO_2_, whereas knock down of *gfp* does not. One-way ANOVA with Dunnett’s test. n ≥ 7 assays. (E) Knock down of *flp-19* expression in BAG partially rescues the increased arousal phenotype of *arcp-1; npr-1* animals at 3% CO_2_. One-way ANOVA with Dunnett’s test. n ≥ 7 assays with 20–30 animals per trial. (F) BAG-specific knock down of *flp-19* in *npr-1* animals does not affect the plasticity of CO_2_ escape in response to previous O_2_ experience. Two-way ANOVA with Šidák test. n = 7–8 assays. (G) Animals overexpressing *flp-19* in BAG move significantly faster at 3% CO_2_ compared to *npr-1* controls, although their response is still lower than *npr-1* animals grown at 7% O_2_ and *arcp-1* mutants. n ≥ 3 assays. One-way ANOVA with Tukey test. For (C)–(G), 20–30 animals were tested per assay. Solid lines plot mean; shaded areas show SEM; black bars indicate time intervals for statistical comparisons; bars plot mean ± SEM for these intervals. ^∗^p < 0.05; ^∗∗∗^p < 0.001; ^∗∗∗∗^p < 0.0001; ns, not significant. See also Figure S7 and Data S4.

One of the most abundant transcripts expressed in BAG whose expression was significantly altered by defects in *arcp-1* was the neuropeptide *flp-19*. *flp-19* expression was upregulated 2.4-fold in *arcp-1* animals, which would be consistent with increased BAG signaling. Previous work has shown that GCY-9, PDE-1, and the cGMP-gated Ca^2+^ channel TAX-4 control *flp-19* expression in BAG (Romanos et al., 2017), making it an interesting candidate for altering CO_2_ responses in the *arcp-1* mutant. To confirm that defects in *arcp-1* increased expression of *flp-19*, we introduced a *flp-19p::gfp* reporter transgene (Kim and Li, 2004) into *arcp-1* mutants and quantified fluorescence in BAG neurons. Disrupting *arcp-1* significantly increased BAG expression of the neuropeptide reporter (Figure 6A). This phenotype was rescued by expressing wild-type *arcp-1* in BAG, but not in the O_2_ sensors URX, AQR, and PQR (Figure 6A). Thus, *arcp-1* controls *flp-19* expression cell-autonomously in BAG. We observed a similar increase in expression of the *flp-19* reporter when *pde-1* was mutated (Figure 6A). BAG expression of *flp-19* in mutants lacking both *arcp-1* and *pde-1* was similar to that of the single mutants (Figure S7H). These data suggest that ARCP-1 and PDE-1 together reduce BAG signaling by lowering the expression of some neuropeptides. However, disrupting *arcp-1* does not generally increase BAG neuropeptide expression as judged from our BAG profiling experiments (Data S4) and analysis of a *flp-17* neuropeptide reporter in BAG (Figure S7I).

To ask if *flp-19* expression was elevated in JU1249, we backcrossed the *flp-19p::gfp* transgene ten times to this isolate. We did the same for JU2825 that, unlike JU1249, suppressed CO_2_ escape when acclimated to 21% O_2_ (Figure S2A). BAG expression of *flp-19* was low in JU2825 and high in JU1249 (Figure 6B). Restoring *arcp-1* in BAG significantly reduced *flp-19* expression (Figure 6B). Thus, disrupting *arcp-1* also increases *flp-19* expression in JU1249.

### FLP-19 Neuropeptide Signaling from BAG Potentiates Behavioral Responses to CO_2_

Does increased BAG expression of *flp-19* in *arcp-1* mutants enhance the behavioral responses of these animals to CO_2_? If increased *flp-19* expression heightened aversion to CO_2_ in *arcp-1* animals, then disrupting *flp-19* should reverse this phenotype. Consistent with this hypothesis, deleting *flp-19* restored turning at 3% CO_2_ in the *arcp-1* mutant, while it had no effect on this behavior in *npr-1* animals (Figure 6C).

To confirm that FLP-19 release from BAG potentiates CO_2_ responses, we knocked down *flp-19* expression specifically in these neurons by expressing RNAi sense and antisense sequences of *flp-19* from a BAG-specific *gcy-33* promoter (Hallem et al., 2011, Yu et al., 1997). As a negative control, we expressed sense and antisense sequences for *gfp* under the same promoter and found it had no effect on CO_2_ responses (Figures 6D and 6E). By contrast, BAG-specific knockdown of *flp-19* in *arcp-1* mutants restored the frequency of CO_2_-evoked turns (Figure 6D) and reduced CO_2_-evoked locomotory arousal in animals acclimated to 21% O_2_ (Figure 6E). These data suggest increased *flp-19* expression in BAG contributes to the enhanced behavioral responses to CO_2_ in *arcp-1* mutants.

The neuropeptide gene *flp-19* is also expressed in URX. However, knock down of *flp-19* in these neurons, using the *gcy-32* promoter, enhanced rather than reduced locomotory arousal at 3% CO_2_ in *arcp-1* animals and increased baseline locomotion in the absence of CO_2_ (Figure S7K). This result is consistent with previous reports (Carrillo et al., 2013) and suggests that the RNAi effect in BAG is specific to these neurons. We wondered if altered expression of *flp-19* from URX contributes to the enhanced CO_2_ aversion in *arcp-1* animals as well. If this is the case, *flp-19* expression in URX should be reduced in *arcp-1* mutants. Indeed, disrupting *arcp-1* decreased expression of the *flp-19* reporter in URX. This phenotype was rescued by expressing *arcp-1* either in URX or BAG neurons (Figure S7L), suggesting that BAG signaling indirectly influences *flp-19* expression in URX.

Does FLP-19 release from BAG promote escape from CO_2_ in animals that retain functional *arcp-1*? In *npr-1* animals, BAG-specific knock down of *flp-19* did not compromise CO_2_ escape in animals acclimated to 21% O_2_ or 7% O_2_ (Figure 6F). Thus, *flp-19* is not required for the O_2_-dependent modulation of CO_2_ responses. Consistent with this finding, we observed similar expression of the *flp-19* reporter in *npr-1* animals acclimated to 21% or 7% O_2_, suggesting that *flp-19* expression is not regulated by O_2_ experience (Figure S7J).

We next asked if increased *flp-19* expression in BAG is sufficient to boost *C. elegans*’ locomotory arousal by CO_2_. To test this, we overexpressed *flp-19* specifically in the BAG neurons of *npr-1* animals, acclimated these transgenic animals to 21% O_2_, and quantified their speed at 3% CO_2_. Animals overexpressing *flp-19* in BAG moved significantly faster at 3% CO_2_ than *npr-1* controls, although their locomotory arousal was weaker than that of *arcp-1* animals or of *npr-1* animals grown at 7% O_2_ (Figure 6G). Thus, acclimation to 7% O_2_ or disrupting *arcp-1* alters other signals besides *flp-19* to heighten CO_2_ responses. However, in both scenarios—disruption of *arcp-1* or O_2_ acclimation—increasing *flp-19* expression in BAG can potentiate behavioral responses to CO_2_, leading to increased CO_2_ aversion.

## Discussion

Individuals differ in how they respond to altered circumstances in their environment. This is generally ascribed to a combination of genetic variation and different life experiences. How neural circuits encoding behavioral plasticity vary across individuals is, however, poorly understood. Here, we show that *Caenorhabditis* species and wild isolates of *C. elegans* can differ in how past O_2_ experience influences CO_2_ escape behavior. We uncover a genetic variant and neuronal mechanism responsible for this variation in behavioral flexibility in one natural *C. elegans* isolate.

**Figure 7:**
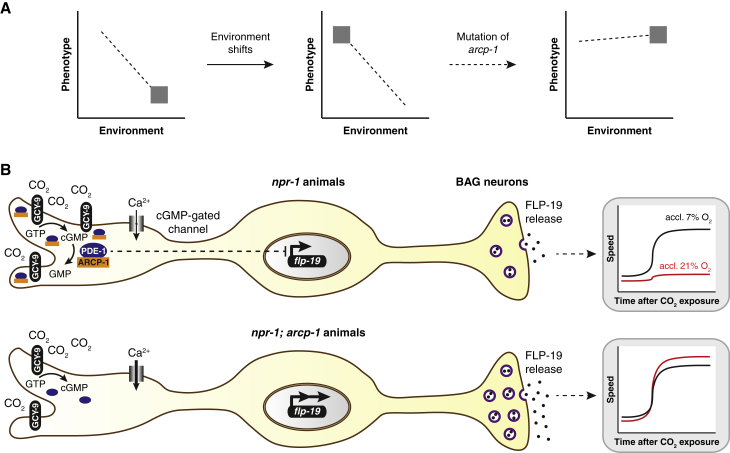
A Model for How Genetic Variation in *arcp-1* Affects CO_2_ Escape Behavior (A) Effect of the natural *arcp-1* allele on experience-dependent plasticity, shown as behavioral reaction norms. *C. elegans* wild isolates acclimated to a high (21%) O_2_ environment suppress their aversion to CO_2_ (left panel). A shift to a low (7%) O_2_ environment results in a heightened CO_2_ response. A mutation in *arcp-1* alters experience-dependent plasticity and genetically fixes a strong aversive response to CO_2_ in part by increasing *flp-19* neuropeptide expression in BAG CO_2_ sensors (right panel). (B) CO_2_ is detected by the receptor guanylate cyclase GCY-9, expressed in BAG cilia. The ankyrin-repeat scaffold protein ARCP-1 is also enriched at dendritic sensory endings, interacts with PDE-1, and localizes this phosphodiesterase to the cilia of BAG CO_2_-sensory neurons. PDE-1 and ARCP-1 inhibit CO_2_-evoked Ca^2+^ activity and expression of FLP-19 neuropeptide messengers in BAG. In the absence of ARCP-1, less GCY-9 and PDE-1 localize to BAG cilia, and *flp-19* is more strongly expressed. Increased FLP-19 expression in BAG contributes to resetting a strong aversive response to CO_2_ in *arcp-1; npr-1* animals regardless of previous O_2_ experience.

The behavioral phenotypes that we observe are reminiscent of genetic accommodation, when the reaction norm of a flexible phenotype responsive to the environment is altered by genetic change (Figure 7A). Underlying this behavioral change, we find that disrupting ARCP-1 both increases CO_2_ sensitivity and alters the effect of previous O_2_ experience on CO_2_ escape. Animals lacking this dendritic scaffold protein become strongly aroused by CO_2_ regardless of previous O_2_ experience, and acclimation to 21% O_2_ further enhances, rather than suppresses, escape from this aversive cue. We show that loss of *arcp-1* mediates these phenotypes by directly altering CO_2_ responses, rather than by affecting the ability to respond to O_2_.

We identify the BAG CO_2_ sensors as the main site where ARCP-1 suppresses CO_2_ escape in animals acclimated to 21% O_2_. Together with previous work (Couto et al., 2013, Romanos et al., 2017), our results suggest a model (Figure 7B) in which ARCP-1 binds and co-localizes the Ca^2+^-activated phosphodiesterase PDE-1 with guanylyl cyclase receptors for CO_2_ at the BAG cilia. ARCP-1 and PDE-1 keep signaling from these neurons in check by suppressing CO_2_-evoked Ca^2+^ responses and neuropeptide expression. Natural genetic variation has been found to directly alter sensory systems in other animals (McGrath, 2013, Prieto-Godino et al., 2017). We identify BAG as a major cellular focus for variation in CO_2_ responses, but the possibility remains that loss of *arcp-1* disrupts plasticity in other sensory circuits, which may indirectly promote CO_2_ aversion as well. Some evidence points to changes in URX, but these are not sufficient to explain the heightened CO_2_ escape behavior in *arcp-1* mutants.

Mounting evidence suggests that natural variation in behavioral flexibility is genetically determined (Izquierdo et al., 2007, Mery, 2013, Mery et al., 2007). One well-established example is the natural variation seen at the *foraging* gene in *Drosophila melanogaster*. This polymorphism causes individual variation in learning and memory, among other phenotypes, by altering the activity of cGMP-dependent protein kinase G (Mery et al., 2007). It is notable that, both in flies and worms, genetic variation affecting cGMP signaling underlies inter-individual variation in experience-dependent plasticity. Besides gas sensors, ARCP-1 is expressed in olfactory, gustatory, and thermosensory neurons that all signal using cGMP, and *arcp-1* mutants show reduced plasticity in a gustatory paradigm. Correlated differences in the plasticity of different sensory modalities have been described as coping styles or behavioral syndromes in other animal models (Coppens et al., 2010) and may also reflect a common genetic or molecular basis. Identifying how loss of *arcp-1* compromises plasticity in other sensory circuits should provide a better understanding of such correlated changes in behavioral flexibility.

We have shown that the absence of ARCP-1 alters expression of a range of genes in BAG. One way this influences CO_2_ aversion is by altering the expression of neuropeptide messengers. Neuropeptides are a diverse group of neuromodulators that, both in vertebrates and invertebrates, are involved in circuit plasticity (Jékely et al., 2018, Taghert and Nitabach, 2012). Natural genetic variation in neuropeptide pathways has been linked to individual differences in aging and social behaviors (Donaldson and Young, 2008, Yin et al., 2017). Our results suggest that they also contribute to heritable differences in behavioral plasticity between individuals. In humans and other primates, natural polymorphisms in serotonergic and dopaminergic systems have been associated with individual differences in memory and cognitive ability (Izquierdo et al., 2007, Zhang et al., 2007). Changing the neuromodulatory tone of circuits likely represents a general mechanism by which genetic variation sculpts individual behavioral plasticity.

Disrupting ARCP-1 increases expression of FLP-19 neuropeptides in BAG. This potentiates or disinhibits both CO_2_-evoked turning and locomotory arousal in animals acclimated to 21% O_2_. A FLP-19 receptor is currently unknown; the *C. elegans* genome encodes ∼150 predicted neuropeptide receptors but none have been reported to bind FLP-19 (Peymen et al., 2014). FLP-19 neuropeptides belong to the ancient and conserved family of RFamide neuropeptides (Peymen et al., 2014). Previous work suggested that CO_2_-evoked cGMP and Ca^2+^ signaling promote *flp-19* expression in BAG, and this effect is counterbalanced by PDE-1 (Romanos et al., 2017). In *arcp-1* mutants, the GCY-9 CO_2_ receptor and PDE-1 are less enriched at BAG cilia. Although *gcy-9* expression is slightly reduced, disrupting ARCP-1 increases BAG Ca^2+^ activity in response to CO_2_. This is consistent with proper ciliary localization of PDE-1 keeping BAG Ca^2+^ signaling in check and could explain the increased *flp-19* expression. ARCP-1 and PDE-1 may also orchestrate microdomains of cGMP that can regulate gene expression (Arora et al., 2013, O’Halloran et al., 2012). In vertebrate neurons, nanodomains of the ankyrin G protein, a homolog of ARCP-1, localize to the dendritic spines and the axon initial segment and contribute to neural plasticity (Grubb and Burrone, 2010, Smith et al., 2014). Likewise, mammalian PDE1 has been implicated in the experience-dependent adaptation of sensory responses. In mouse olfactory neurons, PDE1 is specifically enriched at the cilia, although a molecular anchor that localizes the protein to this compartment has not yet been identified (Cygnar and Zhao, 2009).

The molecular mechanism by which ARCP-1 controls *flp-19* expression, and whether this relates to its ciliary function, remains to be understood. Interestingly, our transcriptional profiling of BAG neurons revealed a suite of genes involved in intraflagellar transport, including the axonemal dynein *che-3*, that show ∼2-fold increased expression in *arcp-1* animals, although we did not observe obvious defects in cilia morphology. This suggests a feedback mechanism exists by which signaling at the cilium regulates expression of genes involved in ciliary transport. Identifying the molecular factors involved is the next step forward toward understanding this transcriptional regulation.

The mechanisms through which natural genetic variation in *arcp-1*, acting on an evolutionary timescale, and O_2_ experience, acting over an animal’s lifetime, sculpt CO_2_ responsiveness seem to be at least partly distinct. However, in both scenarios—disruption of *arcp-1* or acclimation to different O_2_ environments—release of FLP-19 neuropeptides from BAG can boost the animal’s response to this aversive cue, and through alterations in neuropeptide expression, a strong aversive response may become fixed.

CO_2_ responses vary between *Caenorhabditis* species (Carrillo and Hallem, 2015, Pline and Dusenbery, 1987, Viglierchio, 1990). Our results show this variation is at least in part due to differences in O_2_-dependent modulation, suggesting it is an adaptive trait. We speculate that the influence of O_2_ memory on other sensory responses enables animals to reconfigure their behavioral priorities according to past experience (Fenk and de Bono, 2017). Animals at the surface may prioritize escape from 21% O_2_ and gradually suppress their CO_2_ aversion to facilitate migration to buried environments with less aeration and elevated CO_2_ levels. Natural variation in the O_2_-dependent modulation of CO_2_ escape may result in animals occupying different ecological niches. Alternatively, there could be selection against the costs to maintain sensory systems for behavioral plasticity (Dewitt et al., 1998), which may account for the reduced plasticity of CO_2_ responses in some nematode species. We do not know the O_2_ and CO_2_ conditions in which the *arcp-1* deletion may have been selected and can therefore only speculate about its potential fitness benefits. The *arcp-1* mutation was not found in any other wild isolate so is likely recent. However, our data indicate substantial variation among both *Caenorhabditis* species and *C. elegans* isolates in the response to CO_2_ (Figures 1 and S2). These findings are consistent with what has been found for other traits (Frézal et al., 2018), where the phenotypic variation for the strain chosen for study is caused by a rare allele found only in that strain, yet phenotypic variation itself is not restricted to that strain. Understanding evolutionary mechanisms that might select for altered plasticity requires more in-depth knowledge on the ecology of these species. The behavioral phenotypes that we observe are consistent with genetic accommodation for a cross-modal gene-environment interaction (Pigliucci et al., 2006). In summary, our study illustrates how natural genetic variation, by altering the neuromodulatory control of aversive behavior, contributes to individual differences in behavioral flexibility.

## STAR★Methods

### Key Resources Table

**Table:** 

REAGENT or RESOURCE	SOURCE	IDENTIFIER
**Antibodies**		

GFP-Trap Agarose	ChromoTek	Cat#gta-20; RRID: AB_2631357

**Bacterial and Virus Strains**		

*E. coli:* Strain OP50	*Caenorhabditis* Genetics Center	Wormbase: OP50; RRID: WB-STRAIN:OP50

**Chemicals, Peptides, and Recombinant Proteins**		

Dermabond tissue adhesive for worm glueing	Ethicon	Cat#AHV12

**Critical Commercial Assays**		

SuperScript II reverse transcriptase	Invitrogen	Cat#18064-014
KAPA Hifi HotStart kit	KAPA Biosystems	Cat#KK2601
Ampure XP beads	Beckman Coulter	Cat#A63881
Nextera XT DNA sample preparation kit	Illumina	Cat#FC-131-1096

**Deposited Data**		

Genome sequence data of JU1249 and JU2825	This paper	NCBI: PRJNA514933
Genome sequence data of replicate populations for QTL mapping	This paper	NCBI: PRJNA515248
RNA-Seq data of sorted BAG neurons	This paper	GEO: GSE135687

**Experimental Models: Organisms/Strains**		

*C. elegans*: Strain AX1796: *glb-5(Haw) V; npr-1(g320) X*	de Bono lab; Persson et al., 2009	AX1796
*C. elegans*: Strain LSJ1: Bristol strain	*Caenorhabditis* Genetics Center	RRID: WB-STRAIN:LSJ1
*C. angaria*: Strain RGD1: *Caenorhabditis angaria* wild isolate	*Caenorhabditis* Genetics Center	RRID: WB-STRAIN:RGD1
*C. latens*: Strain VX80: *Caenorhabditis latens* wild isolate	*Caenorhabditis* Genetics Center	RRID: WB-STRAIN:VX80
*C. japonica*: Strain DF5081: *Caenorhabditis japonica* wild isolate	*Caenorhabditis* Genetics Center	RRID: WB-STRAIN:DF5081
*C. wallacei*: Strain JU1904: *Caenorhabditis wallacei* wild isolate	*Caenorhabditis* Genetics Center	RRID: WB-STRAIN:JU1904
*C. tropicalis*: Strain JU1373: *Caenorhabditis tropicalis* wild isolate	*Caenorhabditis* Genetics Center	RRID: WB-STRAIN:JU1373
*C. briggsae*: Strain HK105: *Caenorhabditis briggsae* wild isolate	*Caenorhabditis* Genetics Center	RRID: WB-STRAIN:HK105
*C. nigoni*: Strain JU1422: *Caenorhabditis nigoni* wild isolate	*Caenorhabditis* Genetics Center	RRID: WB-STRAIN:JU1422
*C. sinica*: Strain JU800: *Caenorhabditis sinica* wild isolate	*Caenorhabditis* Genetics Center	RRID: WB-STRAIN:JU800
*C. elegans*: Strain ED3011: *C. elegans* wild isolate	*Caenorhabditis* Genetics Center	RRID: WB-STRAIN:ED3011
*C. elegans*: Strain ED3073: *C. elegans* wild isolate	*Caenorhabditis* Genetics Center	RRID: WB-STRAIN:ED3073
*C. elegans*: Strain EG4946: *C. elegans* wild isolate	*Caenorhabditis* Genetics Center	RRID: WB-STRAIN:EG4946
*C. elegans*: Strain JU258: *C. elegans* wild isolate	*Caenorhabditis* Genetics Center	RRID: WB-STRAIN:JU258
*C. elegans*: Strain JU561: *C. elegans* wild isolate	*Caenorhabditis* Genetics Center	RRID: WB-STRAIN:JU561
*C. elegans*: Strain JU1088: *C. elegans* wild isolate	*Caenorhabditis* Genetics Center	RRID: WB-STRAIN:JU1088
*C. elegans*: Strain JU1248: *C. elegans* wild isolate	M.-A. Félix; Troemel et al., 2008	RRID: WB-STRAIN:JU1248
*C. elegans*: Strain JU1543: *C. elegans* wild isolate	M.-A. Félix	RRID: WB-STRAIN:JU1543
*C. elegans*: Strain JU2825: *C. elegans* wild isolate	M.-A. Félix	JU2825
*C. elegans*: Strain MY16: *C. elegans* wild isolate	*Caenorhabditis* Genetics Center	RRID: WB-STRAIN:MY16
*C. elegans*: Strain JU1249: *C. elegans* wild isolate	M.-A. Félix; Zhang et al., 2016	RRID: WB-STRAIN:JU1249
*C. elegans*: Strain AX613: *npr-1(g320) X*	de Bono lab; Persson et al., 2009	AX613
*C. elegans*: Strain JU3221: *arcp-1(mfP22) III; npr-1(215F) X; mfEx94 [arcp-1p::arcp-1b::sl2::gfp; myo-2p::dsRed2]* in JU1249 background	This paper	JU3221
*C. elegans*: Strain AX204: *npr-1(ad609) X*	de Bono lab; Persson et al., 2009	AX204
*C. elegans*: Strain AX6574: *arcp-1(db1082) III; npr-1(ad609) X* 4x outcrossed	This paper	AX6574
*C. elegans*: Strain AX7324: *arcp-1(db1082) III; npr-1(ad609) X* 5x outcrossed	This paper	AX7324
*C. elegans*: Strain AX6723: *arcp-1(db1082) III; npr-1(ad609) X; dbEx975 [arcp-1p::arcp-1b::sl2::gfp; unc-122p::rfp]*	This paper	AX6723
*C. elegans*: Strain AX7094: *arcp-1(db1082) III; npr-1(ad609) X; dbEx1050 [arcp-1p::arcp-1b::sl2::mKate; lin-44p::gfp]*	This paper	AX7094
*C. elegans*: Strain AX6720: *arcp-1(db1082) III; npr-1(ad609) X; dbEx974 [arcp-1p::arcp-1a::sl2::gfp; unc-122p::rfp]*	This paper	AX6720
*C. elegans*: Strain N2: *C. elegans* Bristol strain	*Caenorhabditis* Genetics Center	RRID: WB-STRAIN:N2_(ancestral)
*C. elegans*: Strain MY10: *C. elegans* wild isolate	*Caenorhabditis* Genetics Center	RRID: WB-STRAIN:MY10
*C. elegans*: Strain JU1247: *C. elegans* wild isolate	M.-A. Félix, (Troemel et al., 2008)	RRID: WB-STRAIN:JU1247
*C. elegans*: Strain AX6901: *arcp-1(db1082) III; npr-1(ad609) X; dbEx1002 [fosmid arcp-1p::arcp-1::sl2::gfp; unc-122p::rfp]*	This paper	AX6901
*C. elegans*: Strain AX6766: *arcp-1(db1082) III; npr-1(ad609) X; dbEx984 [gcy-32p::arcp-1b::sl2::gfp; unc-122p::rfp]*	This paper	AX6766
*C. elegans*: Strain AX6805: *arcp-1(db1082) III; npr-1(ad609) X; dbEx990 [flp-17p::arcp-1b::sl2::gfp; unc-122p::rfp]*	This paper	AX6805
*C. elegans*: Strain AX6931: *arcp-1(gk856856) III; npr-1(ad609) X*	This paper	AX6931
*C. elegans*: Strain AX6929: *arcp-1(gk863317) III; npr-1(ad609) X*	This paper	AX6929
*C. elegans*: Strain AX6927: *arcp-1(gk852871) III; npr-1(ad609) X*	This paper	AX6927
*C. elegans*: Strain AX7023: *arcp-1(db1082) III; npr-1(ad609) X; dbEx1035 [gcy-32p::arcp-1b::sl2::mKate; lin-44p::gfp]*	This paper	AX7023
*C. elegans*: Strain AX7095: *arcp-1(db1082) III; npr-1(ad609) X; dbEx990 [flp-17p::arcp-1b::sl2::gfp; unc-122p::rfp]; dbEx1035 [gcy-32p::arcp-1b::sl2::mKate; lin-44p::gfp]*	This paper	AX7095
*C. elegans*: Strain AX7179: *gcy-9(n4470) npr-1(ad609) X*	This paper	AX7179
*C. elegans*: Strain AX7238: *arcp-1(db1082) III; gcy-9(n4470) npr-1(ad609) X*	This paper	AX7238
*C. elegans*: Strain AX7116: *arcp-1(db1082) III; npr-1(ad609) X; dbIs20 [arcp-1p::gfp::arcp-1b; unc-122p::rfp]*	This paper	AX7116
*C. elegans*: Strain AX6969: *malt-1(db1194) II; npr-1(ad609) X; dbIs16 [rab-3p::malt-1::gfp; unc-122p::rfp]*	This paper	AX6969
*C. elegans*: Strain AX7082: *eif-3.L(db1015) II; npr-1(ad609) X; dbIs19 [rab-3p::eif-3.L::gfp; unc-122p::rfp]*	This paper	AX7082
*C. elegans*: Strain AX7419: *npr-1(ad609) X dbEx1075 [flp-17p::pde-1b::gfp; unc-122p::rfp]*	This paper	AX7419
*C. elegans*: Strain AX7422: *arcp-1(db1082) III; npr-1(ad609) X; dbEx1075 [flp-17p::pde-1b::gfp; unc-122p::rfp]*	This paper	AX7422
*C. elegans*: Strain AX2272: *pde-1(ok2924) I; npr-1(ad609) X*	de Bono lab; Couto et al., 2013	AX2272
*C. elegans*: Strain AX7453: *pde-1(ok2924) I; arcp-1(db1082) III; npr-1(ad609) X*	This paper	AX7453
*C. elegans*: Strain AX6881: *npr-1(ad609) X dbEx [flp-17p::YC3.60]*	This paper	AX6881
*C. elegans*: Strain AX6893: *arcp-1(db1082) III; npr-1(ad609) X; dbEx [flp-17p::YC3.60]*	This paper	AX6893
*C. elegans*: Strain AX7842: *arcp-1(db1082) III; npr-1(ad609) X; dbEx [flp-17p::YC3.60]; dbEx1035 [gcy-32p::arcp-1b::sl2::mKate; lin-44::gfp]*	This paper	AX7842
*C. elegans*: Strain AX7845: *arcp-1(db1082) III; npr-1(ad609) X; dbEx [flp-17p::YC3.60] dbEx1172 [gcy-33p::arcp-1b::sl2::mKate; unc-122p::gfp]*	This paper	AX7845
*C. elegans*: Strain AX3516: *npr-1(ad609) X; dbEx614 [gcy-37p::YC2.60; unc-122p::rfp]*	de Bono lab; Fenk and de Bono, 2017	AX3516
*C. elegans*: Strain AX6877: *arcp-1(db1082) III; npr-1(ad609) X; dbEx614 [gcy-37p::YC2.60; unc-122p::rfp]*	This paper	AX6877
*C. elegans*: Strain AX3432: *npr-1(ad609) X; dbEx623 [flp-17p::YC2.60; F15E11.1::mCherry]*	de Bono lab; Gross et al., 2014	AX3432
*C. elegans*: Strain AX7182: *arcp-1(db1082) III; npr-1(ad609) X; dbEx623 [flp-17p::YC2.60; F15E11.1::mCherry]*	This paper	AX7182
*C. elegans*: Strain AX7656: *gcy-33(ok232) V; gcy-31(ok296) npr-1(ad609) X*	This paper	AX7656
*C. elegans*: Strain AX7657: *arcp-1(db1082) III; gcy-33(ok232) V; gcy-31(ok296) npr-1(ad609) X*	This paper	AX7657
*C. elegans*: Strain AX7362: *npr-1(ad609) X; wzIs132 [gcy-9p::gcy-9::dsRed]*	This paper	AX7362
*C. elegans*: Strain AX7361: *arcp-1(db1082) III; npr-1(ad609) X; wzIs132 [gcy-9p::gcy-9::dsRed]*	This paper	AX7361
*C. elegans*: Strain AX7366: *npr-1(ad609) X; wzEx156 [gcy-9p::tax-4::gfp]*	This paper	AX7366
*C. elegans*: Strain AX7365: *arcp-1(db1082) III; npr-1(ad609) X; wzEx156 [gcy-9p::tax-4::gfp]*	This paper	AX7365
*C. elegans*: Strain AX2997: *gcy-33(ok232) V; npr-1(ad609) X; dbEx [flp-17p::gcy-33::gfp; unc-122p::rfp]*	de Bono lab; Gross et al., 2014	AX2997
*C. elegans*: Strain AX7315: *arcp-1(db1082) III; gcy-33(ok232) V; npr-1(ad609) X; dbEx [flp-17p::gcy-33::gfp; unc-122p::rfp]*	This paper	AX7315
*C. elegans*: Strain AX6516: *npr-1(ad609) X; dbEx1053 [gcy-37p::gcy-35::HA::gfp::sl2::mCherry]*	This paper	AX6516
*C. elegans*: Strain AX7278: *arcp-1(db1082) III; npr-1(ad609) X; dbEx1053 [gcy-37p::gcy-35::HA::gfp::sl2::mCherry]*	This paper	AX7278
*C. elegans*: Strain AX7019: *arcp-1(db1082) III; npr-1(ad609) X; dbEx1033 [flp-17p::gfp; unc-122p::rfp]*	This paper	AX7019
*C. elegans*: Strain AX7021: *npr-1(ad609) X; dbEx1033 [flp-17p::gfp; unc-122p::rfp]*	This paper	AX7021
*C. elegans*: Strain AX7268: *npr-1(ad609) X; ynIs34 [flp-19p::gfp]*	This paper	AX7268
*C. elegans*: Strain AX7271: *arcp-1(db1082) III; npr-1(ad609) X; ynIs34 [flp-19p::gfp]*	This paper	AX7271
*C. elegans*: Strain AX7279: *pde-1(ok2924) I; npr-1(ad609) X; ynIs34 [flp-19p::gfp]*	This paper	AX7279
*C. elegans*: Strain AX7272: *arcp-1(db1082) III; npr-1(ad609) X; ynIs34 [flp-19p::gfp]; dbEx1063 [flp-17p::arcp-1b::sl2::mKate; unc-122p::gfp]*	This paper	AX7272
*C. elegans*: Strain AX7273: *arcp-1(db1082) III; npr-1(ad609) X; ynIs34 [flp-19p::gfp] dbEx1035 [gcy-32p::arcp-1b::sl2::mKate; lin-44::gfp]*	This paper	AX7273
*C. elegans*: Strain AX7550: *pde-1(ok2924) I; arcp-1(db1082) III; npr-1(ad609) X; ynIs34 [flp-19p::gfp]*	This paper	AX7550
*C. elegans*: Strain AX7722: *ynIs34 [flp-19p::gfp]* backcrossed 10x in JU2825 background	This paper	AX7722
*C. elegans*: Strain AX7724: *ynIs34 [flp-19p::gfp]* backcrossed 10x in JU1249 background	This paper	AX7724
*C. elegans*: Strain AX7726: *dbEx1063 [flp-17p::arcp-1b::sl2::mKate; unc-122p::gfp]; ynIs34 [flp-19p::gfp]* backcrossed 10x in JU1249 background	This paper	AX7726
*C. elegans*: Strain AX7210: *npr-1(ad609) X; ynIs64 [flp-17p::gfp]*	This paper	AX7210
*C. elegans*: Strain AX7208: *arcp-1(db1082) III; npr-1(ad609) X; ynIs64 [flp-17p::gfp]*	This paper	AX7208
*C. elegans*: Strain AX7321: *flp-19(ok2460) npr-1(ad609) X*	This paper	AX7321
*C. elegans*: Strain AX7322: *arcp-1(db1082) III; flp-19(ok2460) npr-1(ad609) X*	This paper	AX7322
*C. elegans*: Strain AX7754: *arcp-1(db1082) III; npr-1(ad609) X; dbEx1171 [gcy-33p::gfp (sas); unc-122p::rfp]*	This paper	AX7754
*C. elegans*: Strain AX7760: *arcp-1(db1082) III; npr-1(ad609) X; dbEx1173 [gcy-33p::flp-19 (sas); unc-122p::rfp]*	This paper	AX7760
*C. elegans*: Strain AX7788: *arcp-1(db1082) III; npr-1(ad609) X; dbEx1178 [gcy-32p::gfp (sas); unc-122p::rfp]*	This paper	AX7788
*C. elegans*: Strain AX7678: *arcp-1(db1082) III; npr-1(ad609) X; dbEx1153 [gcy-32p::flp-19 (sas); unc-122p::gfp]*	This paper	AX7678
*C. elegans*: Strain AX7793: *npr-1(ad609) X; dbEx1173 [gcy-33p::flp-19 (sas); unc-122p::rfp]*	This paper	AX7793
*C. elegans*: Strain AX7437: *npr-1(ad609) X; dbEx1077 [flp-17p::flp-19::sl2::mKate; unc-122p::gfp]*	This paper	AX7437

**Oligonucleotides**		

Primers used in this study	This paper	Table S2

**Software and Algorithms**		

FlyCapture	Point Grey Research	https://www.flir.com/products/flycapture-sdk
Zentracker	Laurent et al., 2015	https://github.com/wormtracker/zentracker
Neuron Analyzer	Laurent et al., 2015	https://github.com/neuronanalyser/neuronanalyser
RStudio 0.99.903	R Development Core Team, 2015	https://www.R-project.org
Pindel	Ye et al., 2009	http://gmt.genome.wustl.edu/packages/pindel/
Variant Effect Predictor (VEP)	McLaren et al., 2016	www.ensembl.org/info/docs/tools/vep/index.html
Tablet 1.16.09.06	Milne et al., 2013	https://ics.hutton.ac.uk/tablet/
BWA 0.7.8-R455	Li and Durbin, 2009	http://bio-bwa.sourceforge.net/bwa.shtml
Samtools 1.2	Li et al., 2009	http://samtools.sourceforge.net/
Picard 1.114	Broad Institute	http://broadinstitute.github.io/picard/
GATK 3.2-2	Van der Auwera et al., 2013	https://software.broadinstitute.org/gatk/
Bowtie2 0.11.0	Langmead and Salzberg, 2012	http://bowtie-bio.sourceforge.net/bowtie2/index.shtml
rRNA remover code	This paper	https://github.com/lmb-seq/RNA-Seq_utilities
Code for concatenating FASTQ files	This paper	https://github.com/lmb-seq/RNA-Seq_utilities
PRAGUI RNA-Seq analysis pipeline	This paper	https://github.com/lmb-seq/PRAGUI
Mascot	Matrix Science	http://www.matrixscience.com/
Scaffold	Proteome Software Inc	http://www.proteomesoftware.com/products/scaffold/
Prism 7.0	GraphPad Software	https://www.graphpad.com
MATLAB R2014b 8.4	Mathworks	https://www.mathworks.com/products/matlab.html
Metamorph	Molecular Devices	https://www.moleculardevices.com/products/cellular-imaging-systems/acquisition-and-analysis-software/metamorph-microscopy#gref
Fiji (ImageJ)	Schindelin et al., 2012	https://imagej.net/Fiji
Imaris	Bitplane	https://imaris.oxinst.com/

**Other**		

Certified gas mixes	BOC	N/A

### Lead Contact and Materials Availability

Further information and requests for resources and reagents should be directed to and will be fulfilled by the Lead Contact, Mario de Bono (debono@mrc-lmb.cam.ac.uk, mdebono@ist.ac.at).

### Experimental Model and Subject Details

#### Animals

*C. elegans* and other *Caenorhabditis* species were maintained under standard conditions (Stiernagle, 2006) on nematode growth medium (NGM) plates seeded with *E. coli* OP50. Young adult hermaphrodites were used in all experiments. For gonochoristic *Caenorhabditis* species, young adult females were used. For a list of strains and transgene details, see Table S1 and the Key Resources Table.

The mutations in *arcp-1* alleles obtained by forward genetics, and in the JU1249 wild isolate, are shown in Figure S5A. The *C. elegans* strain JU1249 was isolated from a rotten apple collected in 2007 in Santeuil, France (Zhang et al., 2016). A detailed description of the forward genetic screen that isolated the *db1082* allele will be described elsewhere. Causal variants in aggregation-defective mutants from this screen were identified by SNP-based mapping in combination with WGS (Minevich et al., 2012).

#### Microbe strains

The *Escherichia coli* OP50 strain was used as a food source for *C. elegans* and other *Caenorhabditis* species.

### Method Details

#### Molecular biology

Transgenes were cloned using the Multisite Gateway Three-Fragment cloning system (12537-023, Invitrogen) into pDESTR4R3 II. For transgenic lines, the promoter lengths were: *arcp-1p* (1.2 kb for *arcp-1a* and 2 kb for *arcp-1b*), *flp-17p* (3.3 kb), *gcy-32p* (0.6 kb), and *gcy-33p* (1.0 kb). For rescue experiments, cDNA of *arcp-1* isoforms was amplified and cloned into pDONR221, using primers listed in Table S2.

For immunoprecipitation and subcellular localization of ARCP-1, a functional *arcp-1p::gfp::arcp-1b* transgene was made by fusing GFP coding sequences upstream of the *arcp-1b* cDNA sequence. To investigate the subcellular localization of PDE-1 in BAG neurons, the *pde-1b* cDNA sequence was cloned into pDONR221 using primers listed in Table S2. This plasmid was used to generate a *flp-17p::pde-1b::gfp* transgene, by cloning the GFP reporter sequence in frame and downstream of the *pde-1b* cDNA sequence. Details of strains and transgenes used to study the subcellular localization of *gcy-9*, *tax-4*, *gcy-33* and *gcy-35* are provided in Table S1. The *gcy-9p::gcy-9::mCherry* and *gcy-9p::tax-4::gfp* strains were a kind gift from Dr. Niels Ringstad (New York University School of Medicine, USA).

For *flp-19* RNAi, 469 bp of *flp-19* cDNA starting from the sequence GCTTTTCCTGTTAA was cloned in both the sense and antisense orientations. For cell-specific RNAi experiments, we expressed these fragments in BAG using the *gcy-33p* (1.0 kb) and in URX neurons using *gcy-32p* (0.6 kb). To overexpress *flp-19* in BAG, we amplified *flp-19* cDNA using primers listed in Table S2, and fused this sequence to the *flp-17* (3.3 kb) promoter.

To characterize the expression pattern of *arcp-1*, we made a fluorescent reporter transgene by fosmid recombineering. pBALU9 was used to amplify a reporter cassette, containing the *gpd-2* intergenic SL2 sequence and a GFP coding sequence, which was inserted downstream of the *arcp-1* stop codon in the WRM0633bA06 fosmid as described (Tursun et al., 2009). The reporter strain for *flp-19* neuropeptide expression (*flp-19p::gfp*) was a kind gift from Dr. Roger Pocock (Monash University, Australia).

#### Genotyping of natural polymorphisms

Polymorphisms of *npr-1*, *glb-5*, *nath-10* and *arcp-1* genes in *C. elegans* wild isolates were genotyped by PCR. Primers used are listed in Table S2.

#### Behavioral assays

All experiments used young adult hermaphrodite animals, therefore sample stratification was not required within each genotype/condition. For most experiments, measurements were scored using an automated algorithm so blind scoring was not undertaken: see each subsection for details. For details of statistical tests, see the relevant Figure legend for each experiment and also the subsection ‘‘Quantification and Statistical Analysis.’’ All recordings that passed the automated analysis pipeline were included in the final dataset. For rescue and RNAi experiments, behavioral responses and phenotypes were confirmed by testing at least two independent transgenic strains.

*Locomotory responses to CO*_*2*_ *and O*_*2*_

Behavioral responses to gas stimuli were assayed as described (Fenk and de Bono, 2017, Laurent et al., 2015). Animals were acclimated to different O_2_ levels by growing them for one generation at 21% O_2_ (room air) or in a gas-controlled incubator kept at 7% O_2_. For each assay, 20-30 young adult hermaphrodites were transferred onto NGM plates seeded 16–20 h earlier with 20 μL of *E. coli* OP50. To control gas levels experienced by *C. elegans*, animals were placed under a 200 μm deep square polydimethylsiloxane (PDMS) chamber with inlets connected to a PHD 2000 Infusion syringe pump (Harvard apparatus). Humidified gas mixtures were delivered at a flow rate of 3.0 ml/min. Behavioral responses to changes in O_2_ levels were measured by exposing animals to a stimulus train of 7% O_2_ - 21% O_2_ - 7% O_2_ (upshift) or 21% O_2_ - 7% O_2_ - 21% O_2_ (downshift), in which each stimulus comprised a 3 min time interval. Locomotory responses to CO_2_ were measured by exposing animals to a series of 0% CO_2_ (3 min) - X% CO_2_ (3 min) - 0% CO_2_ (3 min), with X corresponding to 1%, 3%, 5% or 10% CO_2_ depending on the experiment. In all CO_2_ assays, a background level of 7% O_2_ was used. Movies were recorded during the stimulus train using FlyCapture (Point Grey Research) on a Leica MZ6 dissecting microscope with a Point Grey Grasshopper camera running at 2 frames/s. Video recording was started 2 min after animals were placed under the PDMS chamber to ensure that the initial environment was in a steady state. In assays where we prolonged the exposure to 7% O_2_ before CO_2_ stimulation, video recording was started 21 min after animals were placed under the PDMS chamber kept at 7% O_2_, and animals were stimulated with 3% CO_2_ at t = 24 min. Videos were analyzed in Zentracker, a custom-written MATLAB software (https://github.com/wormtracker/zentracker). All worms in the field of view were analyzed except those in contact with other animals. Speed was calculated as instantaneous centroid displacement between successive frames. Omega turns were identified as described (Laurent et al., 2015). In total 2-4 assay plates with 20-30 animals per plate were tested per day, and each genotype or condition was assayed in at least two independent experiments. As locomotion measurements were conducted using an automated algorithm, genotypes were not blinded prior to analysis.

*Aggregation and bordering behavior*

L4 animals were picked to a fresh plate 24 h before the assay. Sixty animals were then repicked to the assay plate (an NGM plate seeded 2 days earlier with 100 μL of *E. coli* OP50), and bordering and aggregation was scored 2 and 6 h later. The scorer was blind to genotype. Behavior was always scored on 2-4 assay plates (each containing 60 animals) per day and tested in at least two independent experiments.

*Salt-based associative learning*

Gustatory plasticity was tested as described (Beets et al., 2012, Hukema et al., 2008), in a climate-controlled room set at 20°C and 40% relative humidity. Synchronized young adult hermaphrodites were grown at 25°C on culture plates seeded with *E. coli* OP50. Animals were collected and washed three times over a period of 15 min with chemotaxis buffer (CTX, 5 mM KH_2_PO_4_/K_2_HPO_4_ pH 6.6, 1 mM MgSO_4_, and 1 mM CaCl_2_). Mock-conditioned animals were washed in CTX buffer without NaCl, whereas NaCl-conditioned animals were washed in CTX containing 100 mM NaCl for salt conditioning. Salt chemotaxis behavior of mock- and NaCl-conditioned animals was then tested on four-quadrant plates (Falcon X plate, Becton Dickinson Labware) filled with buffered agar (2% agar, 5 mM KH_2_PO_4_/K_2_HPO_4_ pH 6.6, 1 mM MgSO_4_, and 1 mM CaCl_2_) of which two opposing pairs have been supplemented with 25 mM NaCl. Assay plates were always prepared fresh and left open to solidify and dry for 60 min. Plates were then closed and used on the same day. After the washes, 50 - 150 animals were pipetted on the intersection of the four quadrants and allowed to crawl for 10 min on the quadrant plate. A chemotaxis index was calculated as (n(A) – n(C)) / (n(A) + n(C)) where n(A) is the number of worms within the quadrants containing NaCl and n(C) is the number of worms within the control quadrants without NaCl. The scorer was blind to genotype.

#### Selection-based QTL mapping

*Competition assays*

The *C. elegans* strains JU1249 and JU2825 were competed for several generations using different transfer methods. At the start of the assay, ten JU1249 and JU2825 L4 larvae were put together on a 10 cm NGM plate seeded with *E. coli* OP50. Five biological replicates were maintained at 23°C. Before the cultures starved, a small fraction of the population (200 to 400 animals) was used to seed a fresh culture plate. In Treatment A, the worms were harvested with M9 buffer and 2 μL of worm pellet was transferred to the next plate. In Treatment B, an agar cube (chunk) was cut at the edge of the bacterial lawn and deposited onto the next plate. After each transfer, the remaining animals were stored in M9 buffer at −80°C to quantify the relative proportions of JU1249 and JU2825 alleles.

The genomes of JU1249 and JU2825 were sequenced on an Illumina Hiseq4000 at 20x coverage with paired-end 150 bp reads. For each genome, the raw data were aligned to the reference genome (*C. elegans* WS243 masked from http://wormbase.org) and analyzed using BWA, SAMtools, Picard and Genome Analysis Toolkit (GATK) (Li and Durbin, 2009, Li et al., 2009, Van der Auwera et al., 2013). The accession number for the genomic sequence data of JU1249 and JU2825 is NCBI: PRJNA514933 (https://www.ncbi.nlm.nih.gov/genome/?term=PRJNA514933).

From the output BAM files, homozygous SNPs between the two strains were called and filtered with a raw read depth threshold of 10-300. Allele quantification for the III_663310 polymorphism was performed using pyrosequencing as previously described (Duveau and Félix, 2012). Primers for pyrosequencing are listed in Table S2. In brief, *C. elegans* samples harvested after each transfer were centrifuged at 3,000 rpm for 2 min. Lysates of 2 μL of the worm pellets were used as PCR templates and allele frequencies were quantified with a pyrosequencer (PyroMark Q96 ID; Biotage). The accuracy of this quantification method was estimated by measuring the allele frequencies of PCR products that were amplified using *C. elegans* lysates of known proportions of JU1249 and JU2825 individual L4 larvae. On average, a 2% difference was measured between expected and observed allele frequencies.

*Selection-based QTL mapping experiment*

Segregating populations were generated by crossing the parental JU1249 and JU2825 *C. elegans* wild isolates in both directions, using ten males and two self-sperm exhausted hermaphrodites in each cross. From the F1 progeny, eight biological replicates were set up to generate F2 by crossing again ten males and two self-sperm exhausted hermaphrodites. From each F2 replicate, six males and two L4 stage hermaphrodites were crossed to have plenty of F3 progeny. In the F3 generation, two paired founding populations of 200 L4 larvae (100 from each initial cross direction) were set up per replicate and submitted to contrasted selection regimes. Treatment A transferred worms through liquid harvest and Treatment B by chunking, as described for the competition assay above. In both Treatments, 200-400 animals were transferred before starvation. Males were maintained in the population during each of the first five transfers by picking 50 males. In total 19-20 transfers were done for populations under Treatment A and 17-19 transfers for populations with Treatment B. Genomic DNA of each population (about 10^5^ individuals) was extracted as a pool and sequenced as described above.

The reads of each pool were aligned to the N2 reference genome as described above. The BAM files were filtered for allele information on the positions of homozygous SNPs between the two parents. Allele frequencies were analyzed in each pool. A Cochran-Mantel-Haenszel (CMH) test was used to analyze the consistency of the allele frequency difference between populations with different treatments among the eight replicates, except in the genomic positions 4396879-16406352 on Chromosome IV, where replicate 3 was excluded because one parental genome was fixed in this region in both treatments (McDonald, 2009). The null hypothesis for this CMH test is an equal distribution of sequence reads between the two treatments, and does not consider noise due to allelic drift in the populations, thus inflating the *-log(p value)*. Drift could not be simulated because, for experimental simplicity, population sizes and generations were not controlled during the transfers. We note that, although population size in the experiment was low, the mapping had a relatively good resolution due to the number of populations (16 in total), which yielded independent recombination events.

RStudio (v 0.99.903) and packages (ggplot2, plyr, evobiR) were used for statistical analysis, plots of allele frequencies and CMH tests. Pindel (Ye et al., 2009) was used to detect homozygous indels in the candidate region between JU1249 and JU2825, but no additional polymorphism was found. High quality homozygous variants of the parental strains in the candidate region were annotated using VEP (http://www.ensembl.org//useast.ensembl.org/info/docs/tools/vep/index.html?redirectsrc=//www.ensembl.org%2Finfo%2Fdocs%2Ftools%2Fvep%2Findex.html) (McLaren et al., 2016). The *mfP22* deletion was verified by PCR and Sanger sequencing, using primers listed in Table S2. The accession number for the genomic sequence data of the replicate populations is NCBI: PRJNA515248 (https://www.ncbi.nlm.nih.gov/genome/?term=PRJNA515248).

#### Distribution of *mfP22* allele in wild isolates

To examine the distribution of the *mfP22* deletion in *C. elegans* wild isolates, we monitored the presence of the deletion visually, using Tablet 1.16.09.06 (Milne et al., 2013), for 151 isotypes with whole genome sequences in the CeNDR database (Cook et al., 2016). The *mfP22* allele was only found in JU1249 (Data S2).

#### Confocal microscopy and image analysis

Confocal images were acquired using a Zeiss LSM 710 microscope or a Nikon Eclipse Ti inverted setup coupled to an Andor Ixon EMCCD camera and a spinning disk confocal unit. Projections of z stacks were generated using Fiji (ImageJ).

Expression of *arcp-1* in URX, AQR and PQR was confirmed by co-expression with a *gcy-32p::mCherry* transgene. Expression in BAG and AWB neurons was verified by crossing *arcp-1* reporter strains with *flp-17p::mCherry* and *str-1p::mCherry* marker strains, respectively. Expression in AWC and ASE was confirmed by co-expression with *ceh-36p::RFP* and *odr-1p::RFP* transgenes. For DiI staining, animals were incubated in DiI solution (0.01 mg/ml) for 3 h and washed with M9 buffer before mounting for confocal microscopy.

To quantify the fluorescence of reporter-tagged proteins in cilia and neuron cell bodies, z stack images were taken on a spinning disk confocal microscope using a 60x lens and 100 ms exposure time. Z-projections of image stacks were generated with Fiji (ImageJ). Regions of interest (ROIs) were selected by centering a 50-pixel by 50-pixel square region over the distal dendrite or soma of the BAG neurons, respectively. All measurements were background-corrected by subtracting the mean values of a 50-pixel by 50-pixel square region drawn outside of the neuron.

To quantify the expression of neuropeptide reporters in BAG soma, z stack images were taken on a spinning disk confocal microscope using a 60x lens and 100 ms exposure time. 3D images were reconstituted using the IMARIS software package (Bitplane). GFP pixel intensities brighter than a threshold value (1000 for *flp-19* and 3000 for *flp-17* reporters) were cropped by creating a surface with 0.25 mm details. The mean pixel intensities inside the surface were calculated after background subtraction.

#### Calcium imaging

L4 animals expressing a ratiometric yellow cameleon sensor were picked 24 h before imaging. Animals were glued to agarose pads (2% agarose in M9 buffer, 1 mM CaCl_2_) using Dermabond tissue adhesive (Ethicon) with the nose immersed in a mix of bacterial food (*E. coli* OP50) and M9 buffer. To deliver gas stimuli, glued animals were placed under a microfluidic chamber with inlets connected to a PHD 2000 Infusion syringe pump (Harvard Apparatus) running at a flow rate of 2.5 ml/min. An electronic valve system placed between the syringes and the microfluidic chamber allowed switching between two different gas mixtures in a controlled manner at pre-specified time intervals. Imaging data were analyzed using Neuron Analyzer, a customwritten MATLAB program (code available at https://github.com/neuronanalyser/neuronanalyser). As measurements were conducted using an automated algorithm, genotypes were not blinded prior to analysis.

##### CO_2_-evoked Ca^2+^ activity

Animals expressing a *flp-17p::YC3.60* (yellow cameleon 3.60) transgene were used for ratiometric imaging of relative calcium concentration in BAG cell bodies (Bretscher et al., 2011). After immobilization, animals were placed under a microfluidic PDMS chamber and exposed to a 0% CO_2_ (3 min) - X% CO_2_ (3 min) - 0% CO_2_ (3 min) stimulus train, with X corresponding to 1%, 3% or 5% CO_2_ depending on the experiment. To measure CO_2_-evoked tonic Ca^2+^ activity in BAG, the time interval for CO_2_ stimulation was prolonged from 3 min to 18 min. In all experiments, the background O_2_ level was 7% O_2_. Calcium imaging was done at 2 frames/s on an AZ100 microscope (Nikon) bearing a TwinCam adaptor (Cairn Research) mounted with two ORCAFlash4.0 V2 digital cameras (Hamamatsu) using an AZ Plan Fluor 2x lens with 2x zoom and an exposure time of 500 ms.

##### O_2_-evoked Ca^2+^ activity

We used animals expressing a *gcy-37p::YC2.60* transgene to measure Ca^2+^ activity of URX neurons in response to O_2_ stimuli (Fenk and de Bono, 2017). To measure O_2_ responses in BAG, we used animals expressing a *flp-17p::YC2.60* transgene (Gross et al., 2014). After immobilization, animals were placed under a Y-shaped microfluidic chamber and exposed to an O_2_ upshift (7% - 21% - 7% O_2_) in case of URX imaging, or an O_2_ downshift (21% - 7% - 21% O_2_) for BAG. Each stimulus comprised a 2 min time window. Images were recorded at 2 frames/s with an exposure time of 100 ms for a total of 6 min, on a Zeiss Axiovert inverted microscope with an EMCCD Evolve 512 Deltacamera (Photometrics) and a 40x C-Apochromat lens, using MetaMorph acquisition software (Molecular Devices). To reduce photobleaching an optical density filter 2.0 or 1.5 was used. Excitation light was passed through an excitation filter for CFP (438/24-25, Semrock) and a dichroic filter for YFP (DiO2-25x36, Semrock). A beam splitter (Optical Insights) was used to separate the cyan and yellow emission light using a dichroic filter for 483/32-25 nm (CFP) and 542/27-25 nm (YFP) (Semrock).

#### Immunoprecipitation from *C. elegans*

Two independent coIP experiments were performed to identify putative interactors of ARCP-1B. Samples for GFP-ARCP-1B were always processed in parallel with control samples of other cytoplasmic GFP-tagged proteins (MALT-1-GFP and EIF-3.L-GFP), providing negative controls. For coIP experiments, lysis buffer was prepared with 50 mM HEPES (pH 7.4), 1 mM EGTA, 1 mM MgCl_2_, 100 mM KCl, 10% glycerol, 0.05% Tergitol type-NP40 (Sigma-Aldrich), 1mM DTT, 0.1M PMSF with 1 complete EDTA-free proteinase inhibitor cocktail tablet (Roche Applied Science) per 12 ml. Worms were washed twice in ice-cold M9 and once in ice-cold lysis buffer, and then snap-frozen in liquid nitrogen. Frozen worm pellets (∼10 g) were pulverized using a Freezer/Mill (SPEX SamplePrep). Crude extract was clarified at 4°C for 10 min at 20,000 g, and again for 20 min at 100,000 g with a TLA-100 rotor (Beckman Coulter). For immunoprecipitation, samples were incubated with GFP-Trap (ChromoTek) for 4 h at 4°C, then washed 3 times with 50 mM HEPES, 100 mM KCl. Purified complexes were eluted in SDS-sample buffer at 95°C and further fractionated by SDS-PAGE prior to mass spectrometry analysis.

Proteins were identified by Orbitrap-mass spectrometry and MASCOT database searching. Gel samples were destained with 50% v/v acetonitrile and 50 mM ammonium bicarbonate, reduced with 10 mM DTT, and alkylated with 55 mM iodoacetamide. Digestion was with 6 ng/μl trypsin (Promega) overnight at 37°C, and peptides extracted in 2% v/v formic acid 2% v/v acetonitrile, and analyzed by nano-scale capillary LC-MS/MS (Ultimate U3000 HPLC, Thermo Scientific Dionex) at a flow of ∼300 nL/min. A C18 Acclaim PepMap100 5 μm, 100 μm x 20 mm nanoViper (Thermo Scientific Dionex), trapped the peptides prior to separation on a C18 Acclaim PepMap100 3 μm, 75 μm x 250 mm nanoViper. Peptides were eluted with an acetonitrile gradient. The analytical column outlet was interfaced via a nano-flow electrospray ionisation source with a linear ion trap mass spectrometer (Orbitrap Velos, Thermo Scientific). Data dependent analysis was performed using a resolution of 30,000 for the full MS spectrum, followed by ten MS/MS spectra in the linear ion trap. MS spectra were collected over a m/z range of 300–2000. MS/MS scans were collected using a threshold energy of 35 for collision-induced dissociation. LC-MS/MS data were searched against the UniProt KB database using Mascot (Matrix Science), with a precursor tolerance of 10 ppm and a fragment ion mass tolerance of 0.8 Da. Two missed enzyme cleavages and variable modifications for oxidised methionine, carbamidomethyl cysteine, pyroglutamic acid, phosphorylated serine, threonine and tyrosine were included. MS/MS data were validated using the Scaffold program (Proteome Software Inc).

#### RNA-seq of sorted BAG neurons

##### Adult cell isolation

Synchronized young adult hermaphrodites with GFP-labeled BAG neurons (expressing a *flp-17p::gfp* transgene) were acutely dissociated as described (Kaletsky et al., 2016). Synchronized adult worms were washed with M9 buffer to remove excess bacteria. The pellet (∼250 μl) was washed with 500 μl lysis buffer (200 mM DTT, 0.25% SDS, 20 mM HEPES pH 8.0, 3% sucrose) and resuspended in 750 μl lysis buffer. Worms were incubated in lysis buffer for 6.5 min at room temperature. The pellet was washed 5 times with M9 and resuspended in 20 mg/ml pronase from *Streptomyces griseus* (Roche). Worms were pipetted up and down for 12 min at room temperature; then ice-cold PBS buffer containing 2% fetal bovine serum (GIBCO) was added. Cell suspensions were passed over a 5 μm syringe filter (Millipore). The filtered cells were diluted in PBS and sorted using a Sony Biotechnology Synergy High Speed Cell Sorter. Gates for detection were set by comparison to *npr-1* cell suspensions prepared on the same day alongside the experimental samples. Positive fluorescent events were sorted directly into Eppendorf tubes containing 10 μL of 0.2% (vol/vol) Triton X-100 and 2 U μl^-1^ RNase inhibitor. Six biological replicates were prepared for each genotype, i.e., *npr-1(ad609)* and *arcp-1(db1082); npr-1(ad609)* animals. For each replicate sample, approximately 4,000 GFP positive events were collected.

##### RNA amplification and library preparation

RNA-seq was done using a Smart-seq2 protocol as described (Picelli et al., 2014). After neuron isolation by FACS, cDNA was prepared from each sample by reverse transcription using SuperScript II reverse transcriptase (18064-014, Invitrogen), Oligo-dT_30_ and Template-Switching Oligonucleotide (TSO) primers listed in Table S2. After the first strand reaction, the cDNA was amplified with the KAPA Hifi HotStart kit (KK2601, KAPA Biosystems) and IS PCR primers listed in Table S2. cDNA was then purified using Ampure XP beads (A 63881, Beckman Coulter), tagmented and 1 μg was used for preparing libraries with the Illumina Nextera XT DNA sample preparation kit (FC-131-1096, Illumina), as per manufacturer suggested practices. Sequencing libraries were then submitted for sequencing on the Illumina HiSeq 4000 platform.

##### RNA-seq data analysis

Prior to analysis the raw files were pre-processed using Bowtie2 version 0.11.0 to remove ribosomal RNA that mapped to a ribosomal RNA sequence library (Wormbase, WS255). Additionally, FASTQ files relating to the same sample but sequenced over multiple flow cell lanes were concatenated to give a single file. Custom rRNA_remover and rna_seq_lane_merger scripts were used (available on GitHub: https://github.com/lmb-seq/RNA-Seq_utilities). The files were then processed by PRAGUI - a Python3 pipeline for RNA-seq data analysis. PRAGUI automates analysis by incorporating widely used RNA-seq processing packages including: Trim Galore, FastQC, STAR, DESeq2, HTSeq, Cufflinks and MultiQC. PRAGUI can be found at: https://github.com/lmb-seq/PRAGUI. The following parameters were used with PRAGUI: DESeq2 analysis (labeled as “DESeq”), unstranded paired-end library, worm organism with *C. elegans* genome fasta file and canonical gene set gtf file (Wormbase, WS255), STAR arguments set to “–outSAMstrandFieldDESeq intronMotif–readFilesCommand zcat -c–outSAMtype BAM SortedByCoordinate,” mapq set to 20. All other PRAGUI parameters were kept default. 5.5 − 17 million reads were obtained per sample and mapped to the *C. elegans* genome. Sequences are deposited at GEO (GSE135687).

### Quantification and Statistical Analysis

The number of animals and replicates used per experiment is described in detail in the ‘‘Methods Details’’ subsection for each assay and in the relevant Figure legends. Specifically, for the main behavioral assays: locomotory responses to CO_2_ and O_2_ were measured in >4 trials per condition with 20-30 animals each; aggregation and bordering assays were conducted with >4 trials per genotype of 50 - 60 animals each.

Statistical analyses used GraphPad Prism 7.0 and Mathworks MATLAB R2014b (8.4). Exact tests used are indicated in figure legends. In general, where more than two groups tested with a single condition were compared, a one-way ANOVA with Tukey’s or Šidák’s multiple comparisons test was used. Where multiple groups tested with multiple conditions were compared, a two-way ANOVA with Tukey’s or Šidák’s post hoc test was used. Where appropriate, a D’Agostino & Pearson or Shapiro-Wilk normality test was conducted to assess if the data fit a normal distribution. For locomotory assays where two groups were compared over one time interval, we chose time intervals where we expected the locomotory changes to have plateaued and used a Mann-Whitney *u* test for statistical comparisons as described (Laurent et al., 2015). For the intervals of interest, we determined independent per-subject means derived from individuals flagged as continuously valid for at least 10 s during the interval. We considered all individuals in the field of view as valid except those in contact with other animals and those that were off the food lawn or less than half a body-length from the border. Following these criteria, each individual was sampled at most once per interval.

### Data and Software Availability

#### Datasets

The genome sequencing data of JU1249 and JU2825 is available on NCBI: PRJNA514933 (https://www.ncbi.nlm.nih.gov/genome/?term=PRJNA514933). The genomic sequence data of the replicate populations for QTL mapping is available on NCBI: PRJNA515248 (https://www.ncbi.nlm.nih.gov/genome/?term=PRJNA515248). Sequence data from the RNA-Seq analysis of sorted BAG neurons is deposited on GEO: GSE135687 (https://www.ncbi.nlm.nih.gov/geo/query/acc.cgi?acc=GSE135687).

#### Codes

##### Locomotory assays

Videos of locomotory assays were analyzed in Zentracker, a custom-written MATLAB software available on https://github.com/wormtracker/zentracker.

##### Calcium imaging

Recordings were analyzed using Neuron Analyzer, a customwritten MATLAB program available at https://github.com/neuronanalyser/neuronanalyser.

##### RNA-seq analysis

Codes for removing rRNA sequences from datasets and for concatenating FASTQ files relating to the same sample but sequenced over multiple flow cell lanes, are available on GitHub: https://github.com/lmb-seq/RNA-Seq_utilities. The git repository for PRAGUI can be found at: https://github.com/lmb-seq/PRAGUI.

## Supporting Citations

The following references appear in the Supplemental Information: de Bono and Bargmann (1998); Félix and Duveau, 2012, Kiontke et al., 2011; Richaud et al. (2018).

## Supplemental Information

Document S1. Figures S1–S7 and Tables S1 and S2

Data S1. Data Used for Selection-Based QTL Mapping, Related to Figure 2 A) List of single-nucleotide polymorphisms (SNPs) between JU1249 and JU2825. B) Number of reads in the eight replicates of treatment A, by SNP. C) Number of reads in the eight replicates of treatment B, by SNP. D) Allele frequencies in the eight replicates of treatment A, by SNP. Data in red were used to generate Figure S4C. E) Allele frequencies in the eight replicates of treatment B, by SNP. Data in red were used to generate Figure S4C. F) Allele frequencies in the eight replicates of treatment A, displayed using a sliding window 10 SNPs wide and a step size of one SNP for all chromosomes except chromosome IV, where we used a sliding window 100 SNPs wide and a step size of one SNP. G) Allele frequencies in the eight replicates of treatment B, displayed using a sliding window 10 SNPs wide and a step size of one SNP for all chromosomes except chromosome IV, where we used a sliding window 100 SNPs wide and a step size of one SNP. H) Differences in allele frequencies between treatment A and treatment B in each of the eight replicates, displayed using a sliding window 5 SNPs wide and a step size of one SNP for all chromosomes, except a sliding window 100 SNPs wide and a step size of one SNP for chromosome IV. In each pair of replicates, the same genomic positions were first selected between treatment A and treatment B, before subtracting allele frequencies between treatments. I) Read statistics used for the CMH analysis. The same genomic positions were first selected for both treatments among all the replicates, before the CMH analysis. The part in gray on chromosome IV for replicate 3 was not used in the test as one parental allele was fixed in both treatments. J) Annotation of variants detected in JU1249 compared to the reference N2, using the VEP algorithm. The F34D10.6 deletion (in red) appears as the only high impact variation. K) Annotation of variants detected in JU2825 compared to the reference N2.

Data S2. Distribution of the *mfP22* Deletion, Related to Figure 2 List of wild isolates where the *mfP22* deletion is absent, based on mapped sequence reads available at the *Caenorhabditis elegans* Natural Diversity Resource, http://elegansvariation.org (Cook et al., 2016).

Data S3. Protein Identifications from CoIP of GFP-ARCP-1B, Related to Figure 5 Proteins identified by mass spectrometry in two independent coIP experiments for interactors of GFP-ARCP-1B. IP of GFP-tagged cytoplasmic proteins (MALT-1-GFP and EIF-3.L-GFP) provided a negative control. Total spectrum counts in GFP-ARCP-1B and control samples are listed for proteins that were at least 3-fold enriched in the GFP-ARCP-1B sample in both experiments.

Data S4. Expression Profiling of BAG Neurons in *npr-1* and *arcp-1; npr-1* Animals Using RNA-Seq, Related to Figure 6 A-D) Genes expressed in BAG neurons, which were isolated by FACS from adult *npr-1* and *arcp-1; npr-1* animals, with six biological replicates per genotype. A-B) Values indicate transcripts per kilobase million (TPM). C-D) Values show fragments per kilobase million (FPKM). Genes are listed based upon an expression detection threshold of 1 count per million reads per gene in at least 6 samples. E) Genes differentially expressed in BAG neurons of *npr-1* and *arcp-1; npr-1* animals.

Document S2. Article plus Supplemental Information
