# Supplementary material for: Natural Variation in a Dendritic Scaffold Protein Remodels Experience-Dependent Plasticity by Altering Neuropeptide Expression
Source: Neuron. 2020 Jan 8;105(1):106–121.e10. doi: 10.1016/j.neuron.2019.10.001 (PMC6953435; doi:10.1016/j.neuron.2019.10.001)
Supplement: Document S1. Figures S1–S7 and Tables S1 and S2 [file mmc1.pdf]

Neuron, Volume *105*

Supplemental Information

Natural Variation in a Dendritic Scaffold Protein Remodels Experience-Dependent Plasticity by Altering Neuropeptide Expression

Isabel Beets, Gaotian Zhang, Lorenz A. Fenk, Changchun Chen, Geoffrey M. Nelson, Marie-Anne Félix, and Mario de Bono

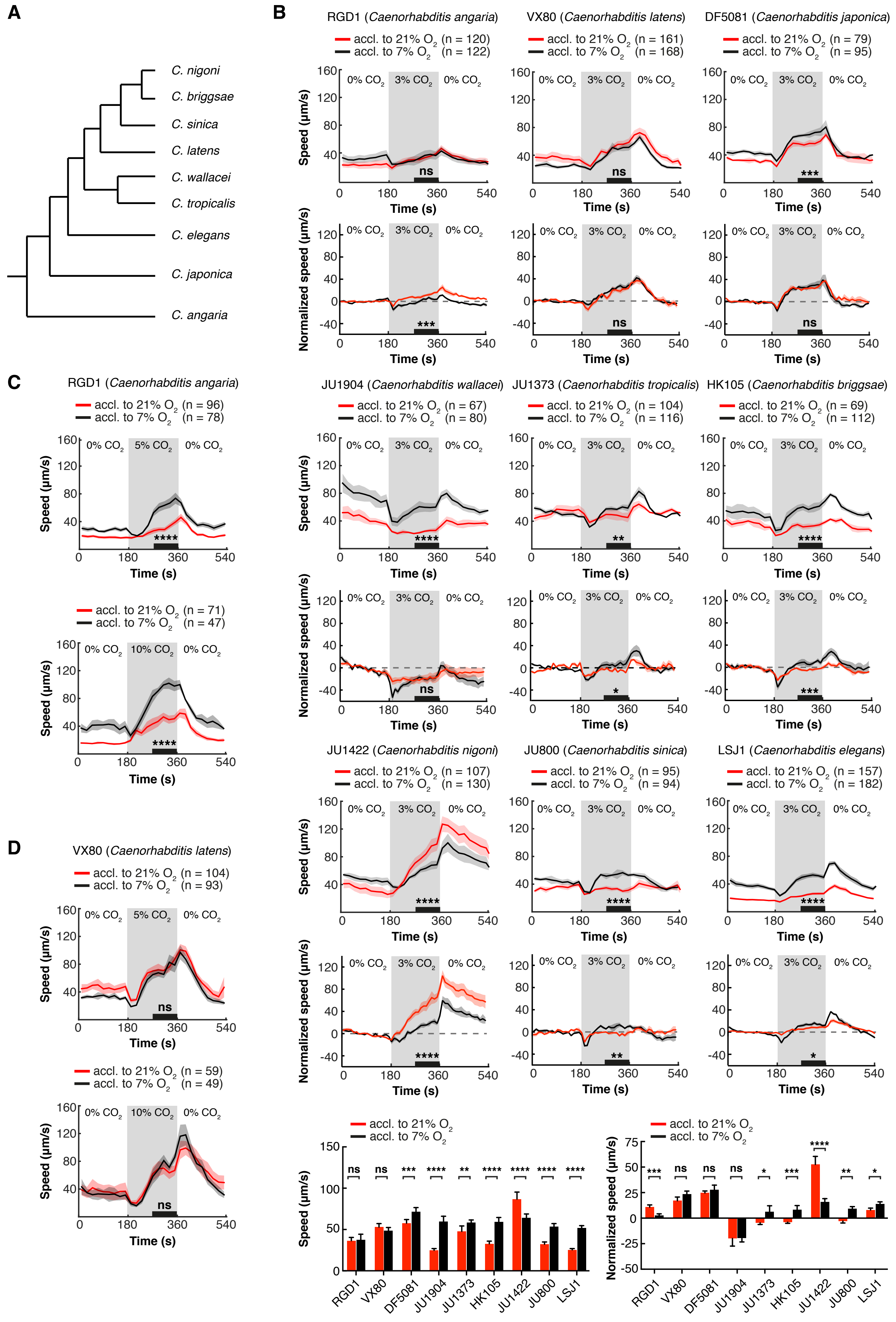
A
C. nigoni
C. briggsae
C. sinica
C. latens
C. wallacei
C. tropicalis
C. elegans
C. japonica
C. angaria
B
RGD1 (Caenorhabditis angaria)
VX80 (Caenorhabditis latens)
DF5081 (Caenorhabditis japonica)
JU1904 (Caenorhabditis wallacei)
JU1373 (Caenorhabditis tropicalis)
HK105 (Caenorhabditis briggsae)
JU1422 (Caenorhabditis nigoni)
JU800 (Caenorhabditis sinica)
LSJ1 (Caenorhabditis elegans)
accl. to 21% O2
accl. to 7% O2
0% CO2
3% CO2
Speed (µm/s)
Normalized speed (µm/s)
Time (s)
C
RGD1 (Caenorhabditis angaria)
5% CO2
10% CO2
D
VX80 (Caenorhabditis latens)

Figure S1, related to Figure 1. The effect of recent O_2 experience on CO_2 escape behavior varies across *Caenorhabditis* species. A) Cladogram of *Caenorhabditis* species studied, based on (Kiontke et al., 2011). **B)** The influence of previous O_2 experience on CO_2 escape behavior varies between *Caenorhabditis* species. In most species, including *C. elegans*, acclimation to 7% O_2 increases locomotory arousal at 3% CO_2. In *C. nigoni*, this effect is reversed and acclimation to 7% O_2 reduces CO_2-evoked locomotory arousal. In some species, such as *C. latens*, O_2 memory does not alter the effect of CO_2 on locomotory activity. For each strain, top panels show speed in response to a 0% - 3% - 0% CO_2 stimulus train. Bottom panels depict speed normalized by subtracting baseline activity before CO_2 stimulation. **C)** In *C. angaria*, the effect of O_2 memory on CO_2 escape becomes apparent at higher CO_2 concentrations. Animals acclimated to 7% O_2 are more strongly aroused by 5% and 10% CO_2 than animals acclimated to 21% O_2. **D)** O_2 memory does not influence CO_2 responses of *C. latens* at any CO_2 concentration tested.

For B-D, solid lines indicate mean speed and shaded areas show SEM. Black bars indicate the time interval used for statistical comparisons. Bar graphs plot mean speed ± SEM for these time intervals. n = number of animals tested in at least 4 trials for each condition. Mann-Whitney *u* test; * $P < 0.05$; ** $P < 0.01$; *** $P < 0.001$; **** $P < 0.0001$; ns, not significant.

Figure S2: Related to Figure 1 Beets et al.

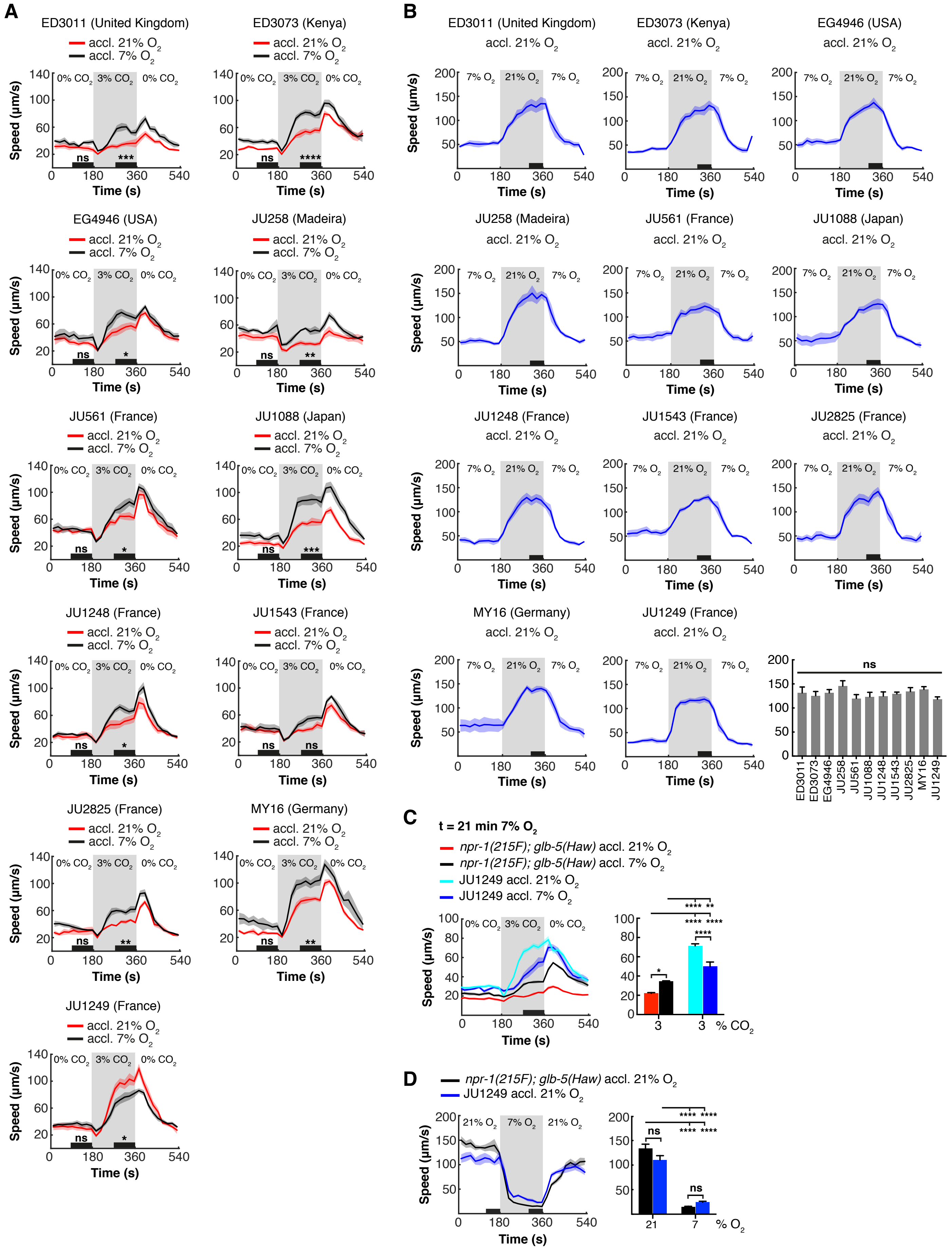

Figure S2, related to Figure 1. ***C. elegans*** **wild isolates show natural variation in the experience-dependent plasticity of CO_2 responses. A)** How O_2 experience modulates the behavioral response to CO_2 varies across *C. elegans* wild isolates. In most isolates acclimation to 7% O_2 enhances locomotory arousal in response to CO_2. By contrast, for JU1249 acclimation to 7% O_2 suppresses locomotory arousal at 3% CO_2. Black bars indicate time intervals used for statistical comparisons here and in Figure 1C. Two-way ANOVA with Šidák test; n = 6 assays. **B)** *C. elegans* wild isolates display similar responses to 21% O_2. One-way ANOVA with post-hoc Tukey's test; n = 6 assays. **C)** JU1249 animals show altered plasticity of CO_2 responses after prolonged exposure to 7% O_2 before CO_2 stimulation. Animals acclimated to 21% or 7% O_2 were exposed to 7% O_2 in the assay chamber for 24 min before being challenged with 3% CO_2. Two-way ANOVA with post-hoc Tukey's test; n = 6 assays. **D)** JU1249 animals resemble controls in their behavioral responses to an O_2 downshift from 21% O_2 to 7% O_2. Two-way ANOVA with post-hoc Tukey's test; n = 6 assays.

For A-D, 20-30 animals were tested in 6 assays per condition. Solid lines depict the mean speed and shaded areas show the SEM. Black bars indicate time intervals used for statistical comparisons. Bar graphs plot mean speed ± SEM for these time intervals. * $P < 0.05$; ** $P < 0.01$; *** $P < 0.001$; **** $P < 0.0001$; ns, not significant.

A

4768905 ***npr-1*** **(chromosome X)** 4768816

```
N2      TACTTTTGCACTGAAAAGTGGGAATCTGCCAAGTCTAGAAGAGCCTACACAATGATCGTGATGCTCGCCCAATTCGTGGTCCCATTCGCT
        -Y--F--C--T--E--K--W--E--S--A--K--S--R--R--A--Y--T--M--I--V--M--L--A--Q--F--V--V--P--F--A-

JU1249  TACTTTTGCACTGAAAAGTGGGAATCTGCCAAGTCTAGAAGAGCCTACACAATGATCGTGATGCTCGCCCAATTCGTGGTCCCATTCGCT
        -Y--F--C--T--E--K--W--E--S--A--K--S--R--R--A--Y--T--M--I--V--M--L--A--Q--F--V--V--P--F--A-

JU2825  TACTTTTGCACTGAAAAGTGGGAATCTGCCAAGTCTAGAAGAGCCTACACAATGATCGTGATGCTCGCCCAATTCGTGGTCCCATTCGCT
        -Y--F--C--T--E--K--W--E--S--A--K--S--R--R--A--Y--T--M--I--V--M--L--A--Q--F--V--V--P--F--A-
```

4768815 4768759

```
N2      GTCATGGCCTTCTGCTATGCAAATATTGTTTCGGTGCTCAGCAAGCGTGCTCAGACA
        -V--M--A--F--C--Y--A--N--I--V--S--V--L--S--K--R--A--Q--T-

JU1249  GTCATGGCCTTCTGCTATGCAAATATTTTTTCGGTGCTCAGCAAGCGTGCTCAGACA
        -V--M--A--F--C--Y--A--N--I--F--S--V--L--S--K--R--A--Q--T-

JU2825  GTCATGGCCTTCTGCTATGCAAATATTTTTTCGGTGCTCAGCAAGCGTGCTCAGACA
        -V--M--A--F--C--Y--A--N--I--F--S--V--L--S--K--R--A--Q--T-
```

B

5351237 ***nath-10*** **(chromosome I)** 5351148

```
N2      CAGAATTCGAACGACATTACCGGAGAGCATACTTGTATTATACTGAAAGGACTCGAACACGGAGGATCTGATAGTGATGGTAAGTTAAAT
        -Q--N--S--N--D--I--T--G--E--H--T--C--I--I--L--K--G--L--E--H--G--G--S--D--S--D--E--E--P--S-

JU1249  CAGAATTCGAACGACATTACCGGAGAGCATACTTGTATTATGCTGAAAGGACTCGAACACGGAGGATCTGATAGTGATGGTAAGTTAAAT
        -Q--N--S--N--D--I--T--G--E--H--T--C--I--M--L--K--G--L--E--H--G--G--S--D--S--D--E--E--P--S-

JU2825  CAGAATTCGAACGACATTACCGGAGAGCATACTTGTATTATGCTGAAAGGACTCGAACACGGAGGATCTGATAGTGATGGTAAGTTAAAT
        -Q--N--S--N--D--I--T--G--E--H--T--C--I--M--L--K--G--L--E--H--G--G--S--D--S--D--E--E--P--S-
```

5351147 5351058

```
N2      TTGTTTTTGAATTTGAGTTTAAATTTTCGAGGAGTTTTTCTAAAGTAAATTCGTAAATTTAATTTCAAATTTCAGAAGAACCATCTGCCA
        -A--T--W--L--P--V--Y--W--R--E--F--R--R--R--I--V--N--L--L--S--F--D--F--S--S--F--P--A--Q--M-

JU1249  TTGTTTTTGAATTTGAGTTTAAATTTTCGAGGAGTTTTTCTAAAGTAAATTCGTAAATTTAATTTCAAATTTCAGAAGAACCATCTGCCA
        -A--T--W--L--P--V--Y--W--R--E--F--R--R--R--I--V--N--L--L--S--F--D--F--S--S--F--P--A--Q--M-

JU2825  TTGTTTTTGAATTTGAGTTTAAATTTTCGAGGAGTTTTTCTAAAGTAAATTCGTAAATTTAATTTCAAATTTCAGAAGAACCATCTGCCA
        -A--T--W--L--P--V--Y--W--R--E--F--R--R--R--I--V--N--L--L--S--F--D--F--S--S--F--P--A--Q--M-
```

C

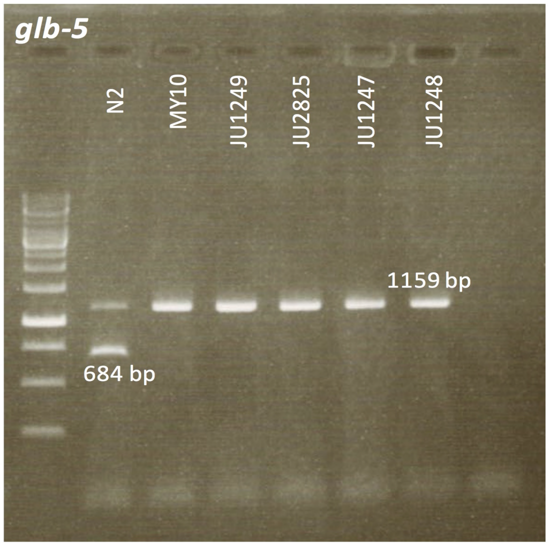

Figure S3, related to Figure 2. The wild isolate JU1249 genetically differs from the non-aggregating N2 laboratory strain. A) Partial sequence of the *npr-1* locus in the *C. elegans* N2 laboratory strain and in wild isolates JU1249 and JU2825. Unlike N2, JU1249 has the wild allele of *npr-1* (de Bono and Bargmann, 1998). **B)** JU1249 also carries the wild (non-N2) allele of the *nath-10* locus (Félix and Duveau, 2012). **C)** Gel electrophoresis of a PCR product for the *glb-5* polymorphism, as identified in (McGrath et al., 2009; Persson et al., 2009). JU1249 contains the wild *glb-5* allele, similar to other wild isolates (MY10, JU2825, JU1247 and JU1248).

Figure S4: Related to Figure 2

Beets et al.

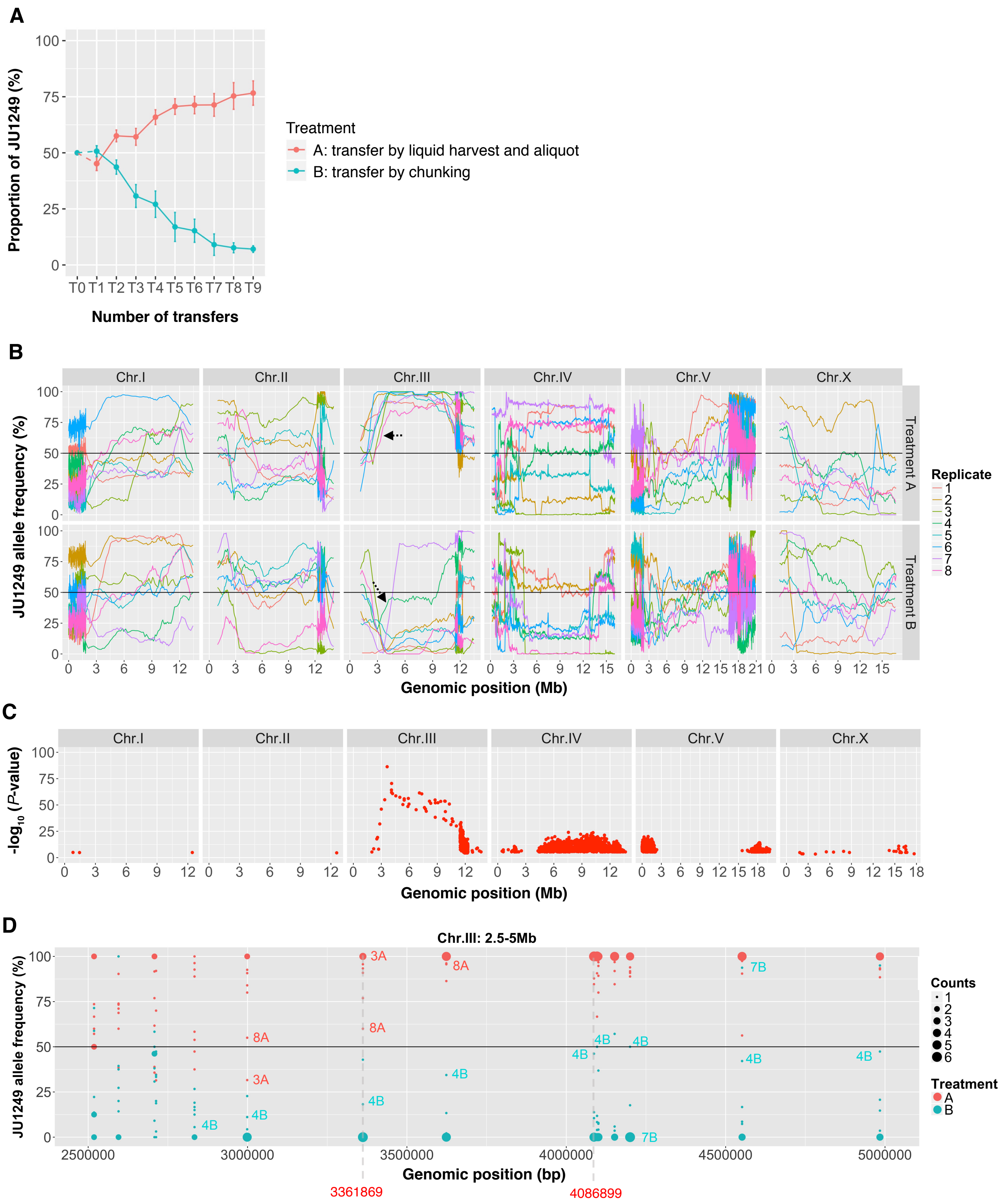

Figure S4, related to Figure 2. JU1249 allele frequency of JU1249 under different selection regimes for aggregating or solitary behavior. A) Competition assay between JU1249 and JU2825. Using SNP pyrosequencing, the proportion of JU1249 animals was measured at each transfer over the course of nine transfers. JU1249 outcompeted JU2825 in the populations transferred by Treatment A (liquid harvest and aliquot). In populations transferred by Treatment B (chunking at the lawn border where aggregating animals accumulate), JU2825 outcompeted JU1249. Plot shows the mean proportion of JU1249 animals ± SEM for five replicates of the competition assay. **B)** Average frequency of JU1249 alleles for eight replicate populations transferred by Treatment A (top panel) and Treatment B (bottom panel). For each chromosome, we used a sliding window with a width of 10 SNPs and a step size of one SNP, except for the more divergent chromosome IV (sliding window of 100-SNP). Replicates are shown in different colors. Arrows indicate recombinant selection events for replicates 8A and 4B. **C)** Cochran-Mantel-Haenszel (CMH) test for analysis of the consistency of allele frequency differences between populations among the eight population pairs. Replicate 3 was excluded for genomic positions 4396879-16406352 on Chromosome IV (see STAR Methods). Plots show significant *P*-values as $-\log_{10}$ (*P*-value) adjusted using a Bonferroni correction. **D)** JU1249 allele frequencies in the eight replicates transferred by Treatment A (red) and B (green), for the region 2,500,000-5,000,000 bp on chromosome III. Dot size represents the number of replicates at a given frequency. The candidate region of 3,361,869-4,086,899 bp is defined as the region where all replicates show a high JU1249 allele frequency in Treatment A and a low JU1249 frequency in Treatment B. The most relevant replicates for defining the interval associated with the aggregation phenotype are indicated next to the corresponding data points. The data set used in D is indicated in red in Data Table S1D and S1E.

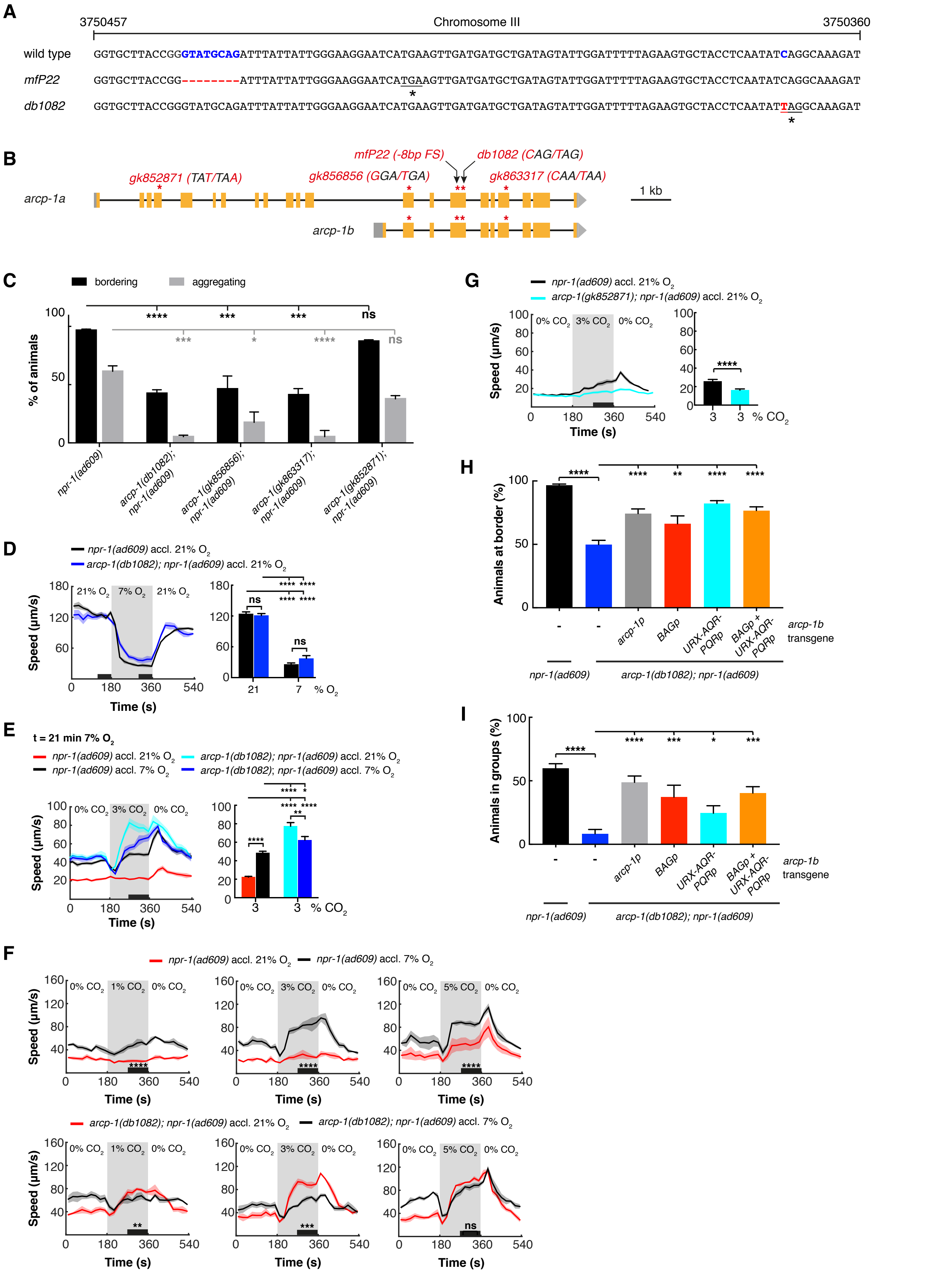
A
3750457
Chromosome III
3750360
wild type
GGTGCTTACCGGGTATGCAGATTTATTATTGGGAAGGAATCATGAAGTTGATGATGCTGATAGTATTGGATTTTTAGAAGTGCTACCTCAATATCAGGCAAAGAT
mfP22
GGTGCTTACCGG--------ATTTATTATTGGGAAGGAATCATGAAGTTGATGATGCTGATAGTATTGGATTTTTAGAAGTGCTACCTCAATATCAGGCAAAGAT
db1082
GGTGCTTACCGGGTATGCAGATTTATTATTGGGAAGGAATCATGAAGTTGATGATGCTGATAGTATTGGATTTTTAGAAGTGCTACCTCAATATTAGGCAAAGAT
B
mfP22 (-8bp FS)
db1082 (CAG/TAG)
gk852871 (TAT/TAA)
gk856856 (GGA/TGA)
gk863317 (CAA/TAA)
arcp-1a
arcp-1b
1 kb
C
bordering
aggregating
% of animals
npr-1(ad609)
arcp-1(db1082); npr-1(ad609)
arcp-1(gk856856); npr-1(ad609)
arcp-1(gk863317); npr-1(ad609)
arcp-1(gk852871); npr-1(ad609)
D
npr-1(ad609) accl. 21% O2
arcp-1(db1082); npr-1(ad609) accl. 21% O2
21% O2
7% O2
21% O2
Speed (μm/s)
Time (s)
% O2
E
t = 21 min 7% O2
npr-1(ad609) accl. 21% O2
npr-1(ad609) accl. 7% O2
arcp-1(db1082); npr-1(ad609) accl. 21% O2
arcp-1(db1082); npr-1(ad609) accl. 7% O2
0% CO2
3% CO2
0% CO2
Speed (μm/s)
Time (s)
% CO2
F
npr-1(ad609) accl. 21% O2
npr-1(ad609) accl. 7% O2
1% CO2
3% CO2
5% CO2
arcp-1(db1082); npr-1(ad609) accl. 21% O2
arcp-1(db1082); npr-1(ad609) accl. 7% O2
Speed (μm/s)
Time (s)
G
npr-1(ad609) accl. 21% O2
arcp-1(gk852871); npr-1(ad609) accl. 21% O2
Speed (μm/s)
Time (s)
% CO2
H
Animals at border (%)
arcp-1p
BAGp
URX-AQR-PQRp
BAGp + URX-AQR-PQRp
arcp-1b transgene
npr-1(ad609)
arcp-1(db1082); npr-1(ad609)
I
Animals in groups (%)
arcp-1p
BAGp
URX-AQR-PQRp
BAGp + URX-AQR-PQRp
arcp-1b transgene
npr-1(ad609)
arcp-1(db1082); npr-1(ad609)

Figure S5, related to Figures 2 and 3. The ARCP-1B protein, but not ARCP-1A, promotes aggregation behavior and suppresses locomotory arousal by CO_2. A) Partial sequence of the *arcp-1* gene on chromosome III indicating the 8bp deletion (*mfP22*) in JU1249 and the C/T mutation of the *db1082* allele. Asterisks mark a premature stop codon. **B)** Structure of *arcp-1a* and *arcp-1b* transcripts with the positions of mutations used in this study marked by asterisks. The mutant *gk852871* allele affects only *arcp-1a*, whereas the other alleles affect both transcripts of *arcp-1*. Orange boxes represent exons; 5' and 3' UTRs are shown in gray; lines depict introns. **C)** Bordering and aggregation behaviors of the different *arcp-1* mutants. Animals with a mutation that affects only *arcp-1a* show normal bordering and aggregation, whereas mutants affected in both *arcp-1* isoforms have defects in these behaviors. Bars plot mean ± SEM. For each assay, 50-60 animals were transferred to a bacterial lawn and behaviors were scored after 2 hours. $n \geq 4$ assays for all genotypes. Kruskal-Wallis test with Dunn's correction. **D)** *arcp-1* mutants display normal behavioral responses to an O_2 downshift. Two-way ANOVA with Tukey test; n = 6 assays. **E)** *arcp-1* animals show altered plasticity of CO_2 responses when stimulated with CO_2 many minutes after transfer to 7% O_2. Animals acclimated to 21% or 7% O_2 were exposed to 7% O_2 in the assay chamber for 24 min before being challenged with 3% CO_2. Two-way ANOVA with Tukey test; n = 6 - 7 assays. **F)** Experience-dependent plasticity of CO_2 responses in *arcp-1* mutants is affected at different CO_2 concentrations. In *arcp-1* mutants, unlike *npr-1* animals, acclimation to 21% O_2 fails to suppress locomotory arousal at 1%, 3% or 5% CO_2. n = 53 - 67 animals for *npr-1*, n = 62 - 72 animals for *arcp-1; npr-1*. Mann-Whitney *u* test. **G)** Animals defective only in *arcp-1a* do not show the CO_2 phenotype observed in JU1249 and *db1082* animals. n = 97 animals for *npr-1* and n = 98 animals for *arcp-1; npr-1*. Mann-Whitney *u* test. **H-I)** The bordering and aggregation phenotypes of *arcp-1; npr-1* animals are rescued by expressing *arcp-1b* from its own promoter (*arcp-1p*), by BAG-specific expression using the *flp-17* promoter (*BAGp*), and by cell-specific expression in URX, AQR and PQR from the *gcy-32* promoter (*URX-AQR-PQRp*). Bars plot mean ± SEM. For each assay, 50-60 animals were transferred to a bacterial lawn and behaviors were scored after 6 hours. $n \geq 8$ assays. One-way ANOVA with Holm-Šidák's test. For D-G, 20-30 animals per assay were tested in at least 4 trials for each genotype. Left panel shows mean speed traces (solid lines) ± SEM (shaded areas). Black bars indicate the time intervals used for statistical comparisons. Bar graphs plot mean speed ± SEM for these intervals. * $P < 0.05$; ** $P < 0.01$; *** $P < 0.001$; **** $P < 0.0001$; ns, not significant.

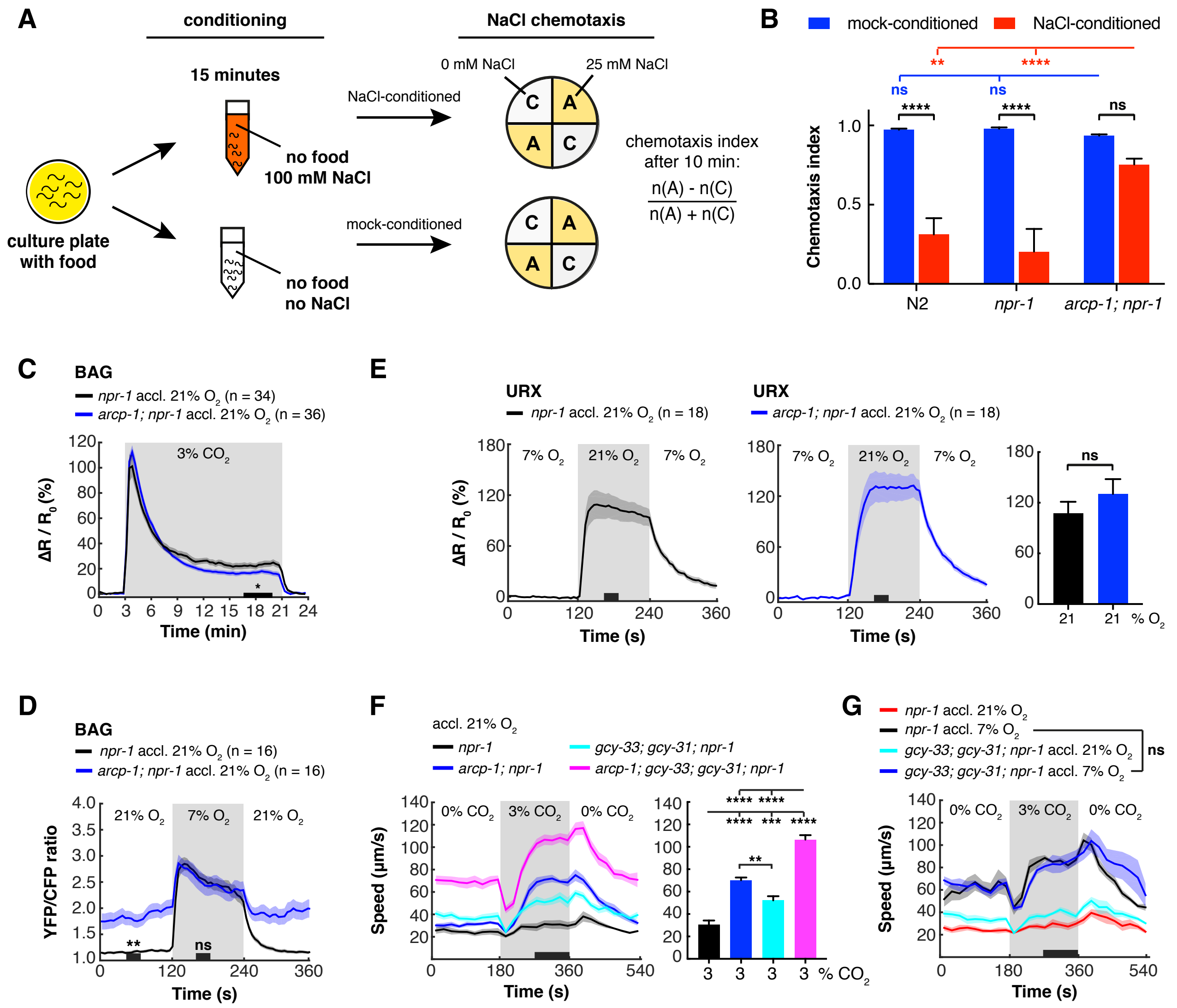
A
conditioning
NaCl chemotaxis
15 minutes
0 mM NaCl
25 mM NaCl
NaCl-conditioned
no food
100 mM NaCl
culture plate
with food
mock-conditioned
no food
no NaCl
chemotaxis index
after 10 min:
n(A) - n(C)
n(A) + n(C)
B
mock-conditioned
NaCl-conditioned
Chemotaxis index
N2
npr-1
arcp-1; npr-1
C
BAG
npr-1 accl. 21% O2 (n = 34)
arcp-1; npr-1 accl. 21% O2 (n = 36)
3% CO2
ΔR / R0 (%)
Time (min)
E
URX
npr-1 accl. 21% O2 (n = 18)
arcp-1; npr-1 accl. 21% O2 (n = 18)
7% O2
21% O2
Time (s)
% O2
D
BAG
npr-1 accl. 21% O2 (n = 16)
arcp-1; npr-1 accl. 21% O2 (n = 16)
YFP/CFP ratio
F
accl. 21% O2
npr-1
arcp-1; npr-1
gcy-33; gcy-31; npr-1
arcp-1; gcy-33; gcy-31; npr-1
0% CO2
Speed (µm/s)
% CO2
G
npr-1 accl. 21% O2
npr-1 accl. 7% O2
gcy-33; gcy-31; npr-1 accl. 21% O2
gcy-33; gcy-31; npr-1 accl. 7% O2

Figure S6, related to Figures 3 and 4. Sensory responses of *arcp-1* mutants to gustatory and O_2 stimuli. A) Overview of salt-based associative learning assay. Worms were washed off their culture plate and conditioned for 15 minutes in the absence of food in a buffer with NaCl (conditioned) or without (mock-conditioned). After training, worms were placed in the center of a quadrant assay plate and their chemotaxis behavior quantified after 10 min. **B)** Chemotaxis index of mock- and NaCl-conditioned *npr-1* and *arcp-1; npr-1* animals. The N2 reference strain was used as a control for gustatory plasticity. *arcp-1; npr-1* animals show a chemotaxis index similar to that of N2 and *npr-1* animals after mock-conditioning, but are defective in the learned aversive response to NaCl after conditioning with salt in the absence of food. Two-way ANOVA with post-hoc Tukey's test. n = 8 assays with 50-100 animals per trial. **C)** Tonic Ca^{2+} response of BAG neurons in response to 3% CO_2, measured using YC3.60. Mean traces of Ca^{2+} activity are plotted as % change in R/R_0, where R is the fluorescence emission ratio at a given time point and R_0 is its initial value. n = number of animals. Mann-Whitney *u* test. **D)** O_2-evoked Ca^{2+} activity in BAG neurons of *npr-1* and *arcp-1; npr-1* animals, as YFP/CFP ratio of the cameleon sensor YC2.60. n = number of animals. Two-way ANOVA with Šidák test. **E)** Mean traces of URX Ca^{2+} activity evoked in *npr-1* and *arcp-1; npr-1* animals by 21% O_2, measured using YC2.60. n = number of animals. Mann-Whitney *u* test. **F)** *gcy-33; gcy-31; npr-1* mutants show increased locomotory arousal at 3% CO_2. *arcp-1; gcy-33; gcy-31; npr-1* quadruple mutants show an additive phenotype, indicating that these genes act in at least partly separate pathways to control CO_2 aversion. n = 10 assays with 20-30 animals per trial. One-way ANOVA with Tukey's test. **G)** *gcy-33; gcy-31; npr-1* animals show normal O_2-dependent modulation of CO_2 escape behavior. Mutant animals respond similarly to *npr-1* control animals when acclimated at 7% O_2. n = 5 assays with 20-30 animals per trial. Mann-Whitney *u* test.

For C-G, solid lines plot mean and shaded areas indicate SEM. Black bars depict time intervals used for statistical comparisons. Bar graphs plot mean ± SEM for these intervals.

* $P < 0.05$; ** $P < 0.01$; *** $P < 0.001$; **** $P < 0.0001$; ns, not significant.

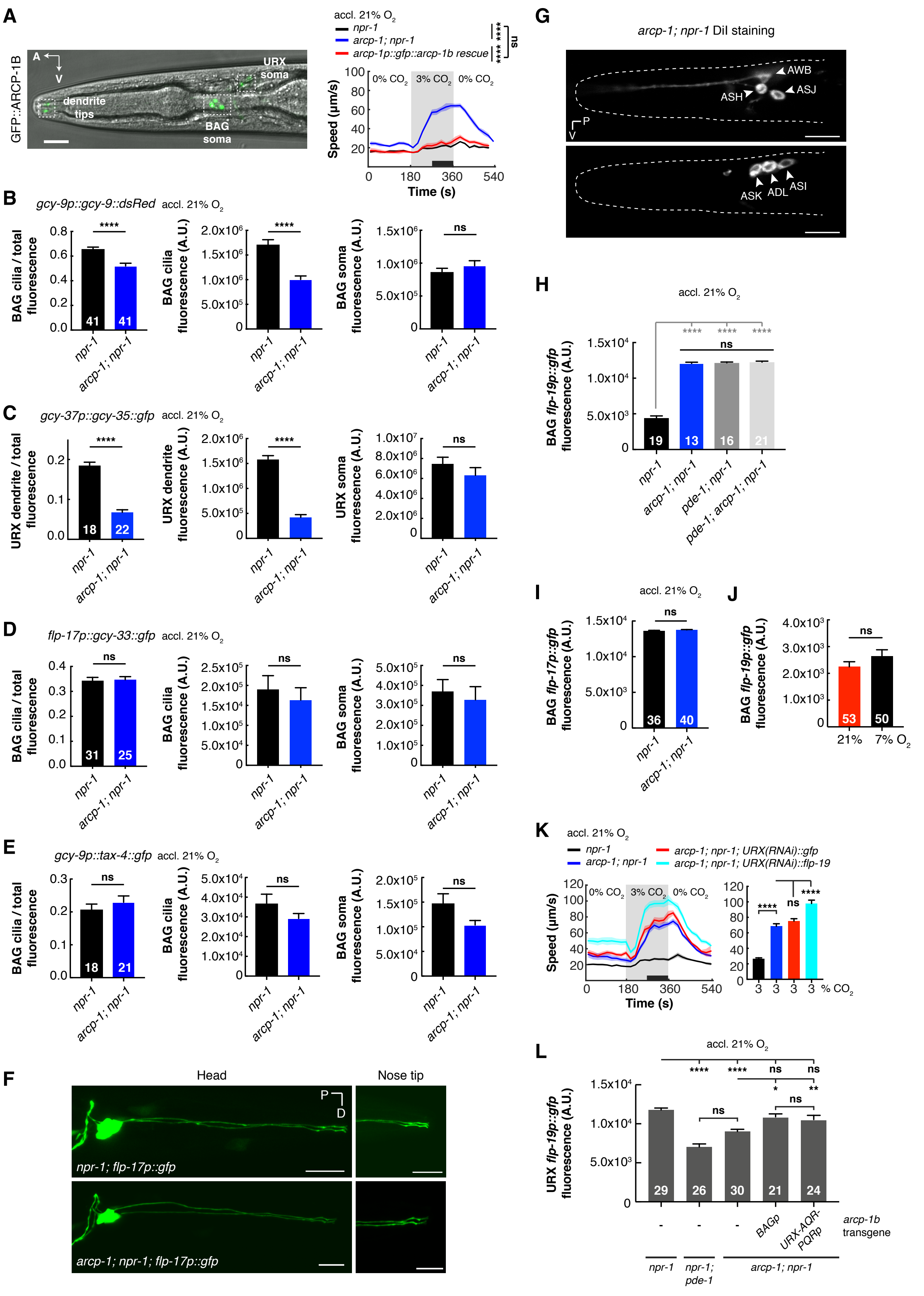
A
GFP::ARCP-1B
dendrite tips
BAG soma
URX soma
accl. 21% O2
npr-1
arcp-1; npr-1
arcp-1p::gfp::arcp-1b rescue
0% CO2 3% CO2 0% CO2
Speed (µm/s)
Time (s)
B
gcy-9p::gcy-9::dsRed accl. 21% O2
BAG cilia / total fluorescence
BAG cilia fluorescence (A.U.)
BAG soma fluorescence (A.U.)
C
gcy-37p::gcy-35::gfp accl. 21% O2
URX dendrite / total fluorescence
URX dendrite fluorescence (A.U.)
URX soma fluorescence (A.U.)
D
flp-17p::gcy-33::gfp accl. 21% O2
E
gcy-9p::tax-4::gfp accl. 21% O2
F
Head
Nose tip
npr-1; flp-17p::gfp
arcp-1; npr-1; flp-17p::gfp
G
arcp-1; npr-1 DiI staining
AWB ASJ ASH ASK ADL ASI
H
accl. 21% O2
BAG flp-19p::gfp fluorescence (A.U.)
npr-1
arcp-1; npr-1
pde-1; npr-1
pde-1; arcp-1; npr-1
I
accl. 21% O2
BAG flp-17p::gfp fluorescence (A.U.)
J
BAG flp-19p::gfp fluorescence (A.U.)
21% 7% O2
K
accl. 21% O2
npr-1
arcp-1; npr-1
arcp-1; npr-1; URX(RNAi)::gfp
arcp-1; npr-1; URX(RNAi)::flp-19
% CO2
L
accl. 21% O2
URX flp-19p::gfp fluorescence (A.U.)
BAGp
URX-AQR-PQRp
arcp-1b transgene
npr-1
npr-1; pde-1
arcp-1; npr-1

Figure S7, related to Figures 5 and 6. Ciliary enrichment of molecular signaling components and regulation of neuropeptide expression in CO_2 and O_2 sensors. A) GFP-tagged ARCP-1B is enriched at the sensory endings of BAG neurons. The *arcp-1p::gfp::arcp-1b* transgene expresses a functional GFP-ARCP-1B protein that rescues the locomotory arousal of *arcp-1; npr-1* animals at 3% CO_2. One-way ANOVA with Tukey test. n ≥ 6 assays with 20-30 animals per trial. **B-E)** Ciliary enrichment of different tagged signaling molecules in the gas sensors, measured as the ratio of fluorescence at the sensory endings versus the total fluorescence in BAG (dendritic ending + soma). GCY-9: BAG CO_2 receptor; TAX-4: cGMP-gated ion channel subunit; GCY-33: BAG O_2 sensor; GCY-35: URX O_2 sensor. n (in bars) = number of animals. Mann-Whitney *u* test. **F)** Maximum intensity projections of confocal Z-stacks of *npr-1* and *arcp-1; npr-1* animals expressing a *flp-17p::gfp* transgene in BAG neurons. *arcp-1* mutants are not overtly defective in dendritic morphology. Scale bar = 10 µm. D = dorsal; P = posterior. **G)** DiI staining of amphid sensory neurons in *arcp-1* mutants shows normal dye filling of ciliated neurons. ARCP-1 is expressed in AWB neurons (see Figure 3C). Scale bar = 10 µm. V = ventral; P = posterior. **H)** *pde-1; arcp-1* double mutants do not show additive phenotypes, suggesting these genes act in the same pathway to regulate *flp-19* expression in BAG. n (in bars) = number of animals. One-way ANOVA with Tukey test. **I)** Expression of a fluorescent reporter for *flp-17* (*flp-17p::gfp*) is not altered by loss of *arcp-1*. n (in bars) = number of animals. Mann-Whitney *u* test. **J)** Previous O_2 experience does not influence BAG expression of a *flp-19* neuropeptide reporter (*flp-19p::gfp)* in *npr-1* animals. n (in bars) = number of animals. Mann-Whitney *u* test. **K)** CO_2-evoked locomotory arousal of *arcp-1; npr-1* mutants following knockdown of *flp-19* expression in URX neurons by expressing sense and antisense sequences from the *gcy-32* promoter. Knockdown of *flp-19* in the mutant background does not prevent locomotory arousal at 3% CO_2, and enhances baseline locomotion in the absence of CO_2; knockdown of *gfp* does not confer any phenotype. One-way ANOVA with Dunnett test. n = 11 - 12 assays with 20-30 animals per trial. **L)** Mean fluorescence ± SEM of a *flp-19p::gfp* reporter in URX, indicating that PDE-1 and ARCP-1 promote *flp-19* expression in these neurons. Cell-specific expression of *arcp-1b* in URX. AQR and PQR, using the *gcy-32* promoter, as well as expression in BAG, using the *flp-17* promoter (*BAGp*), rescues this phenotype. n (in bars) = number of animals. Kruskal-Wallis with Dunn test. For A and K, solid lines depict mean; shaded areas show SEM. Bar graphs plot mean ± SEM. * $P < 0.05$; ** $P < 0.01$; **** $P < 0.0001$; ns, not significant.

Table S1: Strain list, related to STAR Methods.

Strain	Genotype	Notes	Figures
AX1796	*glb-5(Haw) V; npr-1(g320) X*	*glb-5(Haw); npr-1(215F)*	1A-D
LSJ1	*C. elegans* Bristol strain	*glb-5(Haw); npr-1(215F)*	1G-H, S1A-B, S2C-D
RGD1	*Caenorhabditis angaria* wild isolate		S1A-C
VX80	*Caenorhabditis latens* wild isolate		S1A-B, S1D
DF5081	*Caenorhabditis japonica* wild isolate		S1A-B
JU1904	*Caenorhabditis wallacei* wild isolate		S1A-B
JU1373	*Caenorhabditis tropicalis* wild isolate		S1A-B
HK105	*Caenorhabditis briggsae* wild isolate		S1A-B
JU1422	*Caenorhabditis nigoni* wild isolate		S1A-B
JU800	*Caenorhabditis sinica* wild isolate		S1A-B
ED3011	*C. elegans* wild isolate		1B-C, S2A-B
ED3073	*C. elegans* wild isolate		1B-C, S2A-B
EG4946	*C. elegans* wild isolate		1B-C, S2A-B
JU258	*C. elegans* wild isolate		1B-C, S2A-B
JU561	*C. elegans* wild isolate		1B-C, S2A-B
JU1088	*C. elegans* wild isolate		1B-C, S2A-B
JU1248	*C. elegans* wild isolate		1B-C, S2A-B
JU1543	*C. elegans* wild isolate		1B-C, S2A-B
JU2825	*C. elegans* wild isolate		1B-C, 2A, S2A-B, S3A-C, S4
MY16	*C. elegans* wild isolate		1B-E, S2A-B
JU1249	*C. elegans* wild isolate		1B-D, 1F-H, 2B, 2F-G, 2K, 4C, S2A-D, S3A-C, S4, S5A-B
AX613	*npr-1(g320) X*	*npr-1(215F)*	2G, 2K, 4C
JU3221	*arcp-1(mfP22) III; npr-1(215F) X; mfEx94 [arcp-1p::arcp-1b::sl2::gfp; myo-2p::dsRed2]* in JU1249 background		2G, 2K
AX204	*npr-1(ad609) X*		2G, 2I-J, 3D, 4D-E, 5F, 6C-G, S5C-I, S6B, S6F-G, S7A, S7K
AX6574<br>AX7324	*arcp-1(db1082) III; npr-1(ad609) X* 4x outcrossed<br>*arcp-1(db1082) III; npr-1(ad609) X* 5x outcrossed		2F-J, 3D, 4D-E, 5F, 6C-E, S5A-F, S5H-I, S6B, S6F, S7A, S7K

AX6723	*arcp-1(db1082) III; npr-1(ad609) X; dbEx975 [arcp-1p::arcp-1b::sl2::gfp; unc-122p::rfp]*	*arcp-1p::arcp-1b*	2G, 2J
AX7094	*arcp-1(db1082) III; npr-1(ad609) X; dbEx1050 [arcp-1p::arcp-1b::sl2::mKate; lin-44p::gfp]*	*arcp-1p::arcp-1b*	2I, S5H-I
AX6720	*arcp-1(db1082) III; npr-1(ad609) X; dbEx974 [arcp-1p::arcp-1a::sl2::gfp; unc-122p::rfp]*	*arcp-1p::arcp-1a*	2J
N2	*C. elegans* Bristol strain		S3C, S6B
MY10	*C. elegans* wild isolate		S3C
JU1247	*C. elegans* wild isolate		S3C
AX6901	*arcp-1(db1082) III; npr-1(ad609) X; dbEx1002 [fosmid arcp-1p::arcp-1::sl2::gfp; unc-122p::rfp]*		3C
AX6766	*arcp-1(db1082) III; npr-1(ad609) X; dbEx984 [gcy-32p::arcp-1b::sl2::gfp; unc-122p::rfp]*	*URX-AQR-PQRp::arcp-1b*	3D, 4D
AX6805	*arcp-1(db1082) III; npr-1(ad609) X; dbEx990 [flp-17p::arcp-1b::sl2::gfp; unc-122p::rfp]*	*BAGp::arcp-1b*	3D, 4D, S5H-I
AX6931	*arcp-1(gk856856) III; npr-1(ad609) X*		S5B-C
AX6929	*arcp-1(gk863317) III; npr-1(ad609) X*		S5B-C
AX6927	*arcp-1(gk852871) III; npr-1(ad609) X*		S5B-C, S5G
AX7023	*arcp-1(db1082) III; npr-1(ad609) X; dbEx1035 [gcy-32p::arcp-1b::sl2::mKate; lin-44p::gfp]*	*URX-AQR-PQRp::arcp-1b*	S5H-I
AX7095	*arcp-1(db1082) III; npr-1(ad609) X; dbEx990 [flp-17p::arcp-1b::sl2::gfp; unc-122p::rfp]; dbEx1035 [gcy-32p::arcp-1b::sl2::mKate; lin-44p::gfp]*	*BAGp + URX-AQR-PQRp*	S5H-I
AX7179	*gcy-9(n4470) npr-1(ad609) X*		4E
AX7238	*arcp-1(db1082) III; gcy-9(n4470) npr-1(ad609) X*		4E
AX7116	*arcp-1(db1082) III; npr-1(ad609) X; dbIs20 [arcp-1p::gfp::arcp-1b; unc-122p::rfp]*	GFP-ARCP-1B	5B-C, S7A
AX6969	*malt-1(db1194) II; npr-1(ad609) X; dbIs16 [rab-3p::malt-1::gfp; unc-122p::rfp]*	(1) GFP control	5B
AX7082	*eif-3.L(db1015) II; npr-1(ad609) X; dbIs19 [rab-3p::eif-3.L::gfp; unc-122p::rfp]*	(2) GFP control	5B
AX7419	*npr-1(ad609) X dbEx1075 [flp-17p::pde-1b::gfp; unc-122p::rfp]*	PDE-1B-GFP	5D-E
AX7422	*arcp-1(db1082) III; npr-1(ad609) X; dbEx1075 [flp-17p::pde-1b::gfp; unc-122p::rfp]*		5E
AX2272	*pde-1(ok2924) I; npr-1(ad609) X*		5F
AX7453	*pde-1(ok2924) I; arcp-1(db1082) III; npr-1(ad609) X*		5F
AX6881	*npr-1(ad609) X dbEx [flp-17p::YC3.60]*		4A-B, S6C
AX6893	*arcp-1(db1082) III; npr-1(ad609) X; dbEx [flp-17p::YC3.60]*		4A-B, S6C
AX7842	*arcp-1(db1082) III; npr-1(ad609) X; dbEx [flp-17p::YC3.60]; dbEx1035 [gcy-32p::arcp-1b::sl2::mKate; lin-44::gfp]*	*URX-AQR-PQRp::arcp-1b*	4B
AX7845	*arcp-1(db1082) III; npr-1(ad609) X; dbEx [flp-17p::YC3.60] dbEx1172 [gcy-33p::arcp-1b::sl2::mKate; unc-122p::gfp]*	*BAGp::arcp-1b*	4B
AX3516	*npr-1(ad609) X; dbEx614 [gcy-37p::YC2.60; unc-122p::rfp]*		S6E
AX6877	*arcp-1(db1082) III; npr-1(ad609) X; dbEx614 [gcy-37p::YC2.60; unc-122p::rfp]*		S6E
AX3432	*npr-1(ad609) X; dbEx623 [flp-17p::YC2.60; F15E11.1::mCherry]*		S6D
AX7182	*arcp-1(db1082) III; npr-1(ad609) X; dbEx623 [flp-17p::YC2.60; F15E11.1::mCherry]*		S6D
AX7656	*gcy-33(ok232) V; gcy-31(ok296) npr-1(ad609) X*		S6F-G
AX7657	*arcp-1(db1082) III; gcy-33(ok232) V; gcy-31(ok296) npr-1(ad609) X*		S6F
AX7362	*npr-1(ad609) X; wzIs132 [gcy-9p::gcy-9::dsRed]*		S7B

AX7361	*arcp-1(db1082) III; npr-1(ad609) X; wzIs132 [gcy-9p::gcy-9::dsRed]*		S7B
AX7366	*npr-1(ad609) X; wzEx156 [gcy-9p::tax-4::gfp]*		S7E
AX7365	*arcp-1(db1082) III; npr-1(ad609) X; wzEx156 [gcy-9p::tax-4::gfp]*		S7E
AX2997	*gcy-33(ok232) V; npr-1(ad609) X; dbEx [flp-17p::gcy-33::gfp; unc-122p::rfp]*		S7D
AX7315	*arcp-1(db1082) III; gcy-33(ok232) V; npr-1(ad609) X; dbEx [flp-17p::gcy-33::gfp; unc-122p::rfp]*		S7D
AX6516	*npr-1(ad609) X; dbEx1053 [gcy-37p::gcy-35::HA::gfp::sl2::mCherry]*		S7C
AX7278	*arcp-1(db1082) III; npr-1(ad609) X; dbEx1053 [gcy-37p::gcy-35::HA::gfp::sl2::mCherry]*		S7C
AX7019	*arcp-1(db1082) III; npr-1(ad609) X; dbEx1033 [flp-17p::gfp; unc-122p::rfp]*		S7F
AX7021	*npr-1(ad609) X; dbEx1033 [flp-17p::gfp; unc-122p::rfp]*		S7F
AX7268	*npr-1(ad609) X; ynIs34 [flp-19p::gfp]*		6A, S7H, S7J, S7L
AX7271	*arcp-1(db1082) III; npr-1(ad609) X; ynIs34 [flp-19p::gfp]*		6A, S7H, S7L
AX7279	*pde-1(ok2924) I; npr-1(ad609) X; ynIs34 [flp-19p::gfp]*		6A, S7H, S7L
AX7272	*arcp-1(db1082) III; npr-1(ad609) X; ynIs34 [flp-19p::gfp]; dbEx1063 [flp-17p::arcp-1b::sl2::mKate; unc-122p::gfp]*	*BAGp::arcp-1b*	6A, S7L
AX7273	*arcp-1(db1082) III; npr-1(ad609) X; ynIs34 [flp-19p::gfp] dbEx1035 [gcy-32p::arcp-1b::sl2::mKate; lin-44::gfp]*	*URX-AQR-PQRp::arcp-1b*	6A, S7L
AX7550	*pde-1(ok2924) I; arcp-1(db1082) III; npr-1(ad609) X; ynIs34 [flp-19p::gfp]*		S7H
AX7722	*ynIs34 [flp-19p::gfp]* backcrossed 10x in JU2825 background		6B
AX7724	*ynIs34 [flp-19p::gfp]* backcrossed 10x in JU1249 background		6B
AX7726	*dbEx1063 [flp-17p::arcp-1b::sl2::mKate; unc-122p::gfp]; ynIs34 [flp-19p::gfp]* backcrossed 10x in JU1249 background		6B
AX7210	*npr-1(ad609) X; ynIs64 [flp-17p::gfp]*		S7I
AX7208	*arcp-1(db1082) III; npr-1(ad609) X; ynIs64 [flp-17p::gfp]*		S7I
AX7321	*flp-19(ok2460) npr-1(ad609) X*		6C
AX7322	*arcp-1(db1082) III; flp-19(ok2460) npr-1(ad609) X*		6C
AX7754	*arcp-1(db1082) III; npr-1(ad609) X; dbEx1171 [gcy-33p::gfp (sas); unc-122p::rfp]*	*BAG(RNAi)::gfp*	6D-E
AX7760	*arcp-1(db1082) III; npr-1(ad609) X; dbEx1173 [gcy-33p::flp-19 (sas); unc-122p::rfp]*	*BAG(RNAi)::flp-19*	6D-E
AX7788	*arcp-1(db1082) III; npr-1(ad609) X; dbEx1178 [gcy-32p::gfp (sas); unc-122p::rfp]*	*URX(RNAi)::gfp*	S7K
AX7678	*arcp-1(db1082) III; npr-1(ad609) X; dbEx1153 [gcy-32p::flp-19 (sas); unc-122p::gfp]*	*URX(RNAi)::flp-19*	S7K
AX7793	*npr-1(ad609) X; dbEx1173 [gcy-33p::flp-19 (sas); unc-122p::rfp]*	*BAG(RNAi)::flp-19*	6F
AX7437	*npr-1(ad609) X; dbEx1077 [flp-17p::flp-19::sl2::mKate; unc-122p::gfp]*	*BAGp::flp-19*	6G

Table S2, related to STAR Methods. List of primers used in this study.

PRIMER	SOURCE	IDENTIFIER
npr-1 genotyping Fw<br>CTCCACCATTTTGCGTATCTTTGT	(Duveau and Félix, 2012)	N/A
npr-1 genotyping Rev<br>AGGAAGAGAGAATATGCGGCTAC	(Duveau and Félix, 2012)	N/A
glb-5 genotyping Fw<br>GGTGACTCGTTGGATGATGA	(McGrath et al., 2009)	N/A
glb-5 genotyping Rev1<br>GTGATGAGCTCCCATTTCGT	(McGrath et al., 2009)	N/A
glb-5 genotyping Rev2<br>TTCTGCTCCAGAAGACACGA	(McGrath et al., 2009)	N/A
nath-10 genotyping Fw<br>GCCGGGAACGAGGAAAAGTCAAATG	(Duveau and Félix, 2012)	N/A
nath-10 genotyping Rev<br>TTCGGACTCACTGTTCCACTTTTA	(Duveau and Félix, 2012)	N/A
arcp-1 genotyping Fw<br>AAACGCAGTTGCAGATTGAA	This paper	N/A
arcp-1 genotyping Rev1<br>TCGATCGATGAAAGACAACG	This paper	N/A
arcp-1 genotyping Rev2<br>GAATTGGTGCTTACCGGATT	This paper	N/A
arcp-1a cDNA cloning Fw<br>GGGGACAAGTTTGTACAAAAAAGCAGGCTTTTCAGAAAA**ATGCGACCACCTCCACTTGTTC**	This paper	N/A
arcp-1a cDNA cloning Rev<br>GGGGACCACTTTGTACAAGAAAGCTGGGT**TCAATGACTTCCATTGTGACGTGG**	This paper	N/A
arcp-1b cDNA cloning Fw<br>GGGGACAAGTTTGTACAAAAAAGCAGGCTTTTCAGAAAA**ATGCATGAGCGTCTACCATTTGATC**	This paper	N/A
arcp-1b cDNA cloning Rev<br>GGGGACCACTTTGTACAAGAAAGCTGGGT**TCAATGACTTCCATTGTGACGTGG**	This paper	N/A
pde-1b cDNA cloning Fw<br>GGGGACAAGTTTGTACAAAAAAGCAGGCTTTTCAGAAAA**ATGATGATCGTCGGGGATACC**	This paper	N/A
pde-1b cDNA cloning Rev<br>GGGGACCACTTTGTACAAGAAAGCTGGGT**AATTCGTTGTGACGCCATTTTC**	This paper	N/A
flp-19 cDNA cloning Fw<br>GGGGACAAGTTTGTACAAAAAAGCAGGCTTTTCAGAAAA**ATGTCCTTCCAGCTAACGCTGT**	This paper	N/A
flp-19 cDNA cloning Rev<br>GGGGACCACTTTGTACAAGAAAGCTGGGT**TTATCCGAACCTGACTGAGCTGG**	This paper	N/A

III_663310 pyrosequencing Fw<br>GTGACGTACTAGCAACGAGTCGATTTTGGGGATGGA	This paper	N/A
III_663310 pyrosequencing Rev<br>CACTAGGCAGGTAGGCATTTTT	This paper	N/A
III_663310 pyrosequencing Sequencing<br>CCAAACTTTTATAGAGATCA	This paper	N/A
Universal biotinylated primer for pyrosequencing<br>[Btn]TAGCAGGATACGACTATC	(Richaud et al., 2018)	N/A
Oligo-dT_{30} VN for RNA-Seq<br>AAGCAGTGGTATCAACGCAGAGTACT_{30}VN where "N" is any base and "V" is either A, C or G	(Picelli et al., 2014)	N/A
TSO for RNA-Seq<br>AAGCAGTGGTATCAACGCAGAGTACATrGrG+G with "rG' riboguanosine and "+G" LNA-modified guanosine	(Picelli et al., 2014)	N/A
IS PCR primer for RNA-Seq<br>AAGCAGTGGTATCAACGCAGAGT	(Picelli et al., 2014)	N/A
