# Supplementary material for: Natural Variation in a Dendritic Scaffold Protein Remodels Experience-Dependent Plasticity by Altering Neuropeptide Expression
Source: Neuron. 2020 Jan 8;105(1):106–121.e10. doi: 10.1016/j.neuron.2019.10.001 (PMC6953435; doi:10.1016/j.neuron.2019.10.001)
Supplement: Document S2. Article plus Supplemental Information [file mmc6.pdf]

Article

Neuron

Natural Variation in a Dendritic Scaffold Protein Remodels Experience-Dependent Plasticity by Altering Neuropeptide Expression

Highlights

- Behavioral flexibility varies across *Caenorhabditis* and *C. elegans* wild isolates
- A natural polymorphism in ARCP-1 underpins inter-individual variation in plasticity
- ARCP-1 is a dendritic scaffold protein localizing cGMP signaling machinery to cilia
- Disrupting ARCP-1 alters behavioral plasticity by changing neuropeptide expression

Authors

Isabel Beets, Gaotian Zhang, Lorenz A. Fenk, Changchun Chen, Geoffrey M. Nelson, Marie-Anne Félix, Mario de Bono

Correspondence

felix@biologie.ens.fr (M.-A.F.), mdebono@ist.ac.at (M.d.B.)

In Brief

Individuals can vary in their capacity to adapt their behavior to changes in the environment. Beets et al. identify a natural genetic polymorphism that modifies behavioral flexibility by altering the neuromodulatory output of primary sensory neurons.

Beets et al., 2020, Neuron *105*, 106–121
January 8, 2020 © 2019 MRC Laboratory of Molecular Biology. Published by Elsevier Inc.
https://doi.org/10.1016/j.neuron.2019.10.001

CellPress

Natural Variation in a Dendritic Scaffold Protein Remodels Experience-Dependent Plasticity by Altering Neuropeptide Expression

Isabel Beets,[1] Gaotian Zhang,[2] Lorenz A. Fenk,[1] Changchun Chen,[1] Geoffrey M. Nelson,[1] Marie-Anne Félix,[2,*] and Mario de Bono[1,3,4,*]
[1]Cell Biology Division, MRC Laboratory of Molecular Biology, Cambridge CB2 0QH, UK
[2]Institut de Biologie de l'École Normale Supérieure, CNRS, Inserm, PSL Research University, Paris 75005, France
[3]Present address: IST Austria, Klosterneuburg 3400, Austria
[4]Lead Contact
*Correspondence: felix@biologie.ens.fr (M.-A.F.), mdebono@ist.ac.at (M.d.B.)
https://doi.org/10.1016/j.neuron.2019.10.001

SUMMARY

The extent to which behavior is shaped by experience varies between individuals. Genetic differences contribute to this variation, but the neural mechanisms are not understood. Here, we dissect natural variation in the behavioral flexibility of two *Caenorhabditis elegans* wild strains. In one strain, a memory of exposure to 21% O_2 suppresses CO_2-evoked locomotory arousal; in the other, CO_2 evokes arousal regardless of previous O_2 experience. We map that variation to a polymorphic dendritic scaffold protein, ARCP-1, expressed in sensory neurons. ARCP-1 binds the Ca^{2+}-dependent phosphodiesterase PDE-1 and co-localizes PDE-1 with molecular sensors for CO_2 at dendritic ends. Reducing ARCP-1 or PDE-1 activity promotes CO_2 escape by altering neuropeptide expression in the BAG CO_2 sensors. Variation in ARCP-1 alters behavioral plasticity in multiple paradigms. Our findings are reminiscent of genetic accommodation, an evolutionary process by which phenotypic flexibility in response to environmental variation is reset by genetic change.

INTRODUCTION

Animals reconfigure their behavior and physiology in response to experience, and many studies highlight mechanisms underlying such plasticity (Bargmann, 2012; Owen and Brenner, 2012). While plasticity is presumed crucial for evolutionary success, it has costs and often varies across species and between individuals (Coppens et al., 2010; Dewitt et al., 1998; Mery, 2013; Niemelä et al., 2013). Variation in behavioral flexibility is thought to underlie inter-individual differences in cognitive ability and capacity to cope with environmental challenges (Coppens et al., 2010; Niemelä et al., 2013). The genetic and cellular basis of inter-individual variation in experience-dependent plasticity is, however, poorly understood.

Genetic accommodation and assimilation are concepts used to describe variation in plasticity on an evolutionary timescale. Waddington and Schmalhausen suggested genetic assimilation occurs when a phenotype initially responsive to the environment becomes fixed in a specific state (Renn and Schumer, 2013; Schmalhausen, 1949; Waddington, 1942, 1953). This loss of plasticity may reflect genetic drift or selection against the costs of expressing adaptive behaviors (Niemelä et al., 2013). Studies of genetic assimilation led to the broader concept of genetic accommodation, referring to evolutionary genetic variation leading to any change in the environmental regulation of a phenotype (Crispo, 2007; West-Eberhard, 2005). Many studies in insects, fish, rodents, and primates highlight inter-individual variation in behavioral plasticity; in some cases this has been shown to be heritable (Dingemanse and Wolf, 2013; Izquierdo et al., 2007; Mery et al., 2007), but the mechanisms responsible for these differences remain enigmatic.

Many animals use gradients of respiratory gases to help locate prey, mates, or predators and have evolved sophisticated behavioral responses to environmental changes in oxygen (O_2) and carbon dioxide (CO_2) levels (Carrillo and Hallem, 2015; Cummins et al., 2014; Guerenstein and Hildebrand, 2008; Prabhakar and Semenza, 2015). Where studied, behavioral responses to CO_2 have been shown to depend on environmental context, past experience, and life stage (Carrillo et al., 2013; Fenk and de Bono, 2017; Guillermin et al., 2017; Hallem and Sternberg, 2008; Sachse et al., 2007; Vulesevic et al., 2006). This flexibility makes CO_2-sensing an attractive paradigm to study natural variation in behavioral plasticity.

CO_2 responses in *Caenorhabditis elegans* are sculpted by previous O_2 experience (Carrillo et al., 2013; Fenk and de Bono, 2017; Kodama-Namba et al., 2013). Acclimation to surface O_2 levels (i.e., 21%) generates a memory that suppresses aversion of high CO_2. The O_2 memory is written over hours by O_2 sensors, called URX, AQR, and PQR, whose activity is tonically stimulated by 21% O_2 (Busch et al., 2012; Fenk and de Bono, 2017). 21% O_2 is itself aversive to *C. elegans*, most likely because it signals surface exposure (Gray et al., 2004; Persson et al., 2009). By suppressing CO_2 aversiveness, *C. elegans* acclimated to 21% O_2 may increase their chance of escaping the surface into buried environments with elevated CO_2 (Fenk and de Bono, 2017).

 © 2019 MRC Laboratory of Molecular Biology. Published by Elsevier Inc.
This is an open access article under the CC BY license (http://creativecommons.org/licenses/by/4.0/).

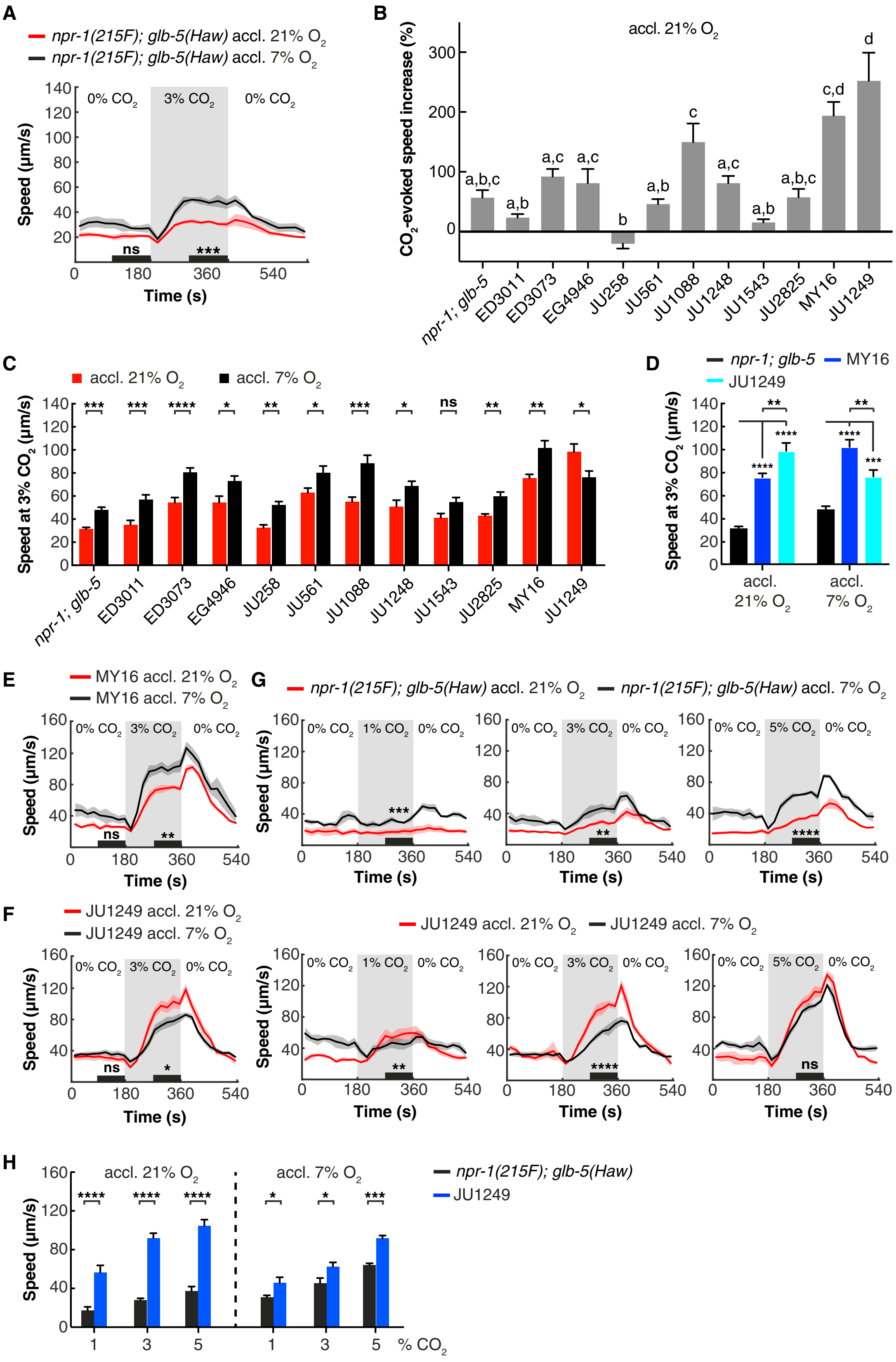

(legend on next page)

Here, we show that the impact of O_2 experience on CO_2 aversion varies across *Caenorhabditis* species and between wild *C. elegans* isolates. By characterizing differences between *C. elegans* isolates, we identify a polymorphism in a dendritic ankyrin-repeat scaffold protein, ARCP-1, that alters plasticity in one strain. ARCP-1 biochemically interacts with the conserved cyclic nucleotide phosphodiesterase PDE-1 and localizes it with molecular sensors for CO_2 to the dendritic ends of BAG sensory neurons. Disrupting ARCP-1 resets CO_2 sensitivity and experience-dependent plasticity of CO_2 escape, in part by altering neuropeptide expression and conferring strong aversion to CO_2.

RESULTS

Natural Variation in Experience-Dependent Plasticity in *Caenorhabditis*

In *C. elegans*, a memory of recent O_2 levels reprograms aversive responses to CO_2 (Fenk and de Bono, 2017). We hypothesized this experience-dependent plasticity is evolutionarily variable. To investigate this, we compared the CO_2 responses of different *Caenorhabditis* species grown at 21% or 7% O_2 (Figure S1A). Animals were transferred to a thin bacterial lawn in a microfluidic chamber kept at 7% O_2, stimulated with 3% CO_2, and their behavioral responses quantified. We used a background level of 7% O_2 in all assays because *C. elegans* dwell locally at this O_2 concentration, making locomotory arousal by CO_2 prominent. By contrast, 21% O_2 evokes sustained rapid movement, making CO_2 responses above this high baseline proportionally small. As a representative *C. elegans* strain, we used LSJ1, a wild-type (N2-like) laboratory strain bearing natural alleles of the neuropeptide receptor *npr-1*(*215F*) and the neuroglobin *glb-5*(*Haw*). We did not use the standard N2 strain, because it has acquired mutations in *npr-1* and *glb-5* that confer gas-sensing defects (McGrath et al., 2009; Persson et al., 2009). As expected, *C. elegans* was aroused more strongly by CO_2 when acclimated to 7% O_2 (Figure S1B). By contrast, O_2 experience did not alter the absolute speed of representative strains of *C. latens* and *C. angaria* at 3% CO_2 (Figure S1B). Because *C. angaria* was not aroused by 3% CO_2, we tested its response to 5% and 10% CO_2. These levels evoked locomotory arousal that, as in *C. elegans*, was stronger in animals acclimated to 7% O_2 (Figure S1C). Thus, *C. angaria* is less sensitive to CO_2 than *C. elegans*, but its arousal by CO_2 remains dependent on O_2 experience. By contrast, CO_2 responses of *C. latens* were unaffected by previous O_2 experience at any concentration tested (Figure S1D). Unexpectedly, acclimation to 7% O_2 suppressed rather than enhanced the locomotory response of *C. nigoni* to CO_2 (Figure S1B). Thus, the effect of O_2 memory on CO_2-evoked behavioral responses is evolutionarily variable.

Effect of O_2 Memory on CO_2 Responses Varies between *C. elegans* Wild Isolates

Our findings prompted us to seek intra-species variation in how O_2 experience influences CO_2 responses, by studying a genetically diverse collection of wild *C. elegans* isolates (Figure S2A). Most strains responded like the reference strain (Figures 1A–1C and S2A). However, two isolates, the French JU1249 and German MY16 strains, responded more strongly than other isolates to a rise in CO_2 regardless of O_2 experience (Figures 1B and 1D). For MY16 CO_2 aversion was stronger when animals were acclimated to 7% O_2, recapitulating the cross-modulation of CO_2 responses observed in most strains (Figures 1C and 1E). By contrast, JU1249 animals acclimated to 21% O_2 further enhanced rather than suppressed CO_2 escape (Figures 1C and 1F). To probe further if the O_2-dependent plasticity of CO_2 escape had changed in JU1249, we quantified escape responses at different CO_2 concentrations. *npr-1; glb-5* control animals always responded more strongly to CO_2 when acclimated to 7% O_2, but this was not the case for JU1249 at any CO_2 concentration tested (Figure 1G). CO-evoked arousal was stronger in JU1249 animals acclimated to 21% O_2 than in those acclimated to 7% O_2 (Figure 1H), suggesting that JU1249 fails to suppress CO_2 escape at 21% O_2.

The increased locomotory arousal of JU1249 and MY16 in response to CO_2 could reflect reduced inhibitory input from the neural circuit signaling 21% O_2. To probe this, we asked if these isolates show altered behavioral responses to 21% O_2. All isolates we tested responded similarly when we switched O_2 from 7% to 21% (Figure S2B), suggesting they retained a functional O_2-sensing circuit.

Figure 1. Natural Variation in the Regulation of CO_2 Escape by Previous O_2 Experience

(A) A *C. elegans* reference strain is more strongly aroused by CO_2 when acclimated to 7% rather than 21% O_2. Two-way ANOVA with Šidák test; n = 6 assays. In this, and all subsequent figures, the background O_2 level in the assay is 7%.

(B) Natural variation in the CO_2 response of *C. elegans* wild isolates acclimated to 21% O_2. Bars represent average increase in speed ± SEM when CO_2 rises from 0% to 3%. The CO_2-evoked speed increase is significantly different ($p < 0.05$) between isolates labeled with different letters (a–d). One-way ANOVA with Tukey test; n = 6 assays.

(C) The effect of O_2 memory on CO_2 responses in wild *C. elegans* isolates. Bars show mean ± SEM for time intervals indicated in (A) and Figure S2A. Two-way ANOVA with Šidák test; n = 6 assays.

(D) JU1249 and MY16 are more strongly aroused by CO_2, regardless of previous O_2 experience. Bars plot mean ± SEM. Two-way ANOVA with Tukey test; n = 6 assays.

(E and F) CO_2 responses of MY16 (E) and JU1249 (F) animals acclimated to 21% or 7% O_2. Two-way ANOVA with Šidák test; n = 6 assays.

(G) Acclimation to 21% O_2 in JU1249, unlike the reference strain LSJ1, enhances rather than suppresses locomotory arousal at different CO_2 concentrations. n = 30–61 animals for *npr-1; glb-5*, n = 59–66 animals for JU1249. Mann-Whitney U test.

(H) CO_2 arousal is increased more strongly in JU1249 animals acclimated to 21% rather than 7% O_2. Bars plot mean ± SEM for time intervals indicated in (G). Two-way ANOVA with Šidák test. n = 4 assays.

For (A), (E), and (F), solid lines plot mean and shaded areas show SEM. Black bars indicate time intervals used for statistical comparisons. For (A)–(H), 20–30 animals were assayed in at least 4 trials for each condition. *$p < 0.05$; **$p < 0.01$; ***$p < 0.001$; ****$p < 0.0001$; ns, not significant.

See also Figures S1 and S2.

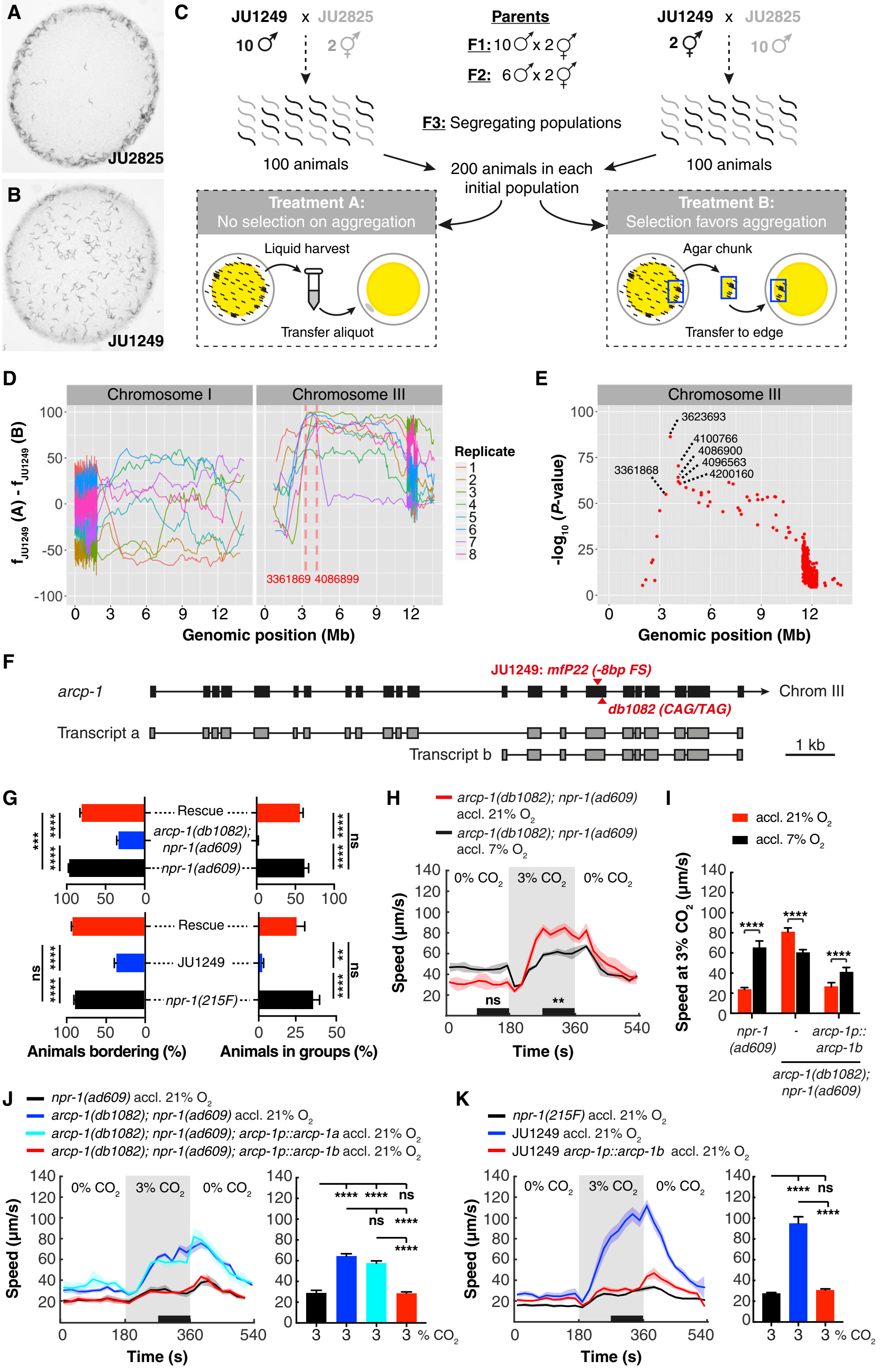

(legend on next page)

In our assays, we exposed animals acclimated to 21% O_2 to a downshift to 7% O_2 3 min before the CO_2 stimulus. To ask if this drop in O_2, rather than O_2 experience, altered the CO_2 response in JU1249, we extended the time animals spent at 7% O_2 prior to receiving the CO_2 stimulus to 24 min. JU1249 was still more strongly aroused by CO_2 when acclimated to 21% rather than 7% O_2; as expected, O_2 experience had the opposite effect on plasticity in *npr-1; glb-5* controls (Figure S2C). We also compared the behavioral responses of JU1249 and *npr-1; glb-5* animals to a 21% to 7% O_2 stimulus and found no significant differences (Figure S2D). Thus, the ability of an O_2 memory to modify CO_2 escape appears to be altered in JU1249, recapitulating the phenotype observed in *C. nigoni*.

Natural Variation in the Ankyrin Repeat Protein ARCP-1 Alters Plasticity of CO_2 Responses

We sought the genetic changes conferring altered plasticity of CO_2 responses in JU1249. Besides altering this phenotype, JU1249 exhibited reduced aggregation and bordering behavior on an *E. coli* food lawn compared to other *C. elegans* wild isolates (Figures 2A and 2B). We speculated JU1249 aggregated poorly because increased avoidance of CO_2 shifted the balance between attraction and repulsion as aerobic animals come together. In this model, the aggregation phenotype, which is easy to score, is linked to altered JU1249 CO_2 responses.

Before testing this hypothesis, we ruled out the possibility that JU1249 is genetically contaminated by the non-aggregating N2 lab strain, by genotyping the *npr-1*, *glb-5*, and *nath-10* loci, which have acquired polymorphisms during N2 domestication (Duveau and Félix, 2012; McGrath et al., 2009; Persson et al., 2009; Weber et al., 2010). JU1249 exhibited the natural alleles found in other wild isolates at all three loci (Figure S3).

To map the JU1249 aggregation defect, we used a selection-based quantitative trait locus (QTL) mapping approach in which we crossed JU1249 to the aggregating *C. elegans* wild isolate JU2825 (Figure 2A). To find conditions for selection-based QTL mapping, we first defined two treatments that differentially selected for aggregating and solitary animals and performed competition tests between JU1249 and JU2825 under these treatments. Starting with a 50:50% mix of each strain, JU1249 (solitary) outcompeted JU2825 (aggregating) when the populations were transferred by liquid harvest and aliquot (Figure S4A, treatment A), indicating that JU1249 has higher fitness in these conditions than JU2825. When cultivated by transferring an agar chunk from the border of the food lawn, where aggregating animals accumulate (Figure S4A, treatment B), JU2825 outcompeted JU1249, which indicates the aggregation trait in *C. elegans* is selectable. We used treatments A and B as selection regimes on populations of cross-progenies of JU1249 and JU2825 (Figure 2C), sequenced their genomes, and compared allele frequencies of paired replicate populations under the two treatments (Data S1; STAR Methods). Populations selected for aggregation (treatment B) were expected to have higher frequencies of JU2825 alleles at the QTL that affect the variation in aggregation behavior compared to the paired populations (treatment A). Our analysis showed large variation in allele frequencies among replicates, suggesting founder effects due to the moderate population sizes in the first crosses (Figures 2D and S4B). We used two criteria to identify candidate QTL regions associated with the aggregation phenotype. First, we identified regions that show consistent differences in allele frequencies among all replicate pairs for the two treatments (Figures S4B and S4C). Second, we narrowed down these regions by examining replicates for the position of the closest recombination event that was selected (Figure S4D). Based on these criteria, we identified a genomic interval on chromosome III (3361869–4086899 bp) as a candidate region, showing a highly significant difference in allele frequencies among the eight population pairs (Figures 2E and S4C).

The 725 kb QTL region in JU1249 contained 3 polymorphisms in protein-coding genes compared to N2 and JU2825 (Data S1J). An 8 bp deletion (*mfP22*) in the open reading frame of the gene F34D10.6, which we named *arcp-1* (for ankyrin repeat containing protein, see below), stood out as a promising candidate for two reasons. First, *mfP22* is the only polymorphism predicted to abolish protein function (Data S1J and S1K), introducing a

Figure 2. Natural Variation in ARCP-1 Alters CO_2 Responses

(A and B) Individuals of JU2825, like most *C. elegans* wild isolates, aggregate at the border of an *E. coli* lawn (A). By contrast, JU1249 animals disperse across the lawn (B).

(C) Selection-based QTL mapping approach to establish the genetic basis of solitary behavior in JU1249.

(D) Line plots showing differences in JU1249 allele frequencies between treatment A and B for each replicate pair, using a sliding window 5 single-nucleotide polymorphisms (SNPs) wide and a step size of one SNP. Replicates are indicated by different colors. Chromosome I shows little consistent deviations from equal frequencies in the two treatments, whereas chromosome III shows a strong enrichment at the 3–4 Mb interval.

(E) Read-count frequency differences between treatment A and B analyzed for consistency across eight replicates using the Cochran-Mantel-Haenszel test. Only chromosome III is shown. p values are shown as $-\log_{10}$ (p value) adjusted by the Bonferroni correction.

(F) Gene structure of *arcp-1* (F34D10.6). Boxes represent exons and lines indicate introns. The wild isolate JU1249 has an 8 bp deletion that introduces a frameshift. The *db1082* allele, isolated in a genetic screen for aggregation-defective mutants, replaces a Gln codon with a premature stop codon.

(G) Wild-type *arcp-1b* rescues bordering and aggregation phenotypes of JU1249 and *db1082* animals. For each assay, 50–60 animals were transferred to a bacterial lawn and behaviors were scored after 6 h. One-way ANOVA with Tukey test. $n \geq 6$ assays.

(H) *arcp-1*(*db1082*) animals, like JU1249, fail to suppress CO_2 responses when acclimated to 21% O_2. n = 5–6 assays. Two-way ANOVA with Šidák test.

(I) Expressing wild-type *arcp-1* restores the O_2-dependent modulation of CO_2 responses in *arcp-1; npr-1* mutants. n = 67–105 animals. Mann-Whitney U test.

(J) An *arcp-1b* transgene, but not *arcp-1a*, rescues the enhanced locomotory arousal evoked by CO_2 in *arcp-1; npr-1* animals acclimated to 21% O_2. $n \geq 4$ assays for all genotypes. One-way ANOVA with Tukey test.

(K) An *arcp-1b* transgene rescues the enhanced CO_2 response of JU1249 animals acclimated to 21% O_2. n = 6 assays. One-way ANOVA with Tukey test.

For (H)–(K), each genotype was tested in at least 4 assays with 20–30 animals per trial. Solid lines plot mean; shaded areas show SEM; horizontal black bars indicate time intervals for statistical comparisons; vertical bars plot mean ± SEM. $^{**}p < 0.01$; $^{***}p < 0.001$; $^{****}p < 0.0001$; ns, not significant.

See also Figures S3, S4, and S5 and Data S1 and S2.

frameshift and premature stop codon in both transcripts of the *arcp-1* gene (Figures 2F and S5A). Second, we independently found several alleles of *arcp-1* in a collection of sequenced mutants that suppress aggregation behavior of *npr-1*(*null*) animals, including two that introduced premature stop codons. The number and kind of these alleles made it likely that disrupting *arcp-1* caused an aggregation defect. Consistent with this hypothesis, the aggregation defect of one strain (*db1082* allele) mapped to a 1 Mb interval on chromosome III, centered on *arcp-1* (Figures 2F and S5A). Two mutants from the million mutation project (Thompson et al., 2013), harboring *arcp-1* alleles (*gk856856* and *gk863317*) that introduce premature stop codons, were also defective in aggregation and bordering (Figures S5B and S5C). To show conclusively that mutations in *arcp-1* disrupt aggregation, we performed transgenic rescue experiments. Expressing wild-type *arcp-1* in JU1249 or in *arcp-1*(*db1082*)*; npr-1*(*null*) mutants restored aggregation and bordering behavior (Figure 2G).

To gain insight into the distribution of the *arcp-1*(*mfP22*) polymorphism in *C. elegans*, we examined other wild isolates. Our analysis suggests *mfP22* is a rare allele, because we did not find it in a set of 151 worldwide *C. elegans* isolates, including MY16 (Data S2).

Does disrupting *arcp-1* alter responses to CO_2? *arcp-1*(*db1082*)*; npr-1*(*null*) animals behaved like JU1249: they showed no overt defect in their response to a 21%-to-7% O_2 downshift (Figure S5D) but failed to suppress escape from different CO_2 concentrations when acclimated to 21% O_2 (Figures 2H, S5E, and S5F). A wild-type *arcp-1* transgene rescued this CO_2 plasticity defect (Figure 2I). *arcp-1* is thus required for animals acclimated to 21% O_2 to suppress escape from high CO_2 environments.

Gene predictions and cDNA cloning revealed *arcp-1a* and *arcp-1b* transcripts that overlap at their 3′ end (Figure 2F; Wormbase WS265). The *db1082* and *mfP22* alleles affect both *arcp-1* transcripts (Figure 2F). Expressing *arcp-1b* fully rescued the heightened CO_2 response of these animals, whereas a transgene for the longer *arcp-1a* transcript did not (Figures 2J and 2K). A mutation that only disrupted *arcp-1a* also failed to recapitulate the enhanced CO_2 response and aggregation phenotype of mutants defective in both *arcp-1* transcripts (Figures S5C and S5G). Thus *arcp-1*, and more specifically the product of its *b* transcript, is required for animals to suppress CO_2 escape following acclimation to 21% O_2.

ARCP-1 Acts in BAG Sensory Neurons to Suppress CO_2 Escape Behavior

arcp-1 encodes an ankyrin repeat protein (Figure 3A) homologous to *C. elegans* ankyrin UNC-44 and vertebrate ankyrins (Otsuka et al., 1995). These proteins are important for the subcellular localization of neural signaling complexes (e.g., anchoring components of the axon initial segment and allowing cyclic nucleotide-gated channels to accumulate in photoreceptor cilia) (Kizhatil et al., 2009; Leterrier et al., 2017; Maniar et al., 2011). Besides ankyrin repeats, ARCP-1 contains a DPY-30 domain (Figure 3A). Both domains are common protein interaction motifs that regulate the function and spatial organization of diverse signaling complexes (Gopal et al., 2012; Jones and Svitkina, 2016; Monteiro and Feng, 2017; Sivadas et al., 2012). ARCP-1's domain structure suggests it serves a similar role trafficking or localizing signaling proteins in the nervous system.

A fosmid-based bicistronic transgene that co-expressed *arcp-1* and free GFP was expressed in the main CO_2 and O_2 sensors: the URX, AQR, PQR, and BAG neurons (Figures 3B and 3C). We also observed expression in a subset of other sensory neurons (i.e., AFD, ASE, AWC, and AWB) (Figure 3C). This raised the possibility that disrupting *arcp-1* modifies plasticity in multiple paradigms. To test this, we assayed *arcp-1* mutants in a salt-based associative learning paradigm (Figure S6A; Beets et al., 2012; Hukema et al., 2008). *arcp-1* mutants were defective in gustatory plasticity: although mock-conditioned animals showed normal attraction to NaCl, upon salt conditioning they failed to downregulate salt chemotaxis behavior (Figure S6B).

To gain insight into *arcp-1* function, we focused on the failure of *arcp-1* mutants to suppress CO_2 escape when acclimated to 21% O_2. Because *arcp-1* is expressed in the BAG CO_2 sensors, we asked if it acts in these neurons to suppress CO_2 escape. Cell-specific expression of wild-type *arcp-1* in BAG using the *flp-17* promoter (Kim and Li, 2004) rescued the increased locomotory activity of *arcp-1* mutants at 3% CO_2 (Figure 3D). We also tested if *arcp-1* can act in URX, AQR, and PQR neurons, which sense 21% O_2, to suppress CO_2 escape. Expressing *arcp-1* in these neurons, using the *gcy-32* promoter (Yu et al., 1997), did not rescue the CO_2 phenotype of *arcp-1* mutants (Figure 3D). By contrast, the *arcp-1* aggregation defect could be rescued by expressing *arcp-1* either in BAG or in URX, AQR, and PQR (Figures S5H and S5I). Together, these data show that *arcp-1* functions in gas-sensing neurons and cell-autonomously suppresses CO_2 escape in the BAG CO_2 sensors.

BAG Responses to CO_2 Are Tuned by ARCP-1

We investigated if the increased behavioral response of *arcp-1* animals to CO_2 was associated with increased CO_2-evoked Ca^{2+} responses in BAG neurons. Using the ratiometric sensor YC3.60, we quantified fluorescence changes at the cell body of BAG in response to CO_2. Animals acclimated to 21% O_2 were transferred to a microfluidic chamber kept at 7% O_2 and stimulated with different CO_2 concentrations. BAG Ca^{2+} responses evoked by 1% and 3% CO_2 were significantly higher in *arcp-1* mutants compared to controls (Figure 4A). Unlike for CO_2 escape, expressing *arcp-1* either in BAG or in URX, AQR, and PQR rescued the CO_2 Ca^{2+} phenotype in BAG (Figure 4B). At 3% CO_2, animals with *arcp-1* rescued in URX, AQR, and PQR even showed a smaller increase in Ca^{2+} activity compared to *npr-1* controls, which may be due to an overexpression effect of the *gcy-32p::arcp-1b* transgene (Figure 4B). Because BAG neurons exhibit a phasic-tonic response to CO_2, we also measured Ca^{2+} responses during prolonged CO_2 stimulation. BAG tonic responses to 3% CO_2 were reduced in *arcp-1* mutants, although the effect was small (Figure S6C).

BAG neurons respond not only to a rise in CO_2, but also to a fall in O_2 (Zimmer et al., 2009). We asked if the CO_2 phenotypes of *arcp-1* animals could be indirectly linked to changes in BAG's ability to respond to O_2. BAG Ca^{2+} activity at 7% O_2, measured by YC2.60, was similar for *arcp-1* mutants and *npr-1* controls, although *arcp-1* animals displayed higher Ca^{2+} at

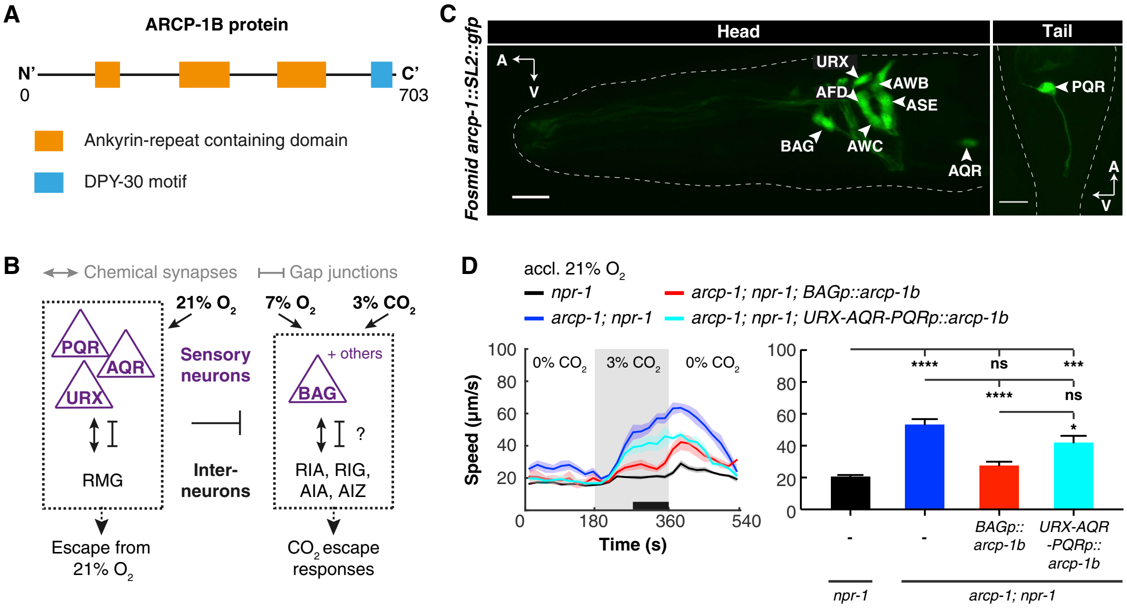

Figure 3. ARCP-1B Acts in BAG Sensors to Suppress CO_2 Escape Behavior

(A) Protein domain architecture of ARCP-1B.

(B) Schematic model of the core neural circuits for O_2 and CO_2 responses in *C. elegans* (Fenk and de Bono, 2017; Guillermin et al., 2017; Laurent et al., 2015). O_2-sensing neurons URX, AQR, and PQR tonically signal 21% O_2. CO_2 stimuli and O_2 downshifts are detected by BAG and other neurons. The O_2 sensors cross-modulate the neural circuit underlying CO_2 escape. The role of RIA, RIG, AIA, and AIZ in the CO_2 circuit is hypothesized based on their function in CO_2 aerotaxis (Guillermin et al., 2017).

(C) A fosmid reporter transgene for *arcp-1* is expressed in all major O_2 and CO_2 sensors, and other sensory neurons. Scale bar, 10 μm; A, anterior; V, ventral.

(D) Cell-specific expression of *arcp-1b* in BAG, using the *flp-17* promoter (*BAGp*), rescues locomotory arousal by CO_2, whereas expression in URX, AQR, and PQR, using the *gcy-32* promoter (*URX-AQR-PQRp*), does not. One-way ANOVA with Tukey test. $n \geq 5$ assays with 20–30 animals per trial. *$p < 0.05$; ***$p < 0.001$; ****$p < 0.0001$; ns, not significant.

See also Figures S5 and S6.

21% O_2 (Figure S6D). Ca^{2+} responses in URX to a 7% to 21% O_2 stimulus were unaffected in *arcp-1* animals (Figure S6E).

BAG O_2 responses are mediated by the guanylate cyclases GCY-31/GCY-33 and are abolished in mutants of these genes (Zimmer et al., 2009). Animals lacking *gcy-33* and *gcy-31*, like *arcp-1* mutants, were aroused more strongly by CO_2, but the effects on CO_2 escape were additive in an *arcp-1; gcy-33; gcy-31; npr-1* quadruple mutant (Figure S6F). Moreover, in *gcy-33; gcy-31* mutants, CO_2 arousal was suppressed when animals were acclimated to 21% O_2—unlike in *arcp-1* animals (Figure S6G). These results indicate that *arcp-1* can act in a separate genetic pathway from *gcy-33* and *gcy-31* to regulate CO_2 escape. Together with our rescue and Ca^{2+} imaging data, these findings are consistent with *arcp-1* suppressing CO_2 escape by inhibiting BAG responses to CO_2.

ARCP-1 Inhibits BAG-Mediated Turning Downstream of the CO_2 Receptor GCY-9

C. elegans respond to a rise in CO_2 not only by becoming aroused and moving faster but also by re-orienting their direction of travel and increasing the frequency of sharp (omega) turns. This behavior is also mediated by BAG (Fenk and de Bono, 2015; Hallem and Sternberg, 2008). Because ARCP-1 acts in BAG to suppress CO_2-evoked Ca^{2+} responses and locomotory arousal, we asked if it also inhibits CO_2-evoked turning. Both *arcp-1* mutants and JU1249 showed increased turning in response to a rise in CO_2 compared to controls (Figures 4C and 4D). This phenotype was rescued by expressing *arcp-1* in BAG, but not by expressing it in URX, AQR, and PQR (Figure 4D).

To gain insight into the molecular functions of *arcp-1*, we examined its effect on CO_2-evoked turns further. This part of the locomotory response to CO_2 is driven by cGMP signaling from the guanylyl cyclase receptor GCY-9 in BAG neurons (Fenk and de Bono, 2015; Hallem et al., 2011). GCY-9 is a molecular receptor for CO_2 and appears to be specifically expressed in BAG (Hallem et al., 2011; Smith et al., 2013). To examine if *arcp-1* regulates turning downstream of GCY-9, we measured CO_2-evoked turns in a *gcy-9; arcp-1* mutant. Disrupting *gcy-9* abolished turning evoked by 3% CO_2 in both *npr-1* and *arcp-1; npr-1* animals (Figure 4E), which implies that the mutant's turning phenotype depends on GCY-9, and ARCP-1 antagonizes GCY-9 signaling in BAG.

ARCP-1 Localizes Phosphodiesterase PDE-1 to BAG Cilia

The ankyrin repeats and DPY-30 motif of ARCP-1 suggest it serves as an interaction partner or scaffold for other proteins. To identify its molecular partners, we took a biochemical approach (Figure 5A). We first made a transgenic strain that

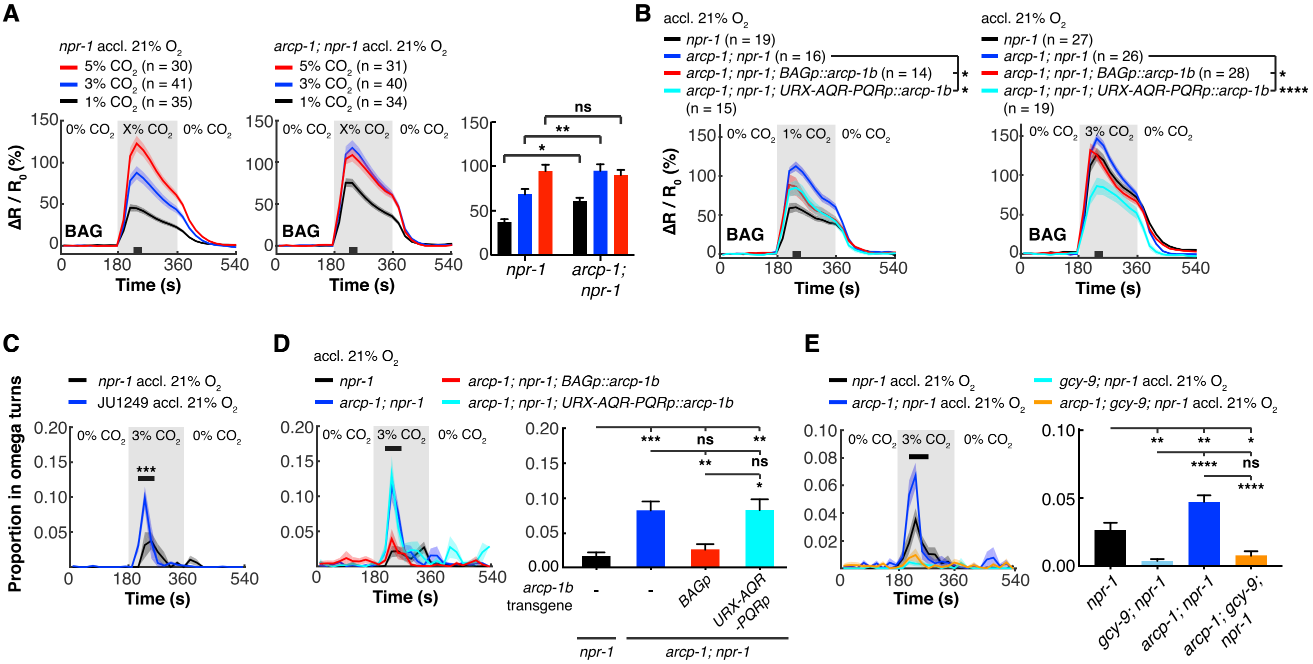

Figure 4. ARCP-1 Suppresses BAG Responses to CO_2

(A and B) Mean traces of BAG Ca^{2+} activity in *npr-1* and *arcp-1; npr-1* animals in response to different CO_2 concentrations. Mutants for *arcp-1* show increased Ca^{2+} activity at 1% and 3% CO_2 (A), which is rescued by expressing *arcp-1* either in BAG (*flp-17p*) or URX, AQR, and PQR (*gcy-32p*) (B). n = number of animals. Two-way ANOVA with Šidák test in (A). One-way ANOVA with Holm-Šidák test in (B).

(C–E) CO_2-evoked turning behavior. (C) Rising CO_2 levels stimulate stronger turning behavior in JU1249 (n = 85 animals) than in *npr-1(215F)* animals (n = 81). Mann-Whitney U test. (D) CO_2-evoked turning is also increased in *arcp-1(db1082); npr-1(ad609)* animals. BAG-specific expression of a *flp-17p::arcp-1b* transgene rescues this phenotype, whereas expression of *arcp-1b* in URX, AQR, and PQR (*gcy-32p*) does not. One-way ANOVA with Tukey test. n $\geq$ 5 assays with 20–30 animals per trial. (E) The increased turning of *arcp-1; npr-1* animals in response to CO_2 requires the GCY-9 CO_2 receptor. One-way ANOVA with Tukey test. n = 9 assays with 20–30 animals per trial.

For (A)–(E), solid lines plot mean; shaded areas show SEM; black bars indicate time intervals for statistical comparisons; bar graphs plot mean ± SEM for these intervals. *p < 0.05; **p < 0.01; ***p < 0.001; ****p < 0.0001; ns, not significant.

See also Figure S6.

expressed GFP-ARCP-1B and showed that it rescued the enhanced CO_2 response of the *arcp-1* mutant (Figure S7A). We then used anti-GFP nanobodies to pull down GFP-ARCP-1B fusion proteins from *C. elegans* lysates and identified putative interacting proteins by mass spectrometry (Figure 5A; Data S3). As a negative control, we immunoprecipitated other GFP-tagged cytoplasmic proteins in parallel. Across two independent experiments, we identified phosphodiesterase 1 (PDE-1) as the top specific hit (i.e., the protein having the highest number of spectral counts in ARCP-1B immunoprecipitates [IPs] while having none in control IPs) (Figure 5B; Data S3).

PDE-1 is a Ca^{2+}-activated cyclic guanosine monophosphate (cGMP)/cyclic AMP (cAMP) phosphodiesterase orthologous to mammalian Ca^{2+}/calmodulin-dependent PDE1 and is expressed in many neurons, including BAG (Couto et al., 2013; Hallem et al., 2011). As expected from our biochemical data, PDE-1 and ARCP-1 localize to similar compartments in BAG. GFP-tagged ARCP-1B was enriched at sensory endings (Figure 5C), similar to what we observe and what has been reported for PDE-1 (Martínez-Velázquez and Ringstad, 2018; Figure 5D).

The biochemical interaction of ARCP-1 and PDE-1, and their co-localization at dendritic endings, led us to hypothesize that ARCP-1 regulates PDE-1 localization. To test this, we compared enrichment of PDE-1 at BAG cilia in *arcp-1* and control animals. Overall, PDE-1 expression was slightly higher in *arcp-1* mutants, but enrichment of PDE-1 at the cilia was reduced by more than half in these animals (Figure 5E). To extend this observation, we investigated the subcellular localization of other signaling components of the gas-sensing neurons in *arcp-1* mutants. We observed a reduction of GCY-9 levels in BAG cilia, as well as reduced levels of the O_2-sensing guanylate cyclase GCY-35 at the sensory endings of URX (Figures S7B and S7C). These phenotypes were not due to a general defect in dendritic localization, because *arcp-1* mutants showed normal levels of the cGMP-gated channel subunit TAX-4 and the O_2-sensing guanylate cyclase GCY-33 in BAG cilia (Figures S7D and S7E). *arcp-1* mutants did not exhibit overt defects in dendritic morphology, based on expression of a *flp-17p::gfp* transgene and DiI filling of amphid sensory neurons (Figures S7F and S7G). Together, our data suggest that ARCP-1 acts as a scaffold that helps co-localize signal transduction components at sensory endings of some neurons.

Our behavioral, Ca^{2+} imaging and cell biological results led us to speculate that ARCP-1 promotes a Ca^{2+}-dependent

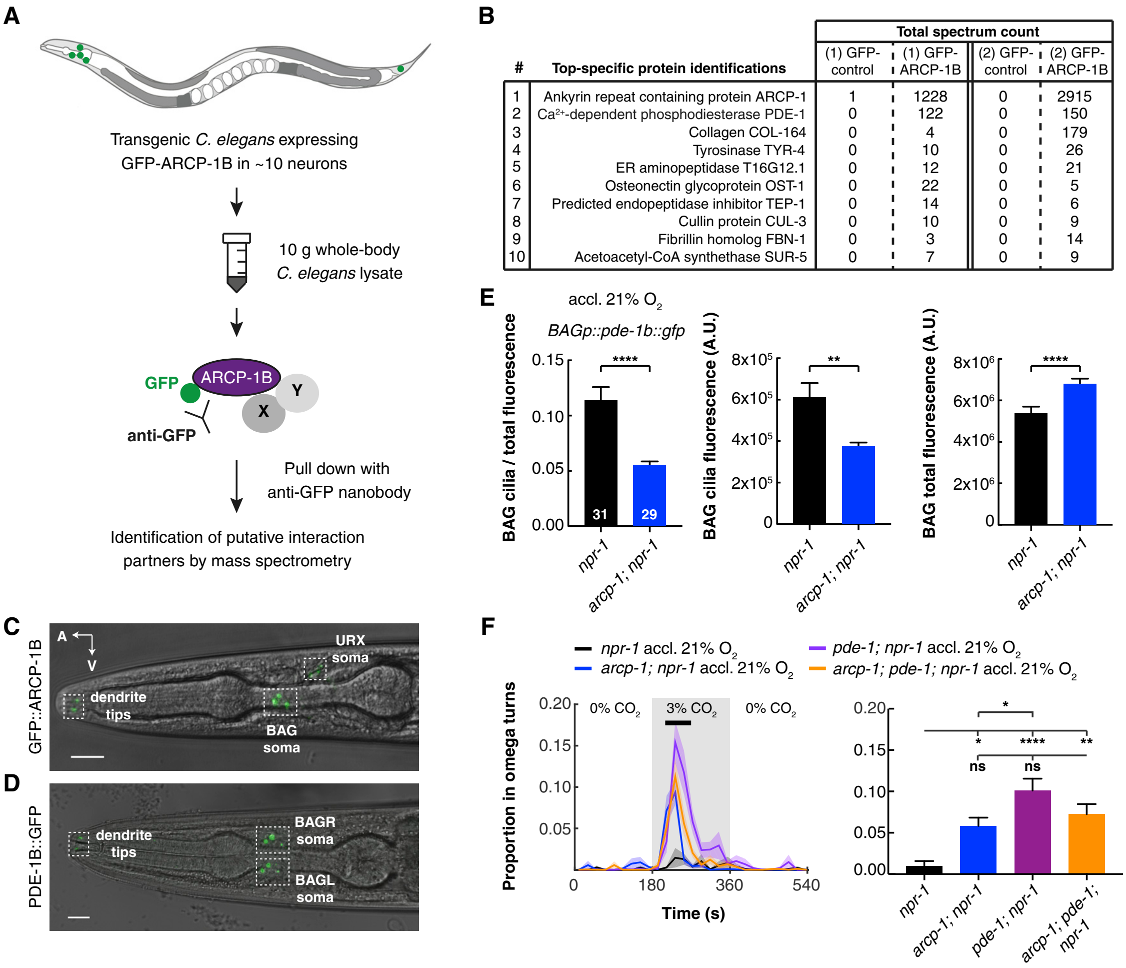

#	Top-specific protein identifications	Total spectrum count (1) GFP-control	(1) GFP-ARCP-1B	(2) GFP-control	(2) GFP-ARCP-1B
1	Ankyrin repeat containing protein ARCP-1	1	1228	0	2915
2	Ca^{2+}-dependent phosphodiesterase PDE-1	0	122	0	150
3	Collagen COL-164	0	4	0	179
4	Tyrosinase TYR-4	0	10	0	26
5	ER aminopeptidase T16G12.1	0	12	0	21
6	Osteonectin glycoprotein OST-1	0	22	0	5
7	Predicted endopeptidase inhibitor TEP-1	0	14	0	6
8	Cullin protein CUL-3	0	10	0	9
9	Fibrillin homolog FBN-1	0	3	0	14
10	Acetoacetyl-CoA synthethase SUR-5	0	7	0	9

Figure 5. ARCP-1 Is a Scaffolding Protein that Localizes Phosphodiesterase PDE-1 to Dendritic Endings

(A) Schematic of coimmunoprecipitation (coIP) approach to identify ARCP-1B interactors, by pull-down of an N-terminal GFP tag.

(B) Top ten specific putative interactors of GFP-ARCP-1B identified in two independent coIPs. IPs of other cytoplasmic GFP-tagged proteins provide negative controls.

(C and D) GFP-tagged ARCP-1B and PDE-1B proteins are both enriched at the sensory endings of BAG. Scale bar, 10 μm; A, anterior; V, ventral.

(E) Disrupting *arcp-1* reduces enrichment of PDE-1, expressed from the *flp-17p*, at BAG cilia. Bars plot mean ± SEM n (in bars) = number of animals. Mann-Whitney U test.

(F) *pde-1* mutants phenocopy the increased turning frequency of *arcp-1* mutants in response to CO_2. *pde-1; arcp-1* double mutants do not show an additive phenotype. Solid lines plot mean; shaded areas show SEM; black bars indicate time intervals for statistical comparisons; bar graphs plot mean ± SEM for these intervals. One-way ANOVA with Tukey test. n ≥ 8 assays with 20–30 animals per trial. *$p < 0.05$; **$p < 0.01$; ****$p < 0.0001$; ns, not significant.

See also Figure S7 and Data S3.

feedback mechanism mediated by PDE-1, which keeps BAG CO_2 responses in check by degrading cGMP following activation of the CO_2 receptor GCY-9. If this is correct, disrupting *pde-1* should phenocopy *arcp-1* and increase the frequency of CO_2-evoked turns. Moreover, the *arcp-1* and *pde-1* phenotypes should not be additive. As predicted, *pde-1* mutants turned more in response to 3% CO_2 than controls and even *arcp-1* mutants, likely because *pde-1* is more widely expressed and serves broader functions than *arcp-1*. The turning phenotype was comparable for *pde-1*, *arcp-1*, and *pde-1; arcp-1* mutants (Figure 5F). These results are consistent with *pde-1* and *arcp-1* acting in the same genetic pathway to keep CO_2 responses in check.

PDE-1 and ARCP-1 Inhibit Expression of FLP-19 Neuropeptides

To investigate further how disrupting *arcp-1* alters BAG function, we specifically labeled these neurons with GFP, used fluorescence-activated cell sorting (FACS) to isolate the fluorescent cells from acutely dissociated *arcp-1;npr-1* and *npr-1* control animals, and profiled their gene expression using RNA sequencing (RNA-seq) (see STAR Methods). Genes that are hallmarks of BAG, such as those involved in CO_2 signaling (*gcy-9*, *pde-1*, *flp-17*) and BAG cell fate determination (*ets-5*) (Guillermin et al., 2011; Hallem et al., 2011), were among the top enriched genes in our dataset (Data S4).

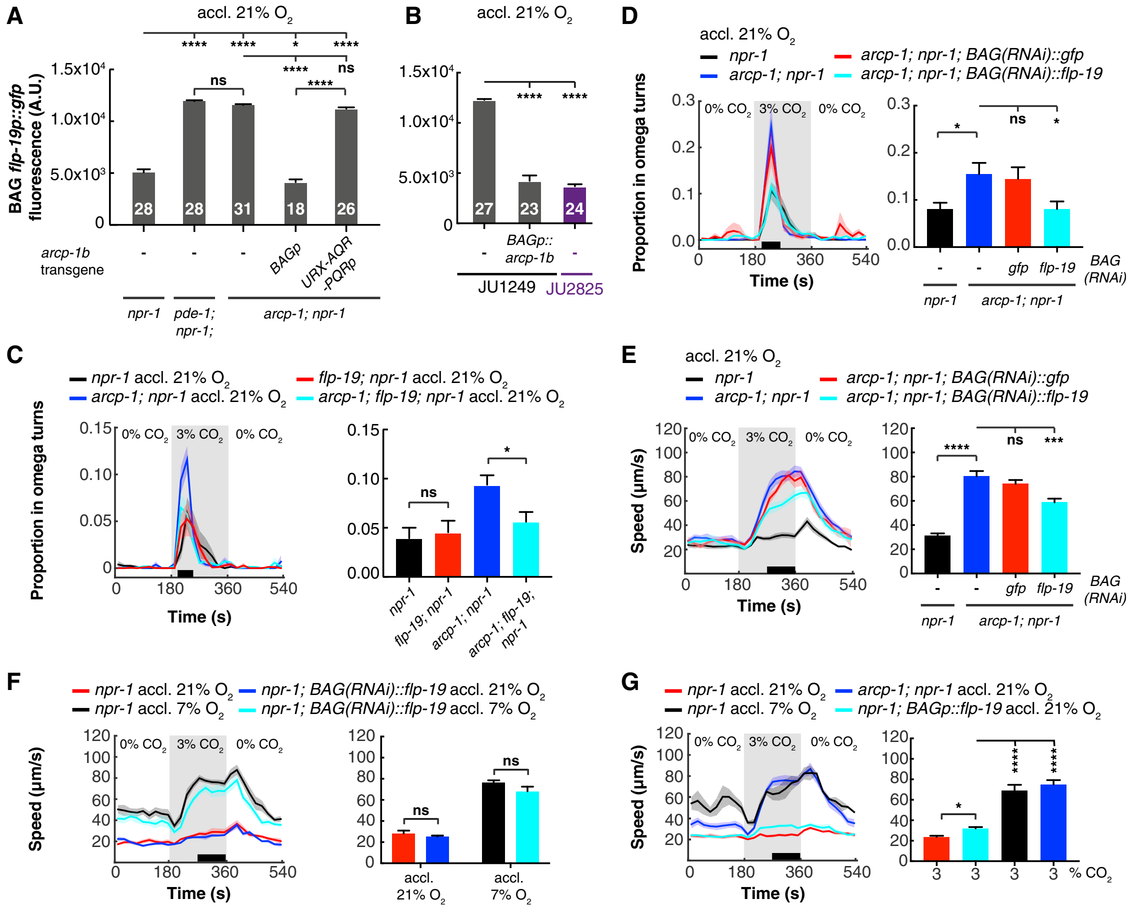

Figure 6. PDE-1 and ARCP-1 Inhibit BAG Expression of FLP-19 Neuropeptides that Potentiate Behavioral Responses to CO_2

(A) Mean fluorescence ± SEM of a *flp-19* neuropeptide reporter (*flp-19p::gfp*) in BAG, indicating that PDE-1 and ARCP-1 inhibit *flp-19* expression. BAG-specific expression of *arcp-1b*, using the *flp-17* promoter (*BAGp*), rescues this phenotype, whereas expression in URX, AQR and PQR, using the *gcy-32* promoter (*URX-AQR-PQRp*), does not. n (in bars) = number of animals. One-way ANOVA with Tukey test.

(B) Mean fluorescence ± SEM of *flp-19* neuropeptide reporter in BAG neurons of JU1249 and JU2825. Increased expression of *flp-19* in JU1249 is rescued by expressing *arcp-1b* from the BAG-specific *flp-17* promoter (*BAGp*). n (in bars) = number of animals. Kruskal-Wallis with Dunn test.

(C) Disrupting *flp-19* suppresses the potentiated turning phenotype of *arcp-1; npr-1* animals in response to 3% CO_2. One-way ANOVA with Holm Šidák test. n = 9 assays.

(D) CO_2-evoked turning of *arcp-1; npr-1* mutants following cell-specific knock down of *flp-19* expression in BAG. Knock down of *flp-19* in the mutant background suppresses turning at 3% CO_2, whereas knock down of *gfp* does not. One-way ANOVA with Dunnett's test. n $\geq$ 7 assays.

(E) Knock down of *flp-19* expression in BAG partially rescues the increased arousal phenotype of *arcp-1; npr-1* animals at 3% CO_2. One-way ANOVA with Dunnett's test. n $\geq$ 7 assays with 20–30 animals per trial.

(F) BAG-specific knock down of *flp-19* in *npr-1* animals does not affect the plasticity of CO_2 escape in response to previous O_2 experience. Two-way ANOVA with Šidák test. n = 7–8 assays.

(G) Animals overexpressing *flp-19* in BAG move significantly faster at 3% CO_2 compared to *npr-1* controls, although their response is still lower than *npr-1* animals grown at 7% O_2 and *arcp-1* mutants. n $\geq$ 3 assays. One-way ANOVA with Tukey test.

For (C)–(G), 20–30 animals were tested per assay. Solid lines plot mean; shaded areas show SEM; black bars indicate time intervals for statistical comparisons; bars plot mean ± SEM for these intervals. *p < 0.05; ***p < 0.001; ****p < 0.0001; ns, not significant.

See also Figure S7 and Data S4.

arcp-1 itself was among the 100 most highly expressed genes in BAG. The BAG profiles highlighted significant gene expression differences between *arcp-1* mutants and controls, notably changes in the abundance of mRNAs encoding neuropeptides, genes involved in ciliary intraflagellar transport, ion channels, and gap junction subunits (see Discussion; Data S4D). These data suggest that loss of ARCP-1 leads to altered gene expression.

One of the most abundant transcripts expressed in BAG whose expression was significantly altered by defects in *arcp-1* was the neuropeptide *flp-19*. *flp-19* expression was upregulated 2.4-fold in *arcp-1* animals, which would be consistent with increased BAG signaling. Previous work has shown that GCY-9, PDE-1, and the cGMP-gated Ca^{2+} channel TAX-4 control *flp-19* expression in BAG (Romanos et al., 2017), making it an interesting candidate for altering CO_2 responses in the

arcp-1 mutant. To confirm that defects in *arcp-1* increased expression of *flp-19*, we introduced a *flp-19p::gfp* reporter transgene (Kim and Li, 2004) into *arcp-1* mutants and quantified fluorescence in BAG neurons. Disrupting *arcp-1* significantly increased BAG expression of the neuropeptide reporter (Figure 6A). This phenotype was rescued by expressing wild-type *arcp-1* in BAG, but not in the O_2 sensors URX, AQR, and PQR (Figure 6A). Thus, *arcp-1* controls *flp-19* expression cell-autonomously in BAG. We observed a similar increase in expression of the *flp-19* reporter when *pde-1* was mutated (Figure 6A). BAG expression of *flp-19* in mutants lacking both *arcp-1* and *pde-1* was similar to that of the single mutants (Figure S7H). These data suggest that ARCP-1 and PDE-1 together reduce BAG signaling by lowering the expression of some neuropeptides. However, disrupting *arcp-1* does not generally increase BAG neuropeptide expression as judged from our BAG profiling experiments (Data S4) and analysis of a *flp-17* neuropeptide reporter in BAG (Figure S7I).

To ask if *flp-19* expression was elevated in JU1249, we backcrossed the *flp-19p::gfp* transgene ten times to this isolate. We did the same for JU2825 that, unlike JU1249, suppressed CO_2 escape when acclimated to 21% O_2 (Figure S2A). BAG expression of *flp-19* was low in JU2825 and high in JU1249 (Figure 6B). Restoring *arcp-1* in BAG significantly reduced *flp-19* expression (Figure 6B). Thus, disrupting *arcp-1* also increases *flp-19* expression in JU1249.

FLP-19 Neuropeptide Signaling from BAG Potentiates Behavioral Responses to CO_2

Does increased BAG expression of *flp-19* in *arcp-1* mutants enhance the behavioral responses of these animals to CO_2? If increased *flp-19* expression heightened aversion to CO_2 in *arcp-1* animals, then disrupting *flp-19* should reverse this phenotype. Consistent with this hypothesis, deleting *flp-19* restored turning at 3% CO_2 in the *arcp-1* mutant, while it had no effect on this behavior in *npr-1* animals (Figure 6C).

To confirm that FLP-19 release from BAG potentiates CO_2 responses, we knocked down *flp-19* expression specifically in these neurons by expressing RNAi sense and antisense sequences of *flp-19* from a BAG-specific *gcy-33* promoter (Hallem et al., 2011; Yu et al., 1997). As a negative control, we expressed sense and antisense sequences for *gfp* under the same promoter and found it had no effect on CO_2 responses (Figures 6D and 6E). By contrast, BAG-specific knockdown of *flp-19* in *arcp-1* mutants restored the frequency of CO_2-evoked turns (Figure 6D) and reduced CO_2-evoked locomotory arousal in animals acclimated to 21% O_2 (Figure 6E). These data suggest increased *flp-19* expression in BAG contributes to the enhanced behavioral responses to CO_2 in *arcp-1* mutants.

The neuropeptide gene *flp-19* is also expressed in URX. However, knock down of *flp-19* in these neurons, using the *gcy-32* promoter, enhanced rather than reduced locomotory arousal at 3% CO_2 in *arcp-1* animals and increased baseline locomotion in the absence of CO_2 (Figure S7K). This result is consistent with previous reports (Carrillo et al., 2013) and suggests that the RNAi effect in BAG is specific to these neurons. We wondered if altered expression of *flp-19* from URX contributes to the enhanced CO_2 aversion in *arcp-1* animals as well. If this is the case, *flp-19* expression in URX should be reduced in *arcp-1* mutants. Indeed, disrupting *arcp-1* decreased expression of the *flp-19* reporter in URX. This phenotype was rescued by expressing *arcp-1* either in URX or BAG neurons (Figure S7L), suggesting that BAG signaling indirectly influences *flp-19* expression in URX.

Does FLP-19 release from BAG promote escape from CO_2 in animals that retain functional *arcp-1*? In *npr-1* animals, BAG-specific knock down of *flp-19* did not compromise CO_2 escape in animals acclimated to 21% O_2 or 7% O_2 (Figure 6F). Thus, *flp-19* is not required for the O_2-dependent modulation of CO_2 responses. Consistent with this finding, we observed similar expression of the *flp-19* reporter in *npr-1* animals acclimated to 21% or 7% O_2, suggesting that *flp-19* expression is not regulated by O_2 experience (Figure S7J).

We next asked if increased *flp-19* expression in BAG is sufficient to boost *C. elegans*' locomotory arousal by CO_2. To test this, we overexpressed *flp-19* specifically in the BAG neurons of *npr-1* animals, acclimated these transgenic animals to 21% O_2, and quantified their speed at 3% CO_2. Animals overexpressing *flp-19* in BAG moved significantly faster at 3% CO_2 than *npr-1* controls, although their locomotory arousal was weaker than that of *arcp-1* animals or of *npr-1* animals grown at 7% O_2 (Figure 6G). Thus, acclimation to 7% O_2 or disrupting *arcp-1* alters other signals besides *flp-19* to heighten CO_2 responses. However, in both scenarios—disruption of *arcp-1* or O_2 acclimation—increasing *flp-19* expression in BAG can potentiate behavioral responses to CO_2, leading to increased CO_2 aversion.

DISCUSSION

Individuals differ in how they respond to altered circumstances in their environment. This is generally ascribed to a combination of genetic variation and different life experiences. How neural circuits encoding behavioral plasticity vary across individuals is, however, poorly understood. Here, we show that *Caenorhabditis* species and wild isolates of *C. elegans* can differ in how past O_2 experience influences CO_2 escape behavior. We uncover a genetic variant and neuronal mechanism responsible for this variation in behavioral flexibility in one natural *C. elegans* isolate.

The behavioral phenotypes that we observe are reminiscent of genetic accommodation, when the reaction norm of a flexible phenotype responsive to the environment is altered by genetic change (Figure 7A). Underlying this behavioral change, we find that disrupting ARCP-1 both increases CO_2 sensitivity and alters the effect of previous O_2 experience on CO_2 escape. Animals lacking this dendritic scaffold protein become strongly aroused by CO_2 regardless of previous O_2 experience, and acclimation to 21% O_2 further enhances, rather than suppresses, escape from this aversive cue. We show that loss of *arcp-1* mediates these phenotypes by directly altering CO_2 responses, rather than by affecting the ability to respond to O_2.

We identify the BAG CO_2 sensors as the main site where ARCP-1 suppresses CO_2 escape in animals acclimated to 21% O_2. Together with previous work (Couto et al., 2013; Romanos et al., 2017), our results suggest a model (Figure 7B) in which

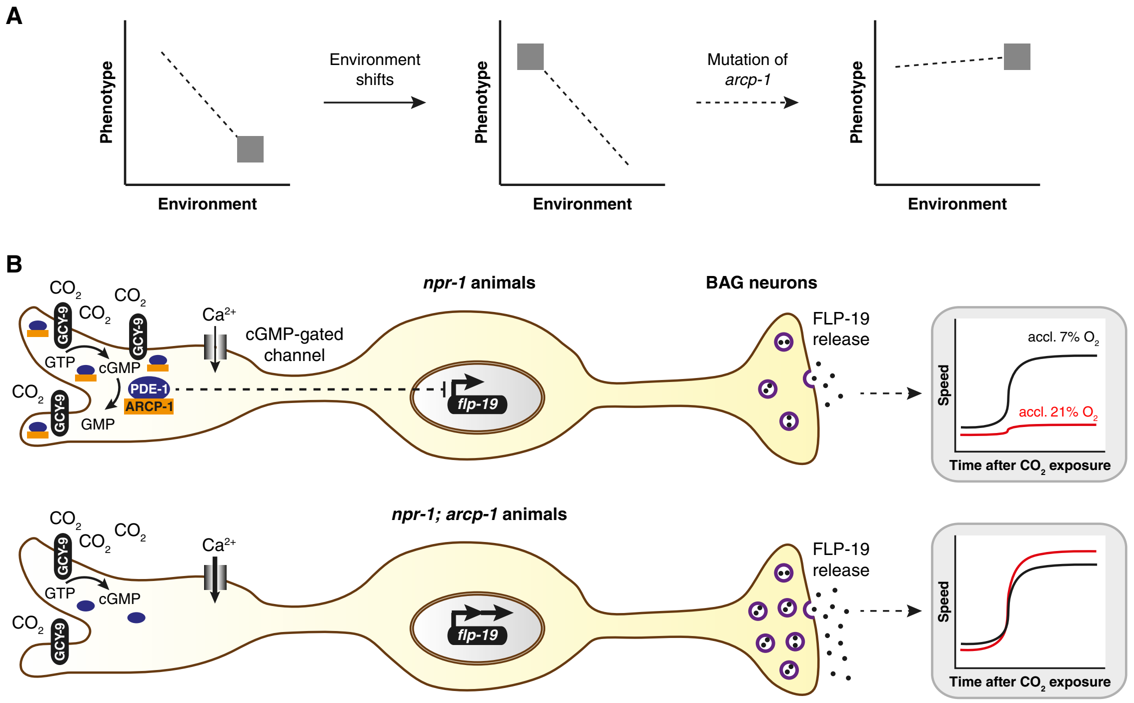

Figure 7. A Model for How Genetic Variation in *arcp-1* Affects CO_2 Escape Behavior

(A) Effect of the natural *arcp-1* allele on experience-dependent plasticity, shown as behavioral reaction norms. *C. elegans* wild isolates acclimated to a high (21%) O_2 environment suppress their aversion to CO_2 (left panel). A shift to a low (7%) O_2 environment results in a heightened CO_2 response. A mutation in *arcp-1* alters experience-dependent plasticity and genetically fixes a strong aversive response to CO_2 in part by increasing *flp-19* neuropeptide expression in BAG CO_2 sensors (right panel).

(B) CO_2 is detected by the receptor guanylate cyclase GCY-9, expressed in BAG cilia. The ankyrin-repeat scaffold protein ARCP-1 is also enriched at dendritic sensory endings, interacts with PDE-1, and localizes this phosphodiesterase to the cilia of BAG CO_2-sensory neurons. PDE-1 and ARCP-1 inhibit CO_2-evoked Ca^{2+} activity and expression of FLP-19 neuropeptide messengers in BAG. In the absence of ARCP-1, less GCY-9 and PDE-1 localize to BAG cilia, and *flp-19* is more strongly expressed. Increased FLP-19 expression in BAG contributes to resetting a strong aversive response to CO_2 in *arcp-1; npr-1* animals regardless of previous O_2 experience.

ARCP-1 binds and co-localizes the Ca^{2+}-activated phosphodiesterase PDE-1 with guanylyl cyclase receptors for CO_2 at the BAG cilia. ARCP-1 and PDE-1 keep signaling from these neurons in check by suppressing CO_2-evoked Ca^{2+} responses and neuropeptide expression. Natural genetic variation has been found to directly alter sensory systems in other animals (McGrath, 2013; Prieto-Godino et al., 2017). We identify BAG as a major cellular focus for variation in CO_2 responses, but the possibility remains that loss of *arcp-1* disrupts plasticity in other sensory circuits, which may indirectly promote CO_2 aversion as well. Some evidence points to changes in URX, but these are not sufficient to explain the heightened CO_2 escape behavior in *arcp-1* mutants.

Mounting evidence suggests that natural variation in behavioral flexibility is genetically determined (Izquierdo et al., 2007; Mery, 2013; Mery et al., 2007). One well-established example is the natural variation seen at the *foraging* gene in *Drosophila melanogaster*. This polymorphism causes individual variation in learning and memory, among other phenotypes, by altering the activity of cGMP-dependent protein kinase G (Mery et al., 2007). It is notable that, both in flies and worms, genetic variation affecting cGMP signaling underlies inter-individual variation in experience-dependent plasticity. Besides gas sensors, ARCP-1 is expressed in olfactory, gustatory, and thermosensory neurons that all signal using cGMP, and *arcp-1* mutants show reduced plasticity in a gustatory paradigm. Correlated differences in the plasticity of different sensory modalities have been described as coping styles or behavioral syndromes in other animal models (Coppens et al., 2010) and may also reflect a common genetic or molecular basis. Identifying how loss of *arcp-1* compromises plasticity in other sensory circuits should provide a better understanding of such correlated changes in behavioral flexibility.

We have shown that the absence of ARCP-1 alters expression of a range of genes in BAG. One way this influences CO_2 aversion is by altering the expression of neuropeptide messengers. Neuropeptides are a diverse group of neuromodulators that, both in vertebrates and invertebrates, are involved in circuit plasticity (Jékely et al., 2018; Taghert and Nitabach, 2012). Natural genetic variation in neuropeptide pathways has been linked to individual differences in aging and social behaviors (Donaldson and Young, 2008; Yin et al., 2017). Our results suggest that they also contribute to heritable differences in behavioral plasticity between individuals. In humans and other primates, natural polymorphisms in serotonergic and dopaminergic systems have been associated with individual differences in

memory and cognitive ability (Izquierdo et al., 2007; Zhang et al., 2007). Changing the neuromodulatory tone of circuits likely represents a general mechanism by which genetic variation sculpts individual behavioral plasticity.

Disrupting ARCP-1 increases expression of FLP-19 neuropeptides in BAG. This potentiates or disinhibits both CO_2-evoked turning and locomotory arousal in animals acclimated to 21% O_2. A FLP-19 receptor is currently unknown; the *C. elegans* genome encodes ~150 predicted neuropeptide receptors but none have been reported to bind FLP-19 (Peymen et al., 2014). FLP-19 neuropeptides belong to the ancient and conserved family of RFamide neuropeptides (Peymen et al., 2014). Previous work suggested that CO_2-evoked cGMP and Ca^{2+} signaling promote *flp-19* expression in BAG, and this effect is counterbalanced by PDE-1 (Romanos et al., 2017). In *arcp-1* mutants, the GCY-9 CO_2 receptor and PDE-1 are less enriched at BAG cilia. Although *gcy-9* expression is slightly reduced, disrupting ARCP-1 increases BAG Ca^{2+} activity in response to CO_2. This is consistent with proper ciliary localization of PDE-1 keeping BAG Ca^{2+} signaling in check and could explain the increased *flp-19* expression. ARCP-1 and PDE-1 may also orchestrate microdomains of cGMP that can regulate gene expression (Arora et al., 2013; O'Halloran et al., 2012). In vertebrate neurons, nanodomains of the ankyrin G protein, a homolog of ARCP-1, localize to the dendritic spines and the axon initial segment and contribute to neural plasticity (Grubb and Burrone, 2010; Smith et al., 2014). Likewise, mammalian PDE1 has been implicated in the experience-dependent adaptation of sensory responses. In mouse olfactory neurons, PDE1 is specifically enriched at the cilia, although a molecular anchor that localizes the protein to this compartment has not yet been identified (Cygnar and Zhao, 2009).

The molecular mechanism by which ARCP-1 controls *flp-19* expression, and whether this relates to its ciliary function, remains to be understood. Interestingly, our transcriptional profiling of BAG neurons revealed a suite of genes involved in intraflagellar transport, including the axonemal dynein *che-3*, that show ~2-fold increased expression in *arcp-1* animals, although we did not observe obvious defects in cilia morphology. This suggests a feedback mechanism exists by which signaling at the cilium regulates expression of genes involved in ciliary transport. Identifying the molecular factors involved is the next step forward toward understanding this transcriptional regulation.

The mechanisms through which natural genetic variation in *arcp-1*, acting on an evolutionary timescale, and O_2 experience, acting over an animal's lifetime, sculpt CO_2 responsiveness seem to be at least partly distinct. However, in both scenarios—disruption of *arcp-1* or acclimation to different O_2 environments—release of FLP-19 neuropeptides from BAG can boost the animal's response to this aversive cue, and through alterations in neuropeptide expression, a strong aversive response may become fixed.

CO_2 responses vary between *Caenorhabditis* species (Carrillo and Hallem, 2015; Pline and Dusenbery, 1987; Viglierchio, 1990). Our results show this variation is at least in part due to differences in O_2-dependent modulation, suggesting it is an adaptive trait. We speculate that the influence of O_2 memory on other sensory responses enables animals to reconfigure their behavioral priorities according to past experience (Fenk and de Bono, 2017). Animals at the surface may prioritize escape from 21% O_2 and gradually suppress their CO_2 aversion to facilitate migration to buried environments with less aeration and elevated CO_2 levels. Natural variation in the O_2-dependent modulation of CO_2 escape may result in animals occupying different ecological niches. Alternatively, there could be selection against the costs to maintain sensory systems for behavioral plasticity (Dewitt et al., 1998), which may account for the reduced plasticity of CO_2 responses in some nematode species. We do not know the O_2 and CO_2 conditions in which the *arcp-1* deletion may have been selected and can therefore only speculate about its potential fitness benefits. The *arcp-1* mutation was not found in any other wild isolate so is likely recent. However, our data indicate substantial variation among both *Caenorhabditis* species and *C. elegans* isolates in the response to CO_2 (Figures 1 and S2). These findings are consistent with what has been found for other traits (Frézal et al., 2018), where the phenotypic variation for the strain chosen for study is caused by a rare allele found only in that strain, yet phenotypic variation itself is not restricted to that strain. Understanding evolutionary mechanisms that might select for altered plasticity requires more in-depth knowledge on the ecology of these species. The behavioral phenotypes that we observe are consistent with genetic accommodation for a cross-modal gene-environment interaction (Pigliucci et al., 2006). In summary, our study illustrates how natural genetic variation, by altering the neuromodulatory control of aversive behavior, contributes to individual differences in behavioral flexibility.

STAR★METHODS

Detailed methods are provided in the online version of this paper and include the following:

- KEY RESOURCES TABLE
- LEAD CONTACT AND MATERIALS AVAILABILITY
- EXPERIMENTAL MODEL AND SUBJECT DETAILS
  - Animals
  - Microbe strains
- METHOD DETAILS
  - Molecular biology
  - Genotyping of natural polymorphisms
  - Behavioral assays
  - Selection-based QTL mapping
  - Distribution of *mfP22* allele in wild isolates
  - Confocal microscopy and image analysis
  - Calcium imaging
  - Immunoprecipitation from *C. elegans*
  - RNA-seq of sorted BAG neurons
- QUANTIFICATION AND STATISTICAL ANALYSIS
- DATA AND SOFTWARE AVAILABILITY
  - Datasets
  - Codes

SUPPLEMENTAL INFORMATION

Supplemental Information can be found online at https://doi.org/10.1016/j.neuron.2019.10.001.

ACKNOWLEDGMENTS

We thank Roger Pocock (Monash University, Australia), Niels Ringstad (New York University School of Medicine, USA), and the *Caenorhabditis* Genetics Center, funded by NIH Office of Research Infrastructure Programs (P40 OD010440), for strains. We gratefully acknowledge Tim Stevens, Gurpreet Ghattaoraya, and members of the de Bono and Schafer labs for advice and comments. This work was funded by an Advanced Grant from the European Research Council to M.d.B (ACMO 269058) and by the Medical Research Council (United Kingdom). I.B. received an EMBO Long-term Fellowship (ALTF 387-2015) and EU Marie-Curie Individual Fellowship (MSCA-IF 704383) and is a fellow of the Research Foundation—Flanders (FWO). Work in the Félix lab was funded by a grant from the Fondation pour la Recherche Médicale (DEQ20150331704). G.Z. was funded in part by a fellowship from the China Scholarship Council.

AUTHOR CONTRIBUTIONS

I.B., G.Z., M.-A.F., and M.d.B. designed experiments. I.B., G.Z., L.A.F., and C.C. performed experiments. I.B., G.Z., M.-A.F., and M.d.B. analyzed the data. G.Z. and G.N. did the genome sequence data analyses. I.B., M.-A.F., and M.d.B. wrote the manuscript.

DECLARATION OF INTERESTS

The authors declare no competing interests.

Received: January 26, 2019
Revised: August 18, 2019
Accepted: September 28, 2019
Published: November 19, 2019

SUPPORTING CITATIONS

The following references appear in the Supplemental Information: de Bono and Bargmann (1998); Félix and Duveau (2012); Kiontke et al. (2011); Richaud et al. (2018).

REFERENCES

Arora, K., Sinha, C., Zhang, W., Ren, A., Moon, C.S., Yarlagadda, S., and Naren, A.P. (2013). Compartmentalization of cyclic nucleotide signaling: a question of when, where, and why? Pflugers Arch. *465*, 1397–1407.

Bargmann, C.I. (2012). Beyond the connectome: how neuromodulators shape neural circuits. BioEssays *34*, 458–465.

Beets, I., Janssen, T., Meelkop, E., Temmerman, L., Suetens, N., Rademakers, S., Jansen, G., and Schoofs, L. (2012). Vasopressin/oxytocin-related signaling regulates gustatory associative learning in *C. elegans*. Science *338*, 543–545.

Bretscher, A.J., Kodama-Namba, E., Busch, K.E., Murphy, R.J., Soltesz, Z., Laurent, P., and de Bono, M. (2011). Temperature, oxygen, and salt-sensing neurons in *C. elegans* are carbon dioxide sensors that control avoidance behavior. Neuron *69*, 1099–1113.

Busch, K.E., Laurent, P., Soltesz, Z., Murphy, R.J., Faivre, O., Hedwig, B., Thomas, M., Smith, H.L., and de Bono, M. (2012). Tonic signaling from O_2 sensors sets neural circuit activity and behavioral state. Nat. Neurosci. *15*, 581–591.

Carrillo, M.A., and Hallem, E.A. (2015). Gas sensing in nematodes. Mol. Neurobiol. *51*, 919–931.

Carrillo, M.A., Guillermin, M.L., Rengarajan, S., Okubo, R.P., and Hallem, E.A. (2013). O_2-sensing neurons control CO_2 response in *C. elegans*. J. Neurosci. *33*, 9675–9683.

Cook, D.E., Zdraljevic, S., Roberts, J.P., and Andersen, E.C. (2016). CeNDR, the *Caenorhabditis elegans* natural diversity resource. Nucleic Acids Res. *45* (D1), D650–D657.

Coppens, C.M., de Boer, S.F., and Koolhaas, J.M. (2010). Coping styles and behavioural flexibility: towards underlying mechanisms. Philos. Trans. R. Soc. Lond. B Biol. Sci. *365*, 4021–4028.

Couto, A., Oda, S., Nikolaev, V.O., Soltesz, Z., and de Bono, M. (2013). In vivo genetic dissection of O_2-evoked cGMP dynamics in a *Caenorhabditis elegans* gas sensor. Proc. Natl. Acad. Sci. USA *110*, E3301–E3310.

Crispo, E. (2007). The Baldwin effect and genetic assimilation: revisiting two mechanisms of evolutionary change mediated by phenotypic plasticity. Evolution *61*, 2469–2479.

Cummins, E.P., Selfridge, A.C., Sporn, P.H., Sznajder, J.I., and Taylor, C.T. (2014). Carbon dioxide-sensing in organisms and its implications for human disease. Cell. Mol. Life Sci. *71*, 831–845.

Cygnar, K.D., and Zhao, H. (2009). Phosphodiesterase 1C is dispensable for rapid response termination of olfactory sensory neurons. Nat. Neurosci. *12*, 454–462.

de Bono, M., and Bargmann, C.I. (1998). Natural variation in a neuropeptide Y receptor homolog modifies social behavior and food response in *C. elegans*. Cell *94*, 679–689.

Dewitt, T.J., Sih, A., and Wilson, D.S. (1998). Costs and limits of phenotypic plasticity. Trends Ecol. Evol. *13*, 77–81.

Dingemanse, N.J., and Wolf, M. (2013). Between-individual differences in behavioural plasticity within populations: causes and consequences. Anim. Behav. *85*, 1031–1039.

Donaldson, Z.R., and Young, L.J. (2008). Oxytocin, vasopressin, and the neurogenetics of sociality. Science *322*, 900–904.

Duveau, F., and Félix, M.A. (2012). Role of pleiotropy in the evolution of a cryptic developmental variation in *Caenorhabditis elegans*. PLoS Biol. *10*, e1001230.

Félix, M.A., and Duveau, F. (2012). Population dynamics and habitat sharing of natural populations of *Caenorhabditis elegans* and *C. briggsae*. BMC Biol. *10*, 59.

Fenk, L.A., and de Bono, M. (2015). Environmental CO_2 inhibits *Caenorhabditis elegans* egg-laying by modulating olfactory neurons and evokes widespread changes in neural activity. Proc. Natl. Acad. Sci. USA *112*, E3525–E3534.

Fenk, L.A., and de Bono, M. (2017). Memory of recent oxygen experience switches pheromone valence in *Caenorhabditis elegans*. Proc. Natl. Acad. Sci. USA *114*, 4195–4200.

Frézal, L., Demoinet, E., Braendle, C., Miska, E., and Félix, M.A. (2018). Natural genetic variation in a multigenerational phenotype in *C. elegans*. Curr. Biol. *28*, 2588–2596.

Gopal, R., Foster, K.W., and Yang, P. (2012). The DPY-30 domain and its flanking sequence mediate the assembly and modulation of flagellar radial spoke complexes. Mol. Cell. Biol. *32*, 4012–4024.

Gray, J.M., Karow, D.S., Lu, H., Chang, A.J., Chang, J.S., Ellis, R.E., Marletta, M.A., and Bargmann, C.I. (2004). Oxygen sensation and social feeding mediated by a *C. elegans* guanylate cyclase homologue. Nature *430*, 317–322.

Gross, E., Soltesz, Z., Oda, S., Zelmanovich, V., Abergel, Z., and de Bono, M. (2014). GLOBIN-5-dependent O_2 responses are regulated by PDL-1/PrBP that targets prenylated soluble guanylate cyclases to dendritic endings. J. Neurosci. *34*, 16726–16738.

Grubb, M.S., and Burrone, J. (2010). Activity-dependent relocation of the axon initial segment fine-tunes neuronal excitability. Nature *465*, 1070–1074.

Guerenstein, P.G., and Hildebrand, J.G. (2008). Roles and effects of environmental carbon dioxide in insect life. Annu. Rev. Entomol. *53*, 161–178.

Guillermin, M.L., Castelletto, M.L., and Hallem, E.A. (2011). Differentiation of carbon dioxide-sensing neurons in *Caenorhabditis elegans* requires the ETS-5 transcription factor. Genetics *189*, 1327–1339.

Guillermin, M.L., Carrillo, M.A., and Hallem, E.A. (2017). A single set of interneurons drives opposite behaviors in *C. elegans*. Curr. Biol. *27*, 2630–2639.

Hallem, E.A., and Sternberg, P.W. (2008). Acute carbon dioxide avoidance in *Caenorhabditis elegans*. Proc. Natl. Acad. Sci. USA *105*, 8038–8043.

Hallem, E.A., Spencer, W.C., McWhirter, R.D., Zeller, G., Henz, S.R., Rätsch, G., Miller, D.M., 3rd, Horvitz, H.R., Sternberg, P.W., and Ringstad, N. (2011). Receptor-type guanylate cyclase is required for carbon dioxide sensation by *Caenorhabditis elegans*. Proc. Natl. Acad. Sci. USA *108*, 254–259.

Hukema, R.K., Rademakers, S., and Jansen, G. (2008). Gustatory plasticity in *C. elegans* involves integration of negative cues and NaCl taste mediated by serotonin, dopamine, and glutamate. Learn. Mem. *15*, 829–836.

Izquierdo, A., Newman, T.K., Higley, J.D., and Murray, E.A. (2007). Genetic modulation of cognitive flexibility and socioemotional behavior in rhesus monkeys. Proc. Natl. Acad. Sci. USA *104*, 14128–14133.

Jékely, G., Melzer, S., Beets, I., Kadow, I.C.G., Koene, J., Haddad, S., and Holden-Dye, L. (2018). The long and the short of it - a perspective on peptidergic regulation of circuits and behaviour. J. Exp. Biol. *221*, jeb166710–jeb166714.

Jones, S.L., and Svitkina, T.M. (2016). Axon initial segment cytoskeleton: architecture, development, and role in neuron polarity. Neural Plast. *2016*, 6808293.

Kaletsky, R., Lakhina, V., Arey, R., Williams, A., Landis, J., Ashraf, J., and Murphy, C.T. (2016). The *C. elegans* adult neuronal IIS/FOXO transcriptome reveals adult phenotype regulators. Nature *529*, 92–96.

Kim, K., and Li, C. (2004). Expression and regulation of an FMRFamide-related neuropeptide gene family in *Caenorhabditis elegans*. J. Comp. Neurol. *475*, 540–550.

Kiontke, K.C., Félix, M.A., Ailion, M., Rockman, M.V., Braendle, C., Pénigault, J.B., and Fitch, D.H.A. (2011). A phylogeny and molecular barcodes for *Caenorhabditis*, with numerous new species from rotting fruits. BMC Evol. Biol. *11*, 339.

Kizhatil, K., Baker, S.A., Arshavsky, V.Y., and Bennett, V. (2009). Ankyrin-G promotes cyclic nucleotide-gated channel transport to rod photoreceptor sensory cilia. Science *323*, 1614–1617.

Kodama-Namba, E., Fenk, L.A., Bretscher, A.J., Gross, E., Busch, K.E., and de Bono, M. (2013). Cross-modulation of homeostatic responses to temperature, oxygen and carbon dioxide in *C. elegans*. PLoS Genet. *9*, e1004011.

Langmead, B., and Salzberg, S.L. (2012). Fast gapped-read alignment with Bowtie 2. Nat. Methods *9*, 357–359.

Laurent, P., Soltesz, Z., Nelson, G.M., Chen, C., Arellano-Carbajal, F., Levy, E., and de Bono, M. (2015). Decoding a neural circuit controlling global animal state in *C. elegans*. eLife *4*, https://doi.org/10.7554/eLife.04241.

Leterrier, C., Clerc, N., Rueda-Boroni, F., Montersino, A., Dargent, B., and Castets, F. (2017). Ankyrin G membrane partners drive the establishment and maintenance of the axon initial segment. Front. Cell. Neurosci. *11*, 6.

Li, H., and Durbin, R. (2009). Fast and accurate short read alignment with Burrows-Wheeler transform. Bioinformatics *25*, 1754–1760.

Li, H., Handsaker, B., Wysoker, A., Fennell, T., Ruan, J., Homer, N., Marth, G., Abecasis, G., and Durbin, R.; 1000 Genome Project Data Processing Subgroup (2009). The sequence alignment/map format and SAMtools. Bioinformatics *25*, 2078–2079.

Maniar, T.A., Kaplan, M., Wang, G.J., Shen, K., Wei, L., Shaw, J.E., Koushika, S.P., and Bargmann, C.I. (2011). UNC-33 (CRMP) and ankyrin organize microtubules and localize kinesin to polarize axon-dendrite sorting. Nat. Neurosci. *15*, 48–56.

Martínez-Velázquez, L.A., and Ringstad, N. (2018). Antagonistic regulation of trafficking to *Caenorhabditis elegans* sensory cilia by a *Retinal Degeneration 3* homolog and retromer. Proc. Natl. Acad. Sci. USA *115*, E438–E447.

McDonald, J.H. (2009). Handbook of biological statistics (Sparky House Publishing).

McGrath, P.T. (2013). Varieties of behavioral natural variation. Curr. Opin. Neurobiol. *23*, 24–28.

McGrath, P.T., Rockman, M.V., Zimmer, M., Jang, H., Macosko, E.Z., Kruglyak, L., and Bargmann, C.I. (2009). Quantitative mapping of a digenic behavioral trait implicates globin variation in *C. elegans* sensory behaviors. Neuron *61*, 692–699.

McLaren, W., Gil, L., Hunt, S.E., Riat, H.S., Ritchie, G.R.S., Thormann, A., Flicek, P., and Cunningham, F. (2016). The Ensembl variant effect predictor. Genome Biol. *17*, 122.

Mery, F. (2013). Natural variation in learning and memory. Curr. Opin. Neurobiol. *23*, 52–56.

Mery, F., Belay, A.T., So, A.K.C., Sokolowski, M.B., and Kawecki, T.J. (2007). Natural polymorphism affecting learning and memory in *Drosophila*. Proc. Natl. Acad. Sci. USA *104*, 13051–13055.

Milne, I., Stephen, G., Bayer, M., Cock, P.J.A., Pritchard, L., Cardle, L., Shaw, P.D., and Marshall, D. (2013). Using Tablet for visual exploration of second-generation sequencing data. Brief. Bioinform. *14*, 193–202.

Minevich, G., Park, D.S., Blankenberg, D., Poole, R.J., and Hobert, O. (2012). CloudMap: a cloud-based pipeline for analysis of mutant genome sequences. Genetics *192*, 1249–1269.

Monteiro, P., and Feng, G. (2017). SHANK proteins: roles at the synapse and in autism spectrum disorder. Nat. Rev. Neurosci. *18*, 147–157.

Niemelä, P.T., Vainikka, A., Forsman, J.T., Loukola, O.J., and Kortet, R. (2013). How does variation in the environment and individual cognition explain the existence of consistent behavioral differences? Ecol. Evol. *3*, 457–464.

O'Halloran, D.M., Hamilton, O.S., Lee, J.I., Gallegos, M., and L'Etoile, N.D. (2012). Changes in cGMP levels affect the localization of EGL-4 in AWC in *Caenorhabditis elegans*. PLoS ONE *7*, e31614.

Otsuka, A.J., Franco, R., Yang, B., Shim, K.H., Tang, L.Z., Zhang, Y.Y., Boontrakulpoontawee, P., Jeyaprakash, A., Hedgecock, E., Wheaton, V.I., et al. (1995). An ankyrin-related gene (*unc-44*) is necessary for proper axonal guidance in *Caenorhabditis elegans*. J. Cell Biol. *129*, 1081–1092.

Owen, G.R., and Brenner, E.A. (2012). Mapping molecular memory: navigating the cellular pathways of learning. Cell. Mol. Neurobiol. *32*, 919–941.

Persson, A., Gross, E., Laurent, P., Busch, K.E., Bretes, H., and de Bono, M. (2009). Natural variation in a neural globin tunes oxygen sensing in wild *Caenorhabditis elegans*. Nature *458*, 1030–1033.

Peymen, K., Watteyne, J., Frooninckx, L., Schoofs, L., and Beets, I. (2014). The FMRFamide-like peptide family in nematodes. Front. Endocrinol. (Lausanne) *5*, 90.

Picelli, S., Faridani, O.R., Björklund, A.K., Winberg, G., Sagasser, S., and Sandberg, R. (2014). Full-length RNA-seq from single cells using Smart-seq2. Nat. Protoc. *9*, 171–181.

Pigliucci, M., Murren, C.J., and Schlichting, C.D. (2006). Phenotypic plasticity and evolution by genetic assimilation. J. Exp. Biol. *209*, 2362–2367.

Pline, M., and Dusenbery, D.B. (1987). Responses of plant-parasitic nematode*Meloidogyne incognita* to carbon dioxide determined by video camera-computer tracking. J. Chem. Ecol. *13*, 873–888.

Prabhakar, N.R., and Semenza, G.L. (2015). Oxygen sensing and homeostasis. Physiology (Bethesda) *30*, 340–348.

Prieto-Godino, L.L., Rytz, R., Cruchet, S., Bargeton, B., Abuin, L., Silbering, A.F., Ruta, V., Dal Peraro, M., and Benton, R. (2017). Evolution of acid-sensing olfactory circuits in Drosophilids. Neuron *93*, 661–676.

R Development Core Team (2015). R: a language and environment for Statistical Computing (R Foundation for Statistical Computing). https://www.r-project.org/.

Renn, S.C.P., and Schumer, M.E. (2013). Genetic accommodation and behavioural evolution: insights from genomic studies. Anim. Behav. *85*, 1012–1022.

Richaud, A., Zhang, G., Lee, D., Lee, J., and Félix, M.A. (2018). The local coexistence pattern of selfing genotypes in *Caenorhabditis elegans* natural metapopulations. Genetics *208*, 807–821.

Romanos, T.R., Petersen, J.G., and Pocock, R. (2017). Control of neuropeptide expression by parallel activity-dependent pathways in Caenorhabditis elegans. Sci. Rep. *7*, 38734.

Sachse, S., Rueckert, E., Keller, A., Okada, R., Tanaka, N.K., Ito, K., and Vosshall, L.B. (2007). Activity-dependent plasticity in an olfactory circuit. Neuron *56*, 838–850.

Schindelin, J., Arganda-Carreras, I., Frise, E., Kaynig, V., Longair, M., Pietzsch, T., Preibisch, S., Rueden, C., Saalfeld, S., Schmid, B., et al. (2012). Fiji: an open-source platform for biological-image analysis. Nat. Methods *9*, 676–682.

Schmalhausen, I.I. (1949). Factors of Evolution (Blakiston).

Sivadas, P., Dienes, J.M., St Maurice, M., Meek, W.D., and Yang, P. (2012). A flagellar A-kinase anchoring protein with two amphipathic helices forms a structural scaffold in the radial spoke complex. J. Cell Biol. *199*, 639–651.

Smith, E.S., Martinez-Velazquez, L., and Ringstad, N. (2013). A chemoreceptor that detects molecular carbon dioxide. J. Biol. Chem. *288*, 37071–37081.

Smith, K.R., Kopeikina, K.J., Fawcett-Patel, J.M., Leaderbrand, K., Gao, R., Schürmann, B., Myczek, K., Radulovic, J., Swanson, G.T., and Penzes, P. (2014). Psychiatric risk factor ANK3/ankyrin-G nanodomains regulate the structure and function of glutamatergic synapses. Neuron *84*, 399–415.

Stiernagle, T. (2006). Maintenance of *C. elegans* (WormBook). http://wormbase.org.

Taghert, P.H., and Nitabach, M.N. (2012). Peptide neuromodulation in invertebrate model systems. Neuron *76*, 82–97.

Thompson, O., Edgley, M., Strasbourger, P., Flibotte, S., Ewing, B., Adair, R., Au, V., Chaudhry, I., Fernando, L., Hutter, H., et al. (2013). The million mutation project: a new approach to genetics in *Caenorhabditis elegans*. Genome Res. *23*, 1749–1762.

Troemel, E.R., Félix, M.A., Whiteman, N.K., Barrière, A., and Ausubel, F.M. (2008). Microsporidia are natural intracellular parasites of the nematode *Caenorhabditis elegans*. PLoS Biol. *6*, 2736–2752.

Tursun, B., Cochella, L., Carrera, I., and Hobert, O. (2009). A toolkit and robust pipeline for the generation of fosmid-based reporter genes in *C. elegans*. PLoS ONE *4*, e4625.

Van der Auwera, G.A., Carneiro, M.O., Hartl, C., Poplin, R., Del Angel, G., Levy-Moonshine, A., Jordan, T., Shakir, K., Roazen, D., Thibault, J., et al. (2013). From FastQ data to high confidence variant calls: the Genome Analysis Toolkit best practices pipeline. Curr. Protoc. Bioinformatics *43*, 11.10.1-33.

Viglierchio, D.R. (1990). Carbon dioxide sensing by *Panagrellus silusiae* and *Ditylenchus dipsaci*. Revue De Nematologie *13*, 425–432.

Vulesevic, B., McNeill, B., and Perry, S.F. (2006). Chemoreceptor plasticity and respiratory acclimation in the zebrafish *Danio rerio*. J. Exp. Biol. *209*, 1261–1273.

Waddington, C.H. (1942). Canalization of development and the inheritance of acquired characters. Nature *150*, 563–565.

Waddington, C.H. (1953). Genetic assimilation of an acquired character. Evolution *7*, 118–126.

Weber, K.P., De, S., Kozarewa, I., Turner, D.J., Babu, M.M., and de Bono, M. (2010). Whole genome sequencing highlights genetic changes associated with laboratory domestication of *C. elegans*. PLoS ONE *5*, e13922.

West-Eberhard, M.J. (2005). Developmental plasticity and the origin of species differences. Proc. Natl. Acad. Sci. USA *102* (*Suppl 1*), 6543–6549.

Ye, K., Schulz, M.H., Long, Q., Apweiler, R., and Ning, Z. (2009). Pindel: a pattern growth approach to detect break points of large deletions and medium sized insertions from paired-end short reads. Bioinformatics *25*, 2865–2871.

Yin, J.A., Gao, G., Liu, X.J., Hao, Z.Q., Li, K., Kang, X.L., Li, H., Shan, Y.H., Hu, W.L., Li, H.P., and Cai, S.Q. (2017). Genetic variation in glia-neuron signalling modulates ageing rate. Nature *551*, 198–203.

Yu, S., Avery, L., Baude, E., and Garbers, D.L. (1997). Guanylyl cyclase expression in specific sensory neurons: a new family of chemosensory receptors. Proc. Natl. Acad. Sci. USA *94*, 3384–3387.

Zhang, Y., Bertolino, A., Fazio, L., Blasi, G., Rampino, A., Romano, R., Lee, M.L.T., Xiao, T., Papp, A., Wang, D., and Sadée, W. (2007). Polymorphisms in human dopamine D2 receptor gene affect gene expression, splicing, and neuronal activity during working memory. Proc. Natl. Acad. Sci. USA *104*, 20552–20557.

Zhang, G., Sachse, M., Prevost, M.C., Luallen, R.J., Troemel, E.R., and Félix, M.A. (2016). A Large Collection of Novel Nematode-Infecting Microsporidia and Their Diverse Interactions with *Caenorhabditis elegans* and Other Related Nematodes. PLoS Pathog. *12*, e1006093.

Zimmer, M., Gray, J.M., Pokala, N., Chang, A.J., Karow, D.S., Marletta, M.A., Hudson, M.L., Morton, D.B., Chronis, N., and Bargmann, C.I. (2009). Neurons detect increases and decreases in oxygen levels using distinct guanylate cyclases. Neuron *61*, 865–879.

STAR★METHODS

KEY RESOURCES TABLE

REAGENT or RESOURCE	SOURCE	IDENTIFIER
Antibodies		
GFP-Trap Agarose	ChromoTek	Cat#gta-20; RRID: AB_2631357
Bacterial and Virus Strains		
E. coli: Strain OP50	*Caenorhabditis* Genetics Center	Wormbase: OP50; RRID: WB-STRAIN:OP50
Chemicals, Peptides, and Recombinant Proteins		
Dermabond tissue adhesive for worm glueing	Ethicon	Cat#AHV12
Critical Commercial Assays		
SuperScript II reverse transcriptase	Invitrogen	Cat#18064-014
KAPA Hifi HotStart kit	KAPA Biosystems	Cat#KK2601
Ampure XP beads	Beckman Coulter	Cat#A63881
Nextera XT DNA sample preparation kit	Illumina	Cat#FC-131-1096
Deposited Data		
Genome sequence data of JU1249 and JU2825	This paper	NCBI: PRJNA514933
Genome sequence data of replicate populations for QTL mapping	This paper	NCBI: PRJNA515248
RNA-Seq data of sorted BAG neurons	This paper	GEO: GSE135687
Experimental Models: Organisms/Strains		
C. elegans: Strain AX1796: *glb-5(Haw) V; npr-1(g320) X*	de Bono lab; Persson et al., 2009	AX1796
C. elegans: Strain LSJ1: Bristol strain	*Caenorhabditis* Genetics Center	RRID: WB-STRAIN:LSJ1
C. angaria: Strain RGD1: *Caenorhabditis angaria* wild isolate	*Caenorhabditis* Genetics Center	RRID: WB-STRAIN:RGD1
C. latens: Strain VX80: *Caenorhabditis latens* wild isolate	*Caenorhabditis* Genetics Center	RRID: WB-STRAIN:VX80
C. japonica: Strain DF5081: *Caenorhabditis japonica* wild isolate	*Caenorhabditis* Genetics Center	RRID: WB-STRAIN:DF5081
C. wallacei: Strain JU1904: *Caenorhabditis wallacei* wild isolate	*Caenorhabditis* Genetics Center	RRID: WB-STRAIN:JU1904
C. tropicalis: Strain JU1373: *Caenorhabditis tropicalis* wild isolate	*Caenorhabditis* Genetics Center	RRID: WB-STRAIN:JU1373
C. briggsae: Strain HK105: *Caenorhabditis briggsae* wild isolate	*Caenorhabditis* Genetics Center	RRID: WB-STRAIN:HK105
C. nigoni: Strain JU1422: *Caenorhabditis nigoni* wild isolate	*Caenorhabditis* Genetics Center	RRID: WB-STRAIN:JU1422
C. sinica: Strain JU800: *Caenorhabditis sinica* wild isolate	*Caenorhabditis* Genetics Center	RRID: WB-STRAIN:JU800
C. elegans: Strain ED3011: *C. elegans* wild isolate	*Caenorhabditis* Genetics Center	RRID: WB-STRAIN:ED3011
C. elegans: Strain ED3073: *C. elegans* wild isolate	*Caenorhabditis* Genetics Center	RRID: WB-STRAIN:ED3073
C. elegans: Strain EG4946: *C. elegans* wild isolate	*Caenorhabditis* Genetics Center	RRID: WB-STRAIN:EG4946
C. elegans: Strain JU258: *C. elegans* wild isolate	*Caenorhabditis* Genetics Center	RRID: WB-STRAIN:JU258
C. elegans: Strain JU561: *C. elegans* wild isolate	*Caenorhabditis* Genetics Center	RRID: WB-STRAIN:JU561
C. elegans: Strain JU1088: *C. elegans* wild isolate	*Caenorhabditis* Genetics Center	RRID: WB-STRAIN:JU1088
C. elegans: Strain JU1248: *C. elegans* wild isolate	M.-A. Félix; Troemel et al., 2008	RRID: WB-STRAIN:JU1248
C. elegans: Strain JU1543: *C. elegans* wild isolate	M.-A. Félix	RRID: WB-STRAIN:JU1543
C. elegans: Strain JU2825: *C. elegans* wild isolate	M.-A. Félix	JU2825
C. elegans: Strain MY16: *C. elegans* wild isolate	*Caenorhabditis* Genetics Center	RRID: WB-STRAIN:MY16

(*Continued on next page*)

Continued

REAGENT or RESOURCE	SOURCE	IDENTIFIER
C. elegans: Strain JU1249: *C. elegans* wild isolate	M.-A. Félix; Zhang et al., 2016	RRID: WB-STRAIN:JU1249
C. elegans: Strain AX613: *npr-1(g320) X*	de Bono lab; Persson et al., 2009	AX613
C. elegans: Strain JU3221: *arcp-1(mfP22) III; npr-1(215F) X; mfEx94 [arcp-1p::arcp-1b::sl2::gfp; myo-2p::dsRed2]* in JU1249 background	This paper	JU3221
C. elegans: Strain AX204: *npr-1(ad609) X*	de Bono lab; Persson et al., 2009	AX204
C. elegans: Strain AX6574: *arcp-1(db1082) III; npr-1(ad609) X* 4x outcrossed	This paper	AX6574
C. elegans: Strain AX7324: *arcp-1(db1082) III; npr-1(ad609) X* 5x outcrossed	This paper	AX7324
C. elegans: Strain AX6723: *arcp-1(db1082) III; npr-1(ad609) X; dbEx975 [arcp-1p::arcp-1b::sl2::gfp; unc-122p::rfp]*	This paper	AX6723
C. elegans: Strain AX7094: *arcp-1(db1082) III; npr-1(ad609) X; dbEx1050 [arcp-1p::arcp-1b::sl2::mKate; lin-44p::gfp]*	This paper	AX7094
C. elegans: Strain AX6720: *arcp-1(db1082) III; npr-1(ad609) X; dbEx974 [arcp-1p::arcp-1a::sl2::gfp; unc-122p::rfp]*	This paper	AX6720
C. elegans: Strain N2: *C. elegans* Bristol strain	*Caenorhabditis* Genetics Center	RRID: WB-STRAIN:N2_(ancestral)
C. elegans: Strain MY10: *C. elegans* wild isolate	*Caenorhabditis* Genetics Center	RRID: WB-STRAIN:MY10
C. elegans: Strain JU1247: *C. elegans* wild isolate	M.-A. Félix, (Troemel et al., 2008)	RRID: WB-STRAIN:JU1247
C. elegans: Strain AX6901: *arcp-1(db1082) III; npr-1(ad609) X; dbEx1002 [fosmid arcp-1p::arcp-1::sl2::gfp; unc-122p::rfp]*	This paper	AX6901
C. elegans: Strain AX6766: *arcp-1(db1082) III; npr-1(ad609) X; dbEx984 [gcy-32p::arcp-1b::sl2::gfp; unc-122p::rfp]*	This paper	AX6766
C. elegans: Strain AX6805: *arcp-1(db1082) III; npr-1(ad609) X; dbEx990 [flp-17p::arcp-1b::sl2::gfp; unc-122p::rfp]*	This paper	AX6805
C. elegans: Strain AX6931: *arcp-1(gk856856) III; npr-1(ad609) X*	This paper	AX6931
C. elegans: Strain AX6929: *arcp-1(gk863317) III; npr-1(ad609) X*	This paper	AX6929
C. elegans: Strain AX6927: *arcp-1(gk852871) III; npr-1(ad609) X*	This paper	AX6927
C. elegans: Strain AX7023: *arcp-1(db1082) III; npr-1(ad609) X; dbEx1035 [gcy-32p::arcp-1b::sl2::mKate; lin-44p::gfp]*	This paper	AX7023
C. elegans: Strain AX7095: *arcp-1(db1082) III; npr-1(ad609) X; dbEx990 [flp-17p::arcp-1b::sl2::gfp; unc-122p::rfp]; dbEx1035 [gcy-32p::arcp-1b::sl2::mKate; lin-44p::gfp]*	This paper	AX7095
C. elegans: Strain AX7179: *gcy-9(n4470) npr-1(ad609) X*	This paper	AX7179
C. elegans: Strain AX7238: *arcp-1(db1082) III; gcy-9(n4470) npr-1(ad609) X*	This paper	AX7238
C. elegans: Strain AX7116: *arcp-1(db1082) III; npr-1(ad609) X; dbIs20 [arcp-1p::gfp::arcp-1b; unc-122p::rfp]*	This paper	AX7116
C. elegans: Strain AX6969: *malt-1(db1194) II; npr-1(ad609) X; dbIs16 [rab-3p::malt-1::gfp; unc-122p::rfp]*	This paper	AX6969

(*Continued on next page*)

Continued

REAGENT or RESOURCE	SOURCE	IDENTIFIER
C. elegans: Strain AX7082: *eif-3.L(db1015) II; npr-1(ad609) X; dbIs19 [rab-3p::eif-3.L::gfp; unc-122p::rfp]*	This paper	AX7082
C. elegans: Strain AX7419: *npr-1(ad609) X dbEx1075 [flp-17p::pde-1b::gfp; unc-122p::rfp]*	This paper	AX7419
C. elegans: Strain AX7422: *arcp-1(db1082) III; npr-1(ad609) X; dbEx1075 [flp-17p::pde-1b::gfp; unc-122p::rfp]*	This paper	AX7422
C. elegans: Strain AX2272: *pde-1(ok2924) I; npr-1(ad609) X*	de Bono lab; Couto et al., 2013	AX2272
C. elegans: Strain AX7453: *pde-1(ok2924) I; arcp-1(db1082) III; npr-1(ad609) X*	This paper	AX7453
C. elegans: Strain AX6881: *npr-1(ad609) X dbEx [flp-17p::YC3.60]*	This paper	AX6881
C. elegans: Strain AX6893: *arcp-1(db1082) III; npr-1(ad609) X; dbEx [flp-17p::YC3.60]*	This paper	AX6893
C. elegans: Strain AX7842: *arcp-1(db1082) III; npr-1(ad609) X; dbEx [flp-17p::YC3.60]; dbEx1035 [gcy-32p::arcp-1b::sl2::mKate; lin-44::gfp]*	This paper	AX7842
C. elegans: Strain AX7845: *arcp-1(db1082) III; npr-1(ad609) X; dbEx [flp-17p::YC3.60] dbEx1172 [gcy-33p::arcp-1b::sl2::mKate; unc-122p::gfp]*	This paper	AX7845
C. elegans: Strain AX3516: *npr-1(ad609) X; dbEx614 [gcy-37p::YC2.60; unc-122p::rfp]*	de Bono lab; Fenk and de Bono, 2017	AX3516
C. elegans: Strain AX6877: *arcp-1(db1082) III; npr-1(ad609) X; dbEx614 [gcy-37p::YC2.60; unc-122p::rfp]*	This paper	AX6877
C. elegans: Strain AX3432: *npr-1(ad609) X; dbEx623 [flp-17p::YC2.60; F15E11.1::mCherry]*	de Bono lab; Gross et al., 2014	AX3432
C. elegans: Strain AX7182: *arcp-1(db1082) III; npr-1(ad609) X; dbEx623 [flp-17p::YC2.60; F15E11.1::mCherry]*	This paper	AX7182
C. elegans: Strain AX7656: *gcy-33(ok232) V; gcy-31(ok296) npr-1(ad609) X*	This paper	AX7656
C. elegans: Strain AX7657: *arcp-1(db1082) III; gcy-33(ok232) V; gcy-31(ok296) npr-1(ad609) X*	This paper	AX7657
C. elegans: Strain AX7362: *npr-1(ad609) X; wzIs132 [gcy-9p::gcy-9::dsRed]*	This paper	AX7362
C. elegans: Strain AX7361: *arcp-1(db1082) III; npr-1(ad609) X; wzIs132 [gcy-9p::gcy-9::dsRed]*	This paper	AX7361
C. elegans: Strain AX7366: *npr-1(ad609) X; wzEx156 [gcy-9p::tax-4::gfp]*	This paper	AX7366
C. elegans: Strain AX7365: *arcp-1(db1082) III; npr-1(ad609) X; wzEx156 [gcy-9p::tax-4::gfp]*	This paper	AX7365
C. elegans: Strain AX2997: *gcy-33(ok232) V; npr-1(ad609) X; dbEx [flp-17p::gcy-33::gfp; unc-122p::rfp]*	de Bono lab; Gross et al., 2014	AX2997
C. elegans: Strain AX7315: *arcp-1(db1082) III; gcy-33(ok232) V; npr-1(ad609) X; dbEx [flp-17p::gcy-33::gfp; unc-122p::rfp]*	This paper	AX7315
C. elegans: Strain AX6516: *npr-1(ad609) X; dbEx1053 [gcy-37p::gcy-35::HA::gfp::sl2::mCherry]*	This paper	AX6516
C. elegans: Strain AX7278: *arcp-1(db1082) III; npr-1(ad609) X; dbEx1053 [gcy-37p::gcy-35::HA::gfp::sl2::mCherry]*	This paper	AX7278
C. elegans: Strain AX7019: *arcp-1(db1082) III; npr-1(ad609) X; dbEx1033 [flp-17p::gfp; unc-122p::rfp]*	This paper	AX7019

(*Continued on next page*)

Continued

REAGENT or RESOURCE	SOURCE	IDENTIFIER
C. elegans: Strain AX7021: *npr-1(ad609) X; dbEx1033 [flp-17p::gfp; unc-122p::rfp]*	This paper	AX7021
C. elegans: Strain AX7268: *npr-1(ad609) X; ynIs34 [flp-19p::gfp]*	This paper	AX7268
C. elegans: Strain AX7271: *arcp-1(db1082) III; npr-1(ad609) X; ynIs34 [flp-19p::gfp]*	This paper	AX7271
C. elegans: Strain AX7279: *pde-1(ok2924) I; npr-1(ad609) X; ynIs34 [flp-19p::gfp]*	This paper	AX7279
C. elegans: Strain AX7272: *arcp-1(db1082) III; npr-1(ad609) X; ynIs34 [flp-19p::gfp]; dbEx1063 [flp-17p::arcp-1b::sl2::mKate; unc-122p::gfp]*	This paper	AX7272
C. elegans: Strain AX7273: *arcp-1(db1082) III; npr-1(ad609) X; ynIs34 [flp-19p::gfp] dbEx1035 [gcy-32p::arcp-1b::sl2::mKate; lin-44::gfp]*	This paper	AX7273
C. elegans: Strain AX7550: *pde-1(ok2924) I; arcp-1(db1082) III; npr-1(ad609) X; ynIs34 [flp-19p::gfp]*	This paper	AX7550
C. elegans: Strain AX7722: *ynIs34 [flp-19p::gfp]* backcrossed 10x in JU2825 background	This paper	AX7722
C. elegans: Strain AX7724: *ynIs34 [flp-19p::gfp]* backcrossed 10x in JU1249 background	This paper	AX7724
C. elegans: Strain AX7726: *dbEx1063 [flp-17p::arcp-1b::sl2::mKate; unc-122p::gfp]; ynIs34 [flp-19p::gfp]* backcrossed 10x in JU1249 background	This paper	AX7726
C. elegans: Strain AX7210: *npr-1(ad609) X; ynIs64 [flp-17p::gfp]*	This paper	AX7210
C. elegans: Strain AX7208: *arcp-1(db1082) III; npr-1(ad609) X; ynIs64 [flp-17p::gfp]*	This paper	AX7208
C. elegans: Strain AX7321: *flp-19(ok2460) npr-1(ad609) X*	This paper	AX7321
C. elegans: Strain AX7322: *arcp-1(db1082) III; flp-19(ok2460) npr-1(ad609) X*	This paper	AX7322
C. elegans: Strain AX7754: *arcp-1(db1082) III; npr-1(ad609) X; dbEx1171 [gcy-33p::gfp (sas); unc-122p::rfp]*	This paper	AX7754
C. elegans: Strain AX7760: *arcp-1(db1082) III; npr-1(ad609) X; dbEx1173 [gcy-33p::flp-19 (sas); unc-122p::rfp]*	This paper	AX7760
C. elegans: Strain AX7788: *arcp-1(db1082) III; npr-1(ad609) X; dbEx1178 [gcy-32p::gfp (sas); unc-122p::rfp]*	This paper	AX7788
C. elegans: Strain AX7678: *arcp-1(db1082) III; npr-1(ad609) X; dbEx1153 [gcy-32p::flp-19 (sas); unc-122p::gfp]*	This paper	AX7678
C. elegans: Strain AX7793: *npr-1(ad609) X; dbEx1173 [gcy-33p::flp-19 (sas); unc-122p::rfp]*	This paper	AX7793
C. elegans: Strain AX7437: *npr-1(ad609) X; dbEx1077 [flp-17p::flp-19::sl2::mKate; unc-122p::gfp]*	This paper	AX7437
Oligonucleotides		
Primers used in this study	This paper	Table S2
Software and Algorithms		
FlyCapture	Point Grey Research	https://www.flir.com/products/flycapture-sdk
Zentracker	Laurent et al., 2015	https://github.com/wormtracker/zentracker

(*Continued on next page*)

Continued

REAGENT or RESOURCE	SOURCE	IDENTIFIER
Neuron Analyzer	Laurent et al., 2015	https://github.com/neuronanalyser/neuronanalyser
RStudio 0.99.903	R Development Core Team, 2015	https://www.R-project.org
Pindel	Ye et al., 2009	http://gmt.genome.wustl.edu/packages/pindel/
Variant Effect Predictor (VEP)	McLaren et al., 2016	www.ensembl.org/info/docs/tools/vep/index.html
Tablet 1.16.09.06	Milne et al., 2013	https://ics.hutton.ac.uk/tablet/
BWA 0.7.8-R455	Li and Durbin, 2009	http://bio-bwa.sourceforge.net/bwa.shtml
Samtools 1.2	Li et al., 2009	http://samtools.sourceforge.net/
Picard 1.114	Broad Institute	http://broadinstitute.github.io/picard/
GATK 3.2-2	Van der Auwera et al., 2013	https://software.broadinstitute.org/gatk/
Bowtie2 0.11.0	Langmead and Salzberg, 2012	http://bowtie-bio.sourceforge.net/bowtie2/index.shtml
rRNA remover code	This paper	https://github.com/lmb-seq/RNA-Seq_utilities
Code for concatenating FASTQ files	This paper	https://github.com/lmb-seq/RNA-Seq_utilities
PRAGUI RNA-Seq analysis pipeline	This paper	https://github.com/lmb-seq/PRAGUI
Mascot	Matrix Science	http://www.matrixscience.com/
Scaffold	Proteome Software Inc	http://www.proteomesoftware.com/products/scaffold/
Prism 7.0	GraphPad Software	https://www.graphpad.com
MATLAB R2014b 8.4	Mathworks	https://www.mathworks.com/products/matlab.html
Metamorph	Molecular Devices	https://www.moleculardevices.com/products/cellular-imaging-systems/acquisition-and-analysis-software/metamorph-microscopy#gref
Fiji (ImageJ)	Schindelin et al., 2012	https://imagej.net/Fiji
Imaris	Bitplane	https://imaris.oxinst.com/
Other		
Certified gas mixes	BOC	N/A

LEAD CONTACT AND MATERIALS AVAILABILITY

Further information and requests for resources and reagents should be directed to and will be fulfilled by the Lead Contact, Mario de Bono (debono@mrc-lmb.cam.ac.uk, mdebono@ist.ac.at).

EXPERIMENTAL MODEL AND SUBJECT DETAILS

Animals

C. elegans and other *Caenorhabditis* species were maintained under standard conditions (Stiernagle, 2006) on nematode growth medium (NGM) plates seeded with *E. coli* OP50. Young adult hermaphrodites were used in all experiments. For gonochoristic *Caenorhabditis* species, young adult females were used. For a list of strains and transgene details, see Table S1 and the Key Resources Table.

The mutations in *arcp-1* alleles obtained by forward genetics, and in the JU1249 wild isolate, are shown in Figure S5A. The *C. elegans* strain JU1249 was isolated from a rotten apple collected in 2007 in Santeuil, France (Zhang et al., 2016). A detailed description of the forward genetic screen that isolated the *db1082* allele will be described elsewhere. Causal variants in aggregation-defective mutants from this screen were identified by SNP-based mapping in combination with WGS (Minevich et al., 2012).

Microbe strains

The *Escherichia coli* OP50 strain was used as a food source for *C. elegans* and other *Caenorhabditis* species.

METHOD DETAILS

Molecular biology

Transgenes were cloned using the Multisite Gateway Three-Fragment cloning system (12537-023, Invitrogen) into pDESTR4R3 II. For transgenic lines, the promoter lengths were: *arcp-1p* (1.2 kb for *arcp-1a* and 2 kb for *arcp-1b*), *flp-17p* (3.3 kb), *gcy-32p* (0.6 kb), and *gcy-33p* (1.0 kb). For rescue experiments, cDNA of *arcp-1* isoforms was amplified and cloned into pDONR221, using primers listed in Table S2.

For immunoprecipitation and subcellular localization of ARCP-1, a functional *arcp-1p::gfp::arcp-1b* transgene was made by fusing GFP coding sequences upstream of the *arcp-1b* cDNA sequence. To investigate the subcellular localization of PDE-1 in BAG neurons, the *pde-1b* cDNA sequence was cloned into pDONR221 using primers listed in Table S2. This plasmid was used to generate a *flp-17p::pde-1b::gfp* transgene, by cloning the GFP reporter sequence in frame and downstream of the *pde-1b* cDNA sequence. Details of strains and transgenes used to study the subcellular localization of *gcy-9*, *tax-4*, *gcy-33* and *gcy-35* are provided in Table S1. The *gcy-9p::gcy-9::mCherry* and *gcy-9p::tax-4::gfp* strains were a kind gift from Dr. Niels Ringstad (New York University School of Medicine, USA).

For *flp-19* RNAi, 469 bp of *flp-19* cDNA starting from the sequence GCTTTTCCTGTTAA was cloned in both the sense and antisense orientations. For cell-specific RNAi experiments, we expressed these fragments in BAG using the *gcy-33p* (1.0 kb) and in URX neurons using *gcy-32p* (0.6 kb). To overexpress *flp-19* in BAG, we amplified *flp-19* cDNA using primers listed in Table S2, and fused this sequence to the *flp-17* (3.3 kb) promoter.

To characterize the expression pattern of *arcp-1*, we made a fluorescent reporter transgene by fosmid recombineering. pBALU9 was used to amplify a reporter cassette, containing the *gpd-2* intergenic SL2 sequence and a GFP coding sequence, which was inserted downstream of the *arcp-1* stop codon in the WRM0633bA06 fosmid as described (Tursun et al., 2009). The reporter strain for *flp-19* neuropeptide expression (*flp-19p::gfp*) was a kind gift from Dr. Roger Pocock (Monash University, Australia).

Genotyping of natural polymorphisms

Polymorphisms of *npr-1*, *glb-5*, *nath-10* and *arcp-1* genes in *C. elegans* wild isolates were genotyped by PCR. Primers used are listed in Table S2.

Behavioral assays

All experiments used young adult hermaphrodite animals, therefore sample stratification was not required within each genotype/condition. For most experiments, measurements were scored using an automated algorithm so blind scoring was not undertaken: see each subsection for details. For details of statistical tests, see the relevant Figure legend for each experiment and also the subsection ''Quantification and Statistical Analysis.'' All recordings that passed the automated analysis pipeline were included in the final dataset. For rescue and RNAi experiments, behavioral responses and phenotypes were confirmed by testing at least two independent transgenic strains.

Locomotory responses to CO_2 and O_2

Behavioral responses to gas stimuli were assayed as described (Fenk and de Bono, 2017; Laurent et al., 2015). Animals were acclimated to different O_2 levels by growing them for one generation at 21% O_2 (room air) or in a gas-controlled incubator kept at 7% O_2. For each assay, 20-30 young adult hermaphrodites were transferred onto NGM plates seeded 16–20 h earlier with 20 μL of *E. coli* OP50. To control gas levels experienced by *C. elegans*, animals were placed under a 200 μm deep square polydimethylsiloxane (PDMS) chamber with inlets connected to a PHD 2000 Infusion syringe pump (Harvard apparatus). Humidified gas mixtures were delivered at a flow rate of 3.0 ml/min. Behavioral responses to changes in O_2 levels were measured by exposing animals to a stimulus train of 7% O_2 - 21% O_2 - 7% O_2 (upshift) or 21% O_2 - 7% O_2 - 21% O_2 (downshift), in which each stimulus comprised a 3 min time interval. Locomotory responses to CO_2 were measured by exposing animals to a series of 0% CO_2 (3 min) - X% CO_2 (3 min) - 0% CO_2 (3 min), with X corresponding to 1%, 3%, 5% or 10% CO_2 depending on the experiment. In all CO_2 assays, a background level of 7% O_2 was used. Movies were recorded during the stimulus train using FlyCapture (Point Grey Research) on a Leica MZ6 dissecting microscope with a Point Grey Grasshopper camera running at 2 frames/s. Video recording was started 2 min after animals were placed under the PDMS chamber to ensure that the initial environment was in a steady state. In assays where we prolonged the exposure to 7% O_2 before CO_2 stimulation, video recording was started 21 min after animals were placed under the PDMS chamber kept at 7% O_2, and animals were stimulated with 3% CO_2 at t = 24 min. Videos were analyzed in Zentracker, a custom-written MATLAB software (https://github.com/wormtracker/zentracker). All worms in the field of view were analyzed except those in contact with other animals. Speed was calculated as instantaneous centroid displacement between successive frames. Omega turns were identified as described (Laurent et al., 2015). In total 2-4 assay plates with 20-30 animals per plate were tested per day, and each genotype or condition was assayed in at least two independent experiments. As locomotion measurements were conducted using an automated algorithm, genotypes were not blinded prior to analysis.

Aggregation and bordering behavior

L4 animals were picked to a fresh plate 24 h before the assay. Sixty animals were then repicked to the assay plate (an NGM plate seeded 2 days earlier with 100 μL of *E. coli* OP50), and bordering and aggregation was scored 2 and 6 h later. The scorer was blind to genotype. Behavior was always scored on 2-4 assay plates (each containing 60 animals) per day and tested in at least two independent experiments.

Salt-based associative learning

Gustatory plasticity was tested as described (Beets et al., 2012; Hukema et al., 2008), in a climate-controlled room set at 20°C and 40% relative humidity. Synchronized young adult hermaphrodites were grown at 25°C on culture plates seeded with *E. coli* OP50. Animals were collected and washed three times over a period of 15 min with chemotaxis buffer (CTX, 5 mM KH_2PO_4/K_2HPO_4 pH 6.6, 1 mM $MgSO_4$, and 1 mM $CaCl_2$). Mock-conditioned animals were washed in CTX buffer without NaCl, whereas NaCl-conditioned animals were washed in CTX containing 100 mM NaCl for salt conditioning. Salt chemotaxis behavior of mock- and NaCl-conditioned animals was then tested on four-quadrant plates (Falcon X plate, Becton Dickinson Labware) filled with buffered agar (2% agar, 5 mM KH_2PO_4/K_2HPO_4 pH 6.6, 1 mM $MgSO_4$, and 1 mM $CaCl_2$) of which two opposing pairs have been supplemented with 25 mM NaCl. Assay plates were always prepared fresh and left open to solidify and dry for 60 min. Plates were then closed and used on the same day. After the washes, 50 - 150 animals were pipetted on the intersection of the four quadrants and allowed to crawl for 10 min on the quadrant plate. A chemotaxis index was calculated as (n(A) – n(C)) / (n(A) + n(C)) where n(A) is the number of worms within the quadrants containing NaCl and n(C) is the number of worms within the control quadrants without NaCl. The scorer was blind to genotype.

Selection-based QTL mapping

Competition assays

The *C. elegans* strains JU1249 and JU2825 were competed for several generations using different transfer methods. At the start of the assay, ten JU1249 and JU2825 L4 larvae were put together on a 10 cm NGM plate seeded with *E. coli* OP50. Five biological replicates were maintained at 23°C. Before the cultures starved, a small fraction of the population (200 to 400 animals) was used to seed a fresh culture plate. In Treatment A, the worms were harvested with M9 buffer and 2 μL of worm pellet was transferred to the next plate. In Treatment B, an agar cube (chunk) was cut at the edge of the bacterial lawn and deposited onto the next plate. After each transfer, the remaining animals were stored in M9 buffer at −80°C to quantify the relative proportions of JU1249 and JU2825 alleles.

The genomes of JU1249 and JU2825 were sequenced on an Illumina Hiseq4000 at 20x coverage with paired-end 150 bp reads. For each genome, the raw data were aligned to the reference genome (*C. elegans* WS243 masked from http://wormbase.org) and analyzed using BWA, SAMtools, Picard and Genome Analysis Toolkit (GATK) (Li and Durbin, 2009; Li et al., 2009; Van der Auwera et al., 2013). The accession number for the genomic sequence data of JU1249 and JU2825 is NCBI: PRJNA514933 (https://www.ncbi.nlm.nih.gov/genome/?term=PRJNA514933).

From the output BAM files, homozygous SNPs between the two strains were called and filtered with a raw read depth threshold of 10-300. Allele quantification for the III_663310 polymorphism was performed using pyrosequencing as previously described (Duveau and Félix, 2012). Primers for pyrosequencing are listed in Table S2. In brief, *C. elegans* samples harvested after each transfer were centrifuged at 3,000 rpm for 2 min. Lysates of 2 μL of the worm pellets were used as PCR templates and allele frequencies were quantified with a pyrosequencer (PyroMark Q96 ID; Biotage). The accuracy of this quantification method was estimated by measuring the allele frequencies of PCR products that were amplified using *C. elegans* lysates of known proportions of JU1249 and JU2825 individual L4 larvae. On average, a 2% difference was measured between expected and observed allele frequencies.

Selection-based QTL mapping experiment

Segregating populations were generated by crossing the parental JU1249 and JU2825 *C. elegans* wild isolates in both directions, using ten males and two self-sperm exhausted hermaphrodites in each cross. From the F1 progeny, eight biological replicates were set up to generate F2 by crossing again ten males and two self-sperm exhausted hermaphrodites. From each F2 replicate, six males and two L4 stage hermaphrodites were crossed to have plenty of F3 progeny. In the F3 generation, two paired founding populations of 200 L4 larvae (100 from each initial cross direction) were set up per replicate and submitted to contrasted selection regimes. Treatment A transferred worms through liquid harvest and Treatment B by chunking, as described for the competition assay above. In both Treatments, 200-400 animals were transferred before starvation. Males were maintained in the population during each of the first five transfers by picking 50 males. In total 19-20 transfers were done for populations under Treatment A and 17-19 transfers for populations with Treatment B. Genomic DNA of each population (about 10^5 individuals) was extracted as a pool and sequenced as described above.

The reads of each pool were aligned to the N2 reference genome as described above. The BAM files were filtered for allele information on the positions of homozygous SNPs between the two parents. Allele frequencies were analyzed in each pool. A Cochran-Mantel-Haenszel (CMH) test was used to analyze the consistency of the allele frequency difference between populations with different treatments among the eight replicates, except in the genomic positions 4396879-16406352 on Chromosome IV, where replicate 3 was excluded because one parental genome was fixed in this region in both treatments (McDonald, 2009). The null hypothesis for this CMH test is an equal distribution of sequence reads between the two treatments, and does not consider noise due to allelic drift in the populations, thus inflating the *-log(p value)*. Drift could not be simulated because, for experimental simplicity, population

sizes and generations were not controlled during the transfers. We note that, although population size in the experiment was low, the mapping had a relatively good resolution due to the number of populations (16 in total), which yielded independent recombination events.

RStudio (v 0.99.903) and packages (ggplot2, plyr, evobiR) were used for statistical analysis, plots of allele frequencies and CMH tests. Pindel (Ye et al., 2009) was used to detect homozygous indels in the candidate region between JU1249 and JU2825, but no additional polymorphism was found. High quality homozygous variants of the parental strains in the candidate region were annotated using VEP (http://www.ensembl.org//useast.ensembl.org/info/docs/tools/vep/index.html?redirectsrc=//www.ensembl.org%2Finfo%2Fdocs%2Ftools%2Fvep%2Findex.html) (McLaren et al., 2016). The *mfP22* deletion was verified by PCR and Sanger sequencing, using primers listed in Table S2. The accession number for the genomic sequence data of the replicate populations is NCBI: PRJNA515248 (https://www.ncbi.nlm.nih.gov/genome/?term=PRJNA515248).

Distribution of *mfP22* allele in wild isolates

To examine the distribution of the *mfP22* deletion in *C. elegans* wild isolates, we monitored the presence of the deletion visually, using Tablet 1.16.09.06 (Milne et al., 2013), for 151 isotypes with whole genome sequences in the CeNDR database (Cook et al., 2016). The *mfP22* allele was only found in JU1249 (Data S2).

Confocal microscopy and image analysis

Confocal images were acquired using a Zeiss LSM 710 microscope or a Nikon Eclipse Ti inverted setup coupled to an Andor Ixon EMCCD camera and a spinning disk confocal unit. Projections of z stacks were generated using Fiji (ImageJ).

Expression of *arcp-1* in URX, AQR and PQR was confirmed by co-expression with a *gcy-32p::mCherry* transgene. Expression in BAG and AWB neurons was verified by crossing *arcp-1* reporter strains with *flp-17p::mCherry* and *str-1p::mCherry* marker strains, respectively. Expression in AWC and ASE was confirmed by co-expression with *ceh-36p::RFP* and *odr-1p::RFP* transgenes. For DiI staining, animals were incubated in DiI solution (0.01 mg/ml) for 3 h and washed with M9 buffer before mounting for confocal microscopy.

To quantify the fluorescence of reporter-tagged proteins in cilia and neuron cell bodies, z stack images were taken on a spinning disk confocal microscope using a 60x lens and 100 ms exposure time. Z-projections of image stacks were generated with Fiji (ImageJ). Regions of interest (ROIs) were selected by centering a 50-pixel by 50-pixel square region over the distal dendrite or soma of the BAG neurons, respectively. All measurements were background-corrected by subtracting the mean values of a 50-pixel by 50-pixel square region drawn outside of the neuron.

To quantify the expression of neuropeptide reporters in BAG soma, z stack images were taken on a spinning disk confocal microscope using a 60x lens and 100 ms exposure time. 3D images were reconstituted using the IMARIS software package (Bitplane). GFP pixel intensities brighter than a threshold value (1000 for *flp-19* and 3000 for *flp-17* reporters) were cropped by creating a surface with 0.25 mm details. The mean pixel intensities inside the surface were calculated after background subtraction.

Calcium imaging

L4 animals expressing a ratiometric yellow cameleon sensor were picked 24 h before imaging. Animals were glued to agarose pads (2% agarose in M9 buffer, 1 mM $CaCl_2$) using Dermabond tissue adhesive (Ethicon) with the nose immersed in a mix of bacterial food (*E. coli* OP50) and M9 buffer. To deliver gas stimuli, glued animals were placed under a microfluidic chamber with inlets connected to a PHD 2000 Infusion syringe pump (Harvard Apparatus) running at a flow rate of 2.5 ml/min. An electronic valve system placed between the syringes and the microfluidic chamber allowed switching between two different gas mixtures in a controlled manner at prespecified time intervals. Imaging data were analyzed using Neuron Analyzer, a customwritten MATLAB program (code available at https://github.com/neuronanalyser/neuronanalyser). As measurements were conducted using an automated algorithm, genotypes were not blinded prior to analysis.

CO_2-evoked Ca^{2+} activity

Animals expressing a *flp-17p::YC3.60* (yellow cameleon 3.60) transgene were used for ratiometric imaging of relative calcium concentration in BAG cell bodies (Bretscher et al., 2011). After immobilization, animals were placed under a microfluidic PDMS chamber and exposed to a 0% CO_2 (3 min) - X% CO_2 (3 min) - 0% CO_2 (3 min) stimulus train, with X corresponding to 1%, 3% or 5% CO_2 depending on the experiment. To measure CO_2-evoked tonic Ca^{2+} activity in BAG, the time interval for CO_2 stimulation was prolonged from 3 min to 18 min. In all experiments, the background O_2 level was 7% O_2. Calcium imaging was done at 2 frames/s on an AZ100 microscope (Nikon) bearing a TwinCam adaptor (Cairn Research) mounted with two ORCAFlash4.0 V2 digital cameras (Hamamatsu) using an AZ Plan Fluor 2x lens with 2x zoom and an exposure time of 500 ms.

O_2-evoked Ca^{2+} activity

We used animals expressing a *gcy-37p::YC2.60* transgene to measure Ca^{2+} activity of URX neurons in response to O_2 stimuli (Fenk and de Bono, 2017). To measure O_2 responses in BAG, we used animals expressing a *flp-17p::YC2.60* transgene (Gross et al., 2014). After immobilization, animals were placed under a Y-shaped microfluidic chamber and exposed to an O_2 upshift (7% - 21% - 7% O_2) in case of URX imaging, or an O_2 downshift (21% - 7% - 21% O_2) for BAG. Each stimulus comprised a 2 min time window. Images were recorded at 2 frames/s with an exposure time of 100 ms for a total of 6 min, on a Zeiss Axiovert inverted microscope with an EMCCD Evolve 512 Deltacamera (Photometrics) and a 40x C-Apochromat lens, using MetaMorph acquisition software (Molecular

Devices). To reduce photobleaching an optical density filter 2.0 or 1.5 was used. Excitation light was passed through an excitation filter for CFP (438/24-25, Semrock) and a dichroic filter for YFP (DiO2-25x36, Semrock). A beam splitter (Optical Insights) was used to separate the cyan and yellow emission light using a dichroic filter for 483/32-25 nm (CFP) and 542/27-25 nm (YFP) (Semrock).

Immunoprecipitation from *C. elegans*

Two independent coIP experiments were performed to identify putative interactors of ARCP-1B. Samples for GFP-ARCP-1B were always processed in parallel with control samples of other cytoplasmic GFP-tagged proteins (MALT-1-GFP and EIF-3.L-GFP), providing negative controls. For coIP experiments, lysis buffer was prepared with 50 mM HEPES (pH 7.4), 1 mM EGTA, 1 mM $MgCl_2$, 100 mM KCl, 10% glycerol, 0.05% Tergitol type-NP40 (Sigma-Aldrich), 1mM DTT, 0.1M PMSF with 1 complete EDTA-free proteinase inhibitor cocktail tablet (Roche Applied Science) per 12 ml. Worms were washed twice in ice-cold M9 and once in ice-cold lysis buffer, and then snap-frozen in liquid nitrogen. Frozen worm pellets (~10 g) were pulverized using a Freezer/Mill (SPEX SamplePrep). Crude extract was clarified at 4°C for 10 min at 20,000 g, and again for 20 min at 100,000 g with a TLA-100 rotor (Beckman Coulter). For immunoprecipitation, samples were incubated with GFP-Trap (ChromoTek) for 4 h at 4°C, then washed 3 times with 50 mM HEPES, 100 mM KCl. Purified complexes were eluted in SDS-sample buffer at 95°C and further fractionated by SDS-PAGE prior to mass spectrometry analysis.

Proteins were identified by Orbitrap-mass spectrometry and MASCOT database searching. Gel samples were destained with 50% v/v acetonitrile and 50 mM ammonium bicarbonate, reduced with 10 mM DTT, and alkylated with 55 mM iodoacetamide. Digestion was with 6 ng/μl trypsin (Promega) overnight at 37°C, and peptides extracted in 2% v/v formic acid 2% v/v acetonitrile, and analyzed by nano-scale capillary LC-MS/MS (Ultimate U3000 HPLC, Thermo Scientific Dionex) at a flow of ~300 nL/min. A C18 Acclaim PepMap100 5 μm, 100 μm x 20 mm nanoViper (Thermo Scientific Dionex), trapped the peptides prior to separation on a C18 Acclaim PepMap100 3 μm, 75 μm x 250 mm nanoViper. Peptides were eluted with an acetonitrile gradient. The analytical column outlet was interfaced via a nano-flow electrospray ionisation source with a linear ion trap mass spectrometer (Orbitrap Velos, Thermo Scientific). Data dependent analysis was performed using a resolution of 30,000 for the full MS spectrum, followed by ten MS/MS spectra in the linear ion trap. MS spectra were collected over a m/z range of 300–2000. MS/MS scans were collected using a threshold energy of 35 for collision-induced dissociation. LC-MS/MS data were searched against the UniProt KB database using Mascot (Matrix Science), with a precursor tolerance of 10 ppm and a fragment ion mass tolerance of 0.8 Da. Two missed enzyme cleavages and variable modifications for oxidised methionine, carbamidomethyl cysteine, pyroglutamic acid, phosphorylated serine, threonine and tyrosine were included. MS/MS data were validated using the Scaffold program (Proteome Software Inc).

RNA-seq of sorted BAG neurons

Adult cell isolation

Synchronized young adult hermaphrodites with GFP-labeled BAG neurons (expressing a *flp-17p::gfp* transgene) were acutely dissociated as described (Kaletsky et al., 2016). Synchronized adult worms were washed with M9 buffer to remove excess bacteria. The pellet (~250 μl) was washed with 500 μl lysis buffer (200 mM DTT, 0.25% SDS, 20 mM HEPES pH 8.0, 3% sucrose) and resuspended in 750 μl lysis buffer. Worms were incubated in lysis buffer for 6.5 min at room temperature. The pellet was washed 5 times with M9 and resuspended in 20 mg/ml pronase from *Streptomyces griseus* (Roche). Worms were pipetted up and down for 12 min at room temperature; then ice-cold PBS buffer containing 2% fetal bovine serum (GIBCO) was added. Cell suspensions were passed over a 5 μm syringe filter (Millipore). The filtered cells were diluted in PBS and sorted using a Sony Biotechnology Synergy High Speed Cell Sorter. Gates for detection were set by comparison to *npr-1* cell suspensions prepared on the same day alongside the experimental samples. Positive fluorescent events were sorted directly into Eppendorf tubes containing 10 μL of 0.2% (vol/vol) Triton X-100 and 2 U μl^{-1} RNase inhibitor. Six biological replicates were prepared for each genotype, i.e., *npr-1(ad609)* and *arcp-1(db1082); npr-1(ad609)* animals. For each replicate sample, approximately 4,000 GFP positive events were collected.

RNA amplification and library preparation

RNA-seq was done using a Smart-seq2 protocol as described (Picelli et al., 2014). After neuron isolation by FACS, cDNA was prepared from each sample by reverse transcription using SuperScript II reverse transcriptase (18064-014, Invitrogen), Oligo-dT_{30} and Template-Switching Oligonucleotide (TSO) primers listed in Table S2. After the first strand reaction, the cDNA was amplified with the KAPA Hifi HotStart kit (KK2601, KAPA Biosystems) and IS PCR primers listed in Table S2. cDNA was then purified using Ampure XP beads (A 63881, Beckman Coulter), tagmented and 1 μg was used for preparing libraries with the Illumina Nextera XT DNA sample preparation kit (FC-131-1096, Illumina), as per manufacturer suggested practices. Sequencing libraries were then submitted for sequencing on the Illumina HiSeq 4000 platform.

RNA-seq data analysis

Prior to analysis the raw files were pre-processed using Bowtie2 version 0.11.0 to remove ribosomal RNA that mapped to a ribosomal RNA sequence library (Wormbase, WS255). Additionally, FASTQ files relating to the same sample but sequenced over multiple flow cell lanes were concatenated to give a single file. Custom rRNA_remover and rna_seq_lane_merger scripts were used (available on GitHub: https://github.com/lmb-seq/RNA-Seq_utilities). The files were then processed by PRAGUI - a Python3 pipeline for RNA-seq data analysis. PRAGUI automates analysis by incorporating widely used RNA-seq processing packages including: Trim Galore, FastQC, STAR, DESeq2, HTSeq, Cufflinks and MultiQC. PRAGUI can be found at: https://github.com/lmb-seq/PRAGUI. The following parameters were used with PRAGUI: DESeq2 analysis (labeled as "DESeq"), unstranded paired-end library, worm

organism with *C. elegans* genome fasta file and canonical gene set gtf file (Wormbase, WS255), STAR arguments set to "–outSAMstrandFieldDESeq intronMotif–readFilesCommand zcat -c–outSAMtype BAM SortedByCoordinate," mapq set to 20. All other PRAGUI parameters were kept default. 5.5 – 17 million reads were obtained per sample and mapped to the *C. elegans* genome. Sequences are deposited at GEO (GSE135687).

QUANTIFICATION AND STATISTICAL ANALYSIS

The number of animals and replicates used per experiment is described in detail in the ''Methods Details'' subsection for each assay and in the relevant Figure legends. Specifically, for the main behavioral assays: locomotory responses to CO_2 and O_2 were measured in >4 trials per condition with 20-30 animals each; aggregation and bordering assays were conducted with >4 trials per genotype of 50 - 60 animals each.

Statistical analyses used GraphPad Prism 7.0 and Mathworks MATLAB R2014b (8.4). Exact tests used are indicated in figure legends. In general, where more than two groups tested with a single condition were compared, a one-way ANOVA with Tukey's or Šidák's multiple comparisons test was used. Where multiple groups tested with multiple conditions were compared, a two-way ANOVA with Tukey's or Šidák's post hoc test was used. Where appropriate, a D'Agostino & Pearson or Shapiro-Wilk normality test was conducted to assess if the data fit a normal distribution. For locomotory assays where two groups were compared over one time interval, we chose time intervals where we expected the locomotory changes to have plateaued and used a Mann-Whitney *u* test for statistical comparisons as described (Laurent et al., 2015). For the intervals of interest, we determined independent per-subject means derived from individuals flagged as continuously valid for at least 10 s during the interval. We considered all individuals in the field of view as valid except those in contact with other animals and those that were off the food lawn or less than half a body-length from the border. Following these criteria, each individual was sampled at most once per interval.

DATA AND SOFTWARE AVAILABILITY

Datasets

The genome sequencing data of JU1249 and JU2825 is available on NCBI: PRJNA514933 (https://www.ncbi.nlm.nih.gov/genome/?term=PRJNA514933). The genomic sequence data of the replicate populations for QTL mapping is available on NCBI: PRJNA515248 (https://www.ncbi.nlm.nih.gov/genome/?term=PRJNA515248). Sequence data from the RNA-Seq analysis of sorted BAG neurons is deposited on GEO: GSE135687 (https://www.ncbi.nlm.nih.gov/geo/query/acc.cgi?acc=GSE135687).

Codes

Locomotory assays

Videos of locomotory assays were analyzed in Zentracker, a custom-written MATLAB software available on https://github.com/wormtracker/zentracker.

Calcium imaging

Recordings were analyzed using Neuron Analyzer, a customwritten MATLAB program available at https://github.com/neuronanalyser/neuronanalyser.

RNA-seq analysis

Codes for removing rRNA sequences from datasets and for concatenating FASTQ files relating to the same sample but sequenced over multiple flow cell lanes, are available on GitHub: https://github.com/lmb-seq/RNA-Seq_utilities. The git repository for PRAGUI can be found at: https://github.com/lmb-seq/PRAGUI.

Neuron, Volume *105*

Supplemental Information

Natural Variation in a Dendritic Scaffold Protein Remodels Experience-Dependent Plasticity by Altering Neuropeptide Expression

Isabel Beets, Gaotian Zhang, Lorenz A. Fenk, Changchun Chen, Geoffrey M. Nelson, Marie-Anne Félix, and Mario de Bono

Figure S1: Related to Figure 1

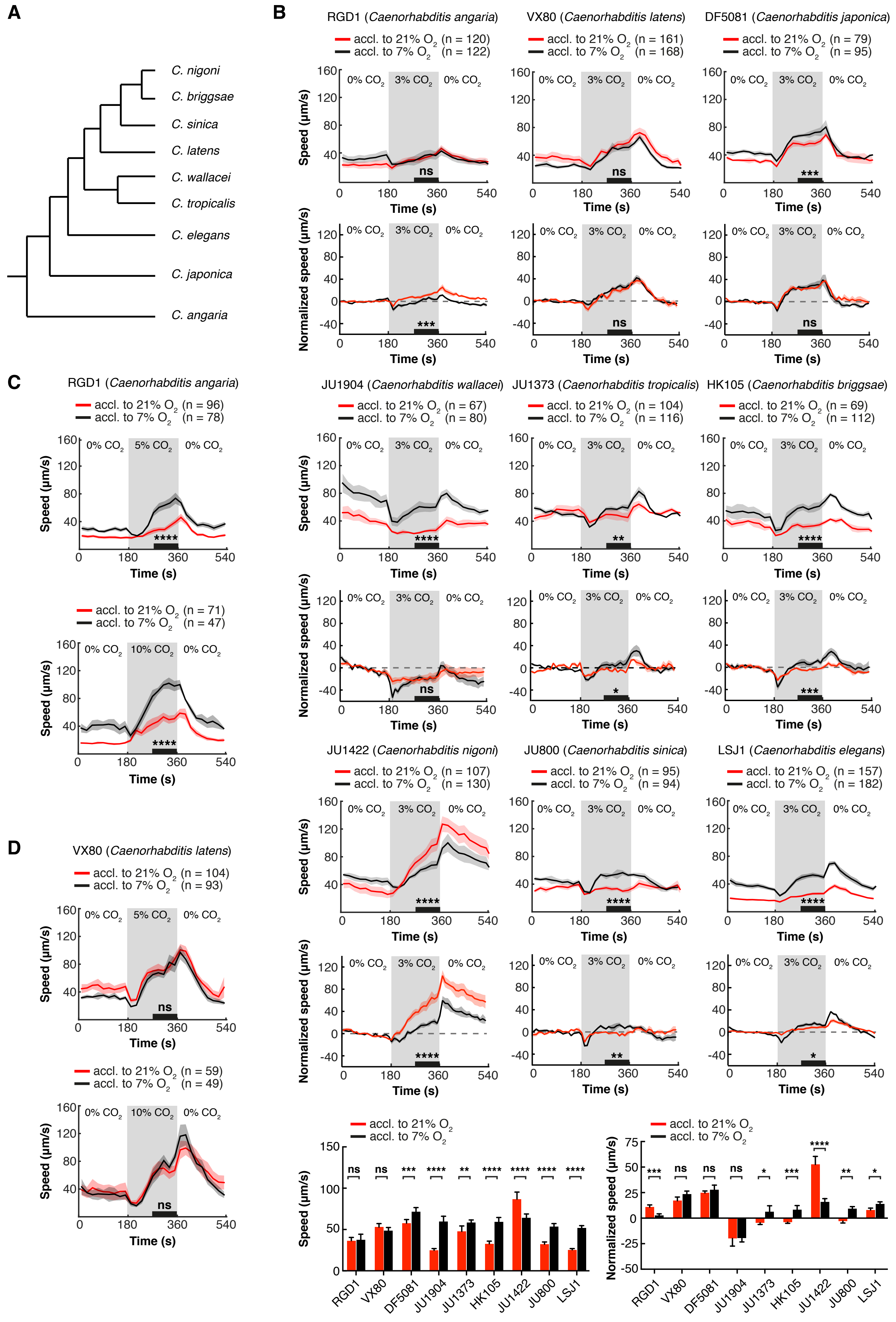

Figure S1, related to Figure 1. The effect of recent O_2 experience on CO_2 escape behavior varies across *Caenorhabditis* species. A) Cladogram of *Caenorhabditis* species studied, based on (Kiontke et al., 2011). **B)** The influence of previous O_2 experience on CO_2 escape behavior varies between *Caenorhabditis* species. In most species, including *C. elegans*, acclimation to 7% O_2 increases locomotory arousal at 3% CO_2. In *C. nigoni*, this effect is reversed and acclimation to 7% O_2 reduces CO_2-evoked locomotory arousal. In some species, such as *C. latens*, O_2 memory does not alter the effect of CO_2 on locomotory activity. For each strain, top panels show speed in response to a 0% - 3% - 0% CO_2 stimulus train. Bottom panels depict speed normalized by subtracting baseline activity before CO_2 stimulation. **C)** In *C. angaria*, the effect of O_2 memory on CO_2 escape becomes apparent at higher CO_2 concentrations. Animals acclimated to 7% O_2 are more strongly aroused by 5% and 10% CO_2 than animals acclimated to 21% O_2. **D)** O_2 memory does not influence CO_2 responses of *C. latens* at any CO_2 concentration tested.
For B-D, solid lines indicate mean speed and shaded areas show SEM. Black bars indicate the time interval used for statistical comparisons. Bar graphs plot mean speed ± SEM for these time intervals. n = number of animals tested in at least 4 trials for each condition. Mann-Whitney *u* test; * $P < 0.05$; ** $P < 0.01$; *** $P < 0.001$; **** $P < 0.0001$; ns, not significant.

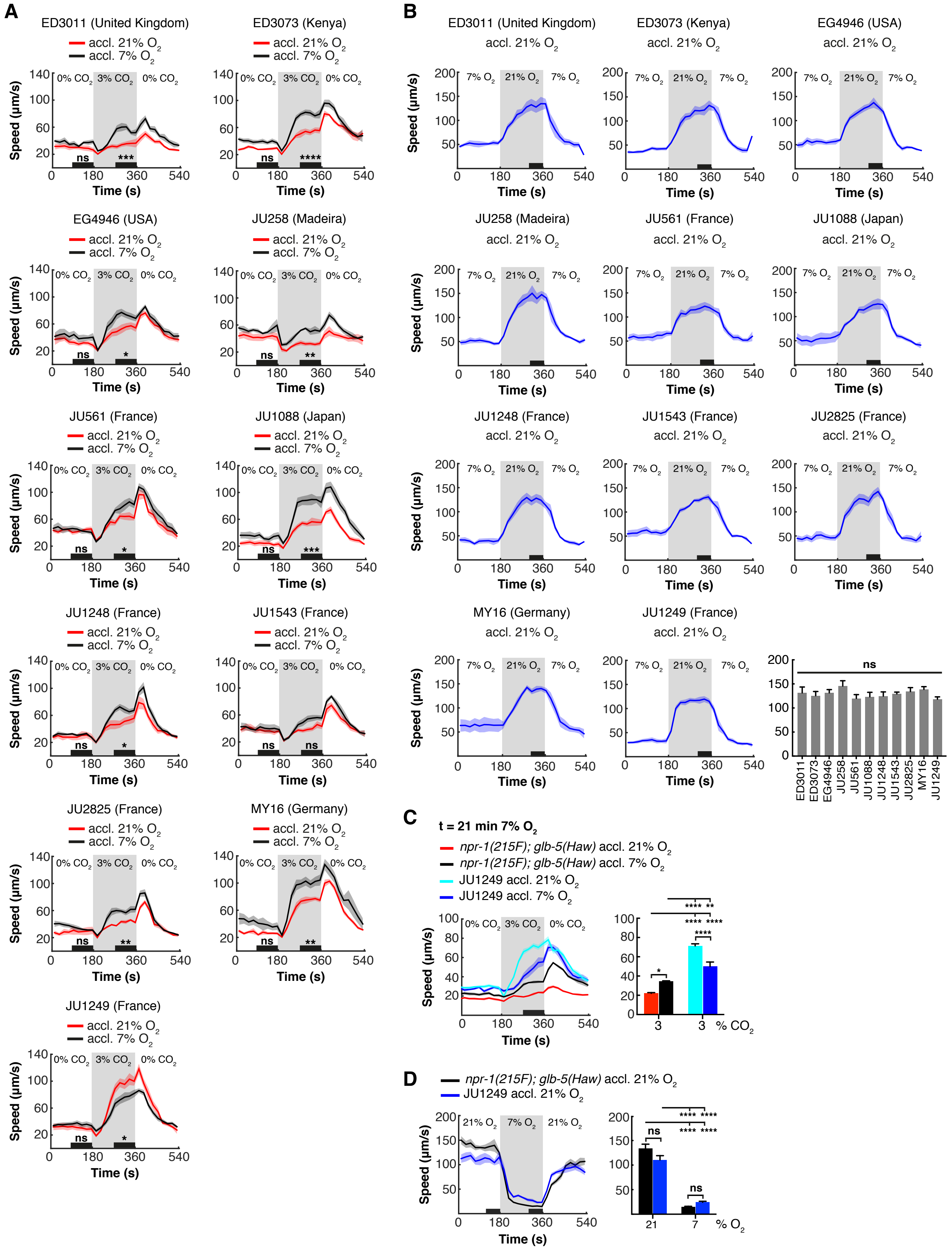
A
ED3011 (United Kingdom)
ED3073 (Kenya)
EG4946 (USA)
JU258 (Madeira)
JU561 (France)
JU1088 (Japan)
JU1248 (France)
JU1543 (France)
JU2825 (France)
MY16 (Germany)
JU1249 (France)
accl. 21% O_2
accl. 7% O_2
0% CO_2
3% CO_2
Speed (µm/s)
Time (s)
ns
*
**
***
****
B
accl. 21% O_2
7% O_2
21% O_2
ED3011
ED3073
EG4946
JU258
JU561
JU1088
JU1248
JU1543
JU2825
MY16
JU1249
C
t = 21 min 7% O_2
npr-1(215F); glb-5(Haw) accl. 21% O_2
npr-1(215F); glb-5(Haw) accl. 7% O_2
JU1249 accl. 21% O_2
JU1249 accl. 7% O_2
% CO_2
D
npr-1(215F); glb-5(Haw) accl. 21% O_2
JU1249 accl. 21% O_2
% O_2

Figure S2, related to Figure 1. *C. elegans* wild isolates show natural variation in the experience-dependent plasticity of CO_2 responses. A) How O_2 experience modulates the behavioral response to CO_2 varies across *C. elegans* wild isolates. In most isolates acclimation to 7% O_2 enhances locomotory arousal in response to CO_2. By contrast, for JU1249 acclimation to 7% O_2 suppresses locomotory arousal at 3% CO_2. Black bars indicate time intervals used for statistical comparisons here and in Figure 1C. Two-way ANOVA with Šidák test; n = 6 assays. **B)** *C. elegans* wild isolates display similar responses to 21% O_2. One-way ANOVA with post-hoc Tukey's test; n = 6 assays. **C)** JU1249 animals show altered plasticity of CO_2 responses after prolonged exposure to 7% O_2 before CO_2 stimulation. Animals acclimated to 21% or 7% O_2 were exposed to 7% O_2 in the assay chamber for 24 min before being challenged with 3% CO_2. Two-way ANOVA with post-hoc Tukey's test; n = 6 assays. **D)** JU1249 animals resemble controls in their behavioral responses to an O_2 downshift from 21% O_2 to 7% O_2. Two-way ANOVA with post-hoc Tukey's test; n = 6 assays.

For A-D, 20-30 animals were tested in 6 assays per condition. Solid lines depict the mean speed and shaded areas show the SEM. Black bars indicate time intervals used for statistical comparisons. Bar graphs plot mean speed ± SEM for these time intervals. * $P < 0.05$; ** $P < 0.01$; *** $P < 0.001$; **** $P < 0.0001$; ns, not significant.

A

4768905 ***npr-1* (chromosome X)** 4768816

```
N2      TACTTTTGCACTGAAAAGTGGGAATCTGCCAAGTCTAGAAGAGCCTACACAATGATCGTGATGCTCGCCCAATTCGTGGTCCCATTCGCT
        -Y--F--C--T--E--K--W--E--S--A--K--S--R--R--A--Y--T--M--I--V--M--L--A--Q--F--V--V--P--F--A-

JU1249  TACTTTTGCACTGAAAAGTGGGAATCTGCCAAGTCTAGAAGAGCCTACACAATGATCGTGATGCTCGCCCAATTCGTGGTCCCATTCGCT
        -Y--F--C--T--E--K--W--E--S--A--K--S--R--R--A--Y--T--M--I--V--M--L--A--Q--F--V--V--P--F--A-

JU2825  TACTTTTGCACTGAAAAGTGGGAATCTGCCAAGTCTAGAAGAGCCTACACAATGATCGTGATGCTCGCCCAATTCGTGGTCCCATTCGCT
        -Y--F--C--T--E--K--W--E--S--A--K--S--R--R--A--Y--T--M--I--V--M--L--A--Q--F--V--V--P--F--A-
```

4768815 4768759

```
N2      GTCATGGCCTTCTGCTATGCAAATATTGTTTCGGTGCTCAGCAAGCGTGCTCAGACA
        -V--M--A--F--C--Y--A--N--I--V--S--V--L--S--K--R--A--Q--T-

JU1249  GTCATGGCCTTCTGCTATGCAAATATTTTTTCGGTGCTCAGCAAGCGTGCTCAGACA
        -V--M--A--F--C--Y--A--N--I--F--S--V--L--S--K--R--A--Q--T-

JU2825  GTCATGGCCTTCTGCTATGCAAATATTTTTTCGGTGCTCAGCAAGCGTGCTCAGACA
        -V--M--A--F--C--Y--A--N--I--F--S--V--L--S--K--R--A--Q--T-
```

B

5351237 ***nath-10* (chromosome I)** 5351148

```
N2      CAGAATTCGAACGACATTACCGGAGAGCATACTTGTATTATACTGAAAGGACTCGAACACGGAGGATCTGATAGTGATGGTAAGTTAAAT
        -Q--N--S--N--D--I--T--G--E--H--T--C--I--I--L--K--G--L--E--H--G--G--S--D--S--D--E--E--P--S-

JU1249  CAGAATTCGAACGACATTACCGGAGAGCATACTTGTATTATGCTGAAAGGACTCGAACACGGAGGATCTGATAGTGATGGTAAGTTAAAT
        -Q--N--S--N--D--I--T--G--E--H--T--C--I--M--L--K--G--L--E--H--G--G--S--D--S--D--E--E--P--S-

JU2825  CAGAATTCGAACGACATTACCGGAGAGCATACTTGTATTATGCTGAAAGGACTCGAACACGGAGGATCTGATAGTGATGGTAAGTTAAAT
        -Q--N--S--N--D--I--T--G--E--H--T--C--I--M--L--K--G--L--E--H--G--G--S--D--S--D--E--E--P--S-
```

5351147 5351058

```
N2      TTGTTTTTGAATTTGAGTTTAAATTTTCGAGGAGTTTTTCTAAAGTAAATTCGTAAATTTAATTTCAAATTTCAGAAGAACCATCTGCCA
        -A--T--W--L--P--V--Y--W--R--E--F--R--R--R--I--V--N--L--L--S--F--D--F--S--S--F--P--A--Q--M-

JU1249  TTGTTTTTGAATTTGAGTTTAAATTTTCGAGGAGTTTTTCTAAAGTAAATTCGTAAATTTAATTTCAAATTTCAGAAGAACCATCTGCCA
        -A--T--W--L--P--V--Y--W--R--E--F--R--R--R--I--V--N--L--L--S--F--D--F--S--S--F--P--A--Q--M-

JU2825  TTGTTTTTGAATTTGAGTTTAAATTTTCGAGGAGTTTTTCTAAAGTAAATTCGTAAATTTAATTTCAAATTTCAGAAGAACCATCTGCCA
        -A--T--W--L--P--V--Y--W--R--E--F--R--R--R--I--V--N--L--L--S--F--D--F--S--S--F--P--A--Q--M-
```

C

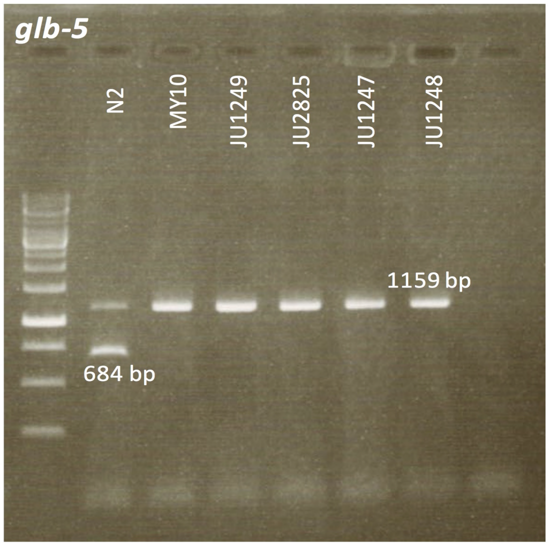

1000 bp
750 bp
500 bp

Figure S3, related to Figure 2. The wild isolate JU1249 genetically differs from the non-aggregating N2 laboratory strain. A) Partial sequence of the *npr-1* locus in the *C. elegans* N2 laboratory strain and in wild isolates JU1249 and JU2825. Unlike N2, JU1249 has the wild allele of *npr-1* (de Bono and Bargmann, 1998). **B)** JU1249 also carries the wild (non-N2) allele of the *nath-10* locus (Félix and Duveau, 2012). **C)** Gel electrophoresis of a PCR product for the *glb-5* polymorphism, as identified in (McGrath et al., 2009; Persson et al., 2009). JU1249 contains the wild *glb-5* allele, similar to other wild isolates (MY10, JU2825, JU1247 and JU1248).

Figure S4: Related to Figure 2

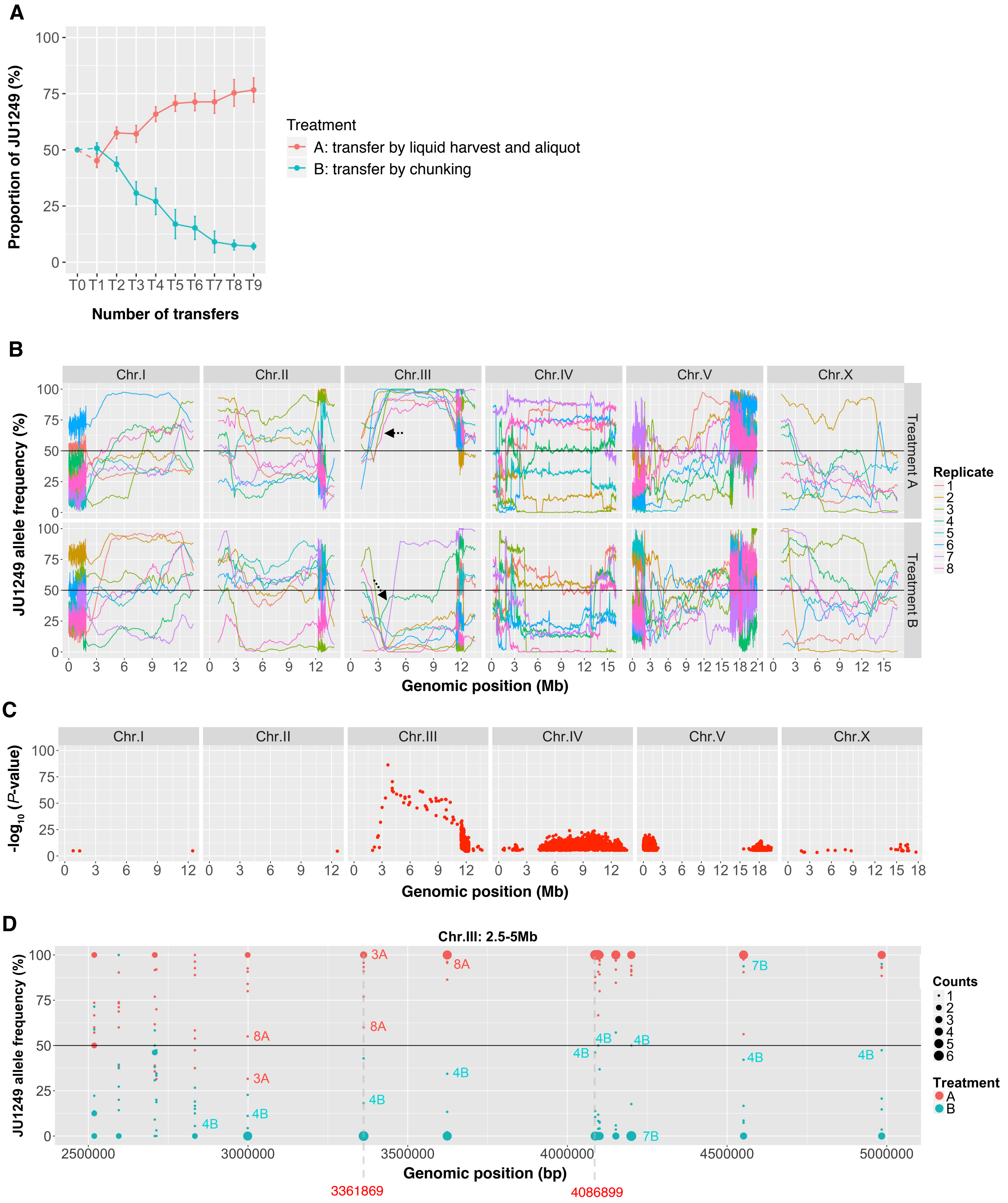

Figure S4, related to Figure 2. JU1249 allele frequency of JU1249 under different selection regimes for aggregating or solitary behavior. A) Competition assay between JU1249 and JU2825. Using SNP pyrosequencing, the proportion of JU1249 animals was measured at each transfer over the course of nine transfers. JU1249 outcompeted JU2825 in the populations transferred by Treatment A (liquid harvest and aliquot). In populations transferred by Treatment B (chunking at the lawn border where aggregating animals accumulate), JU2825 outcompeted JU1249. Plot shows the mean proportion of JU1249 animals ± SEM for five replicates of the competition assay. **B)** Average frequency of JU1249 alleles for eight replicate populations transferred by Treatment A (top panel) and Treatment B (bottom panel). For each chromosome, we used a sliding window with a width of 10 SNPs and a step size of one SNP, except for the more divergent chromosome IV (sliding window of 100-SNP). Replicates are shown in different colors. Arrows indicate recombinant selection events for replicates 8A and 4B. **C)** Cochran-Mantel-Haenszel (CMH) test for analysis of the consistency of allele frequency differences between populations among the eight population pairs. Replicate 3 was excluded for genomic positions 4396879-16406352 on Chromosome IV (see STAR Methods). Plots show significant *P*-values as $-\log_{10}$ (*P*-value) adjusted using a Bonferroni correction. **D)** JU1249 allele frequencies in the eight replicates transferred by Treatment A (red) and B (green), for the region 2,500,000-5,000,000 bp on chromosome III. Dot size represents the number of replicates at a given frequency. The candidate region of 3,361,869-4,086,899 bp is defined as the region where all replicates show a high JU1249 allele frequency in Treatment A and a low JU1249 frequency in Treatment B. The most relevant replicates for defining the interval associated with the aggregation phenotype are indicated next to the corresponding data points. The data set used in D is indicated in red in Data Table S1D and S1E.

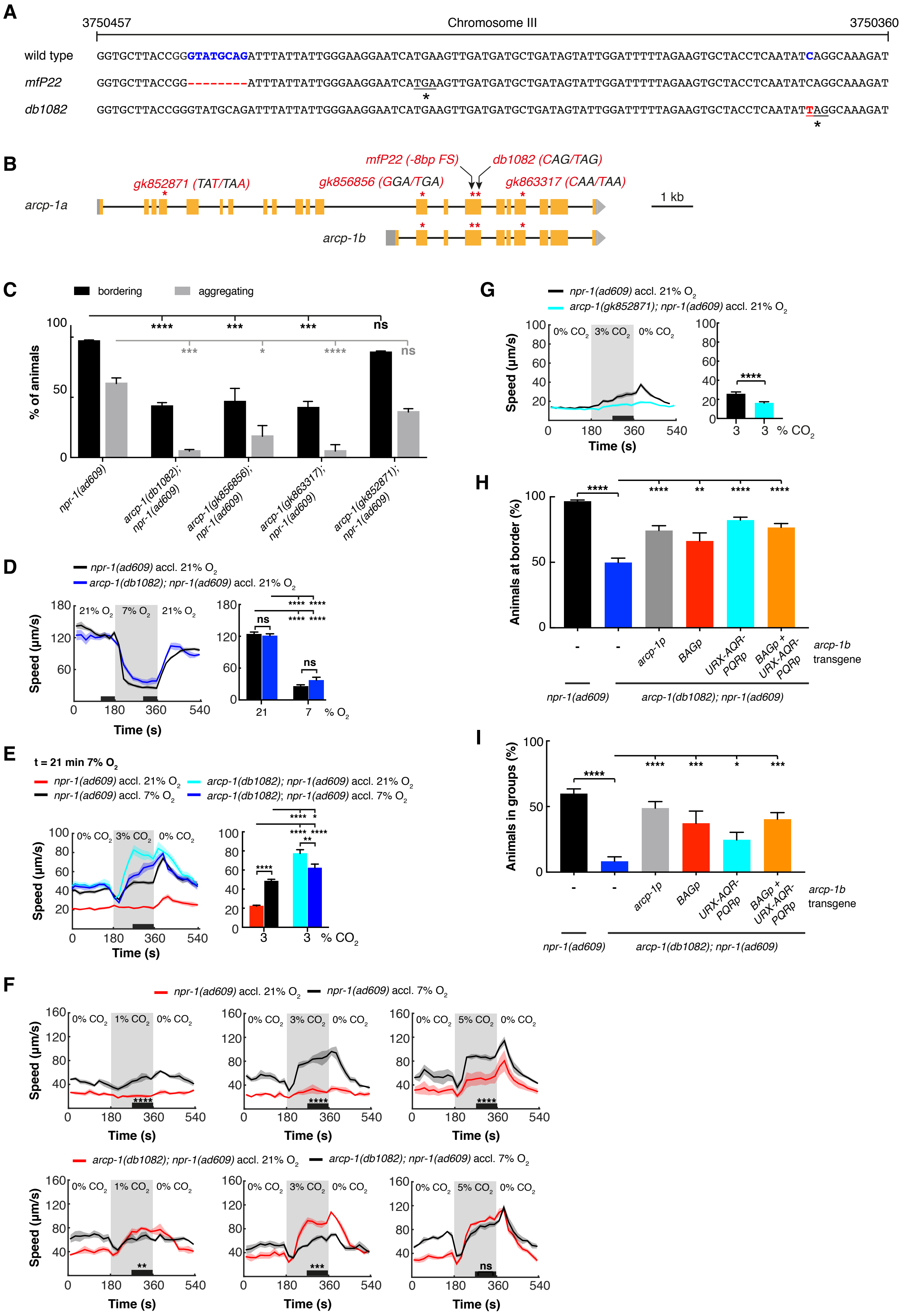
A
3750457
Chromosome III
3750360
wild type GGTGCTTACCGGGTATGCAGATTTATTATTGGGAAGGAATCATGAAGTTGATGATGCTGATAGTATTGGATTTTTAGAAGTGCTACCTCAATATCAGGCAAAGAT
mfP22 GGTGCTTACCGG--------ATTTATTATTGGGAAGGAATCATGAAGTTGATGATGCTGATAGTATTGGATTTTTAGAAGTGCTACCTCAATATCAGGCAAAGAT
db1082 GGTGCTTACCGGGTATGCAGATTTATTATTGGGAAGGAATCATGAAGTTGATGATGCTGATAGTATTGGATTTTTAGAAGTGCTACCTCAATATTAGGCAAAGAT
B
mfP22 (-8bp FS)
db1082 (CAG/TAG)
gk852871 (TAT/TAA)
gk856856 (GGA/TGA)
gk863317 (CAA/TAA)
arcp-1a
1 kb
arcp-1b
C
bordering
aggregating
% of animals
npr-1(ad609)
arcp-1(db1082); npr-1(ad609)
arcp-1(gk856856); npr-1(ad609)
arcp-1(gk863317); npr-1(ad609)
arcp-1(gk852871); npr-1(ad609)
D
npr-1(ad609) accl. 21% O2
arcp-1(db1082); npr-1(ad609) accl. 21% O2
21% O2
7% O2
21% O2
Speed (μm/s)
Time (s)
% O2
E
t = 21 min 7% O2
npr-1(ad609) accl. 21% O2
npr-1(ad609) accl. 7% O2
arcp-1(db1082); npr-1(ad609) accl. 21% O2
arcp-1(db1082); npr-1(ad609) accl. 7% O2
0% CO2
3% CO2
0% CO2
Speed (μm/s)
Time (s)
% CO2
F
npr-1(ad609) accl. 21% O2
npr-1(ad609) accl. 7% O2
1% CO2
3% CO2
5% CO2
arcp-1(db1082); npr-1(ad609) accl. 21% O2
arcp-1(db1082); npr-1(ad609) accl. 7% O2
Speed (μm/s)
Time (s)
G
npr-1(ad609) accl. 21% O2
arcp-1(gk852871); npr-1(ad609) accl. 21% O2
0% CO2
3% CO2
0% CO2
Speed (μm/s)
Time (s)
% CO2
H
Animals at border (%)
arcp-1p
BAGp
URX-AQR-PQRp
BAGp + URX-AQR-PQRp
arcp-1b transgene
npr-1(ad609)
arcp-1(db1082); npr-1(ad609)
I
Animals in groups (%)
arcp-1p
BAGp
URX-AQR-PQRp
BAGp + URX-AQR-PQRp
arcp-1b transgene
npr-1(ad609)
arcp-1(db1082); npr-1(ad609)

Figure S5, related to Figures 2 and 3. The ARCP-1B protein, but not ARCP-1A, promotes aggregation behavior and suppresses locomotory arousal by CO_2. A) Partial sequence of the *arcp-1* gene on chromosome III indicating the 8bp deletion (*mfP22*) in JU1249 and the C/T mutation of the *db1082* allele. Asterisks mark a premature stop codon. **B)** Structure of *arcp-1a* and *arcp-1b* transcripts with the positions of mutations used in this study marked by asterisks. The mutant *gk852871* allele affects only *arcp-1a*, whereas the other alleles affect both transcripts of *arcp-1*. Orange boxes represent exons; 5' and 3' UTRs are shown in gray; lines depict introns. **C)** Bordering and aggregation behaviors of the different *arcp-1* mutants. Animals with a mutation that affects only *arcp-1a* show normal bordering and aggregation, whereas mutants affected in both *arcp-1* isoforms have defects in these behaviors. Bars plot mean ± SEM. For each assay, 50-60 animals were transferred to a bacterial lawn and behaviors were scored after 2 hours. $n \geq 4$ assays for all genotypes. Kruskal-Wallis test with Dunn's correction. **D)** *arcp-1* mutants display normal behavioral responses to an O_2 downshift. Two-way ANOVA with Tukey test; n = 6 assays. **E)** *arcp-1* animals show altered plasticity of CO_2 responses when stimulated with CO_2 many minutes after transfer to 7% O_2. Animals acclimated to 21% or 7% O_2 were exposed to 7% O_2 in the assay chamber for 24 min before being challenged with 3% CO_2. Two-way ANOVA with Tukey test; n = 6 - 7 assays. **F)** Experience-dependent plasticity of CO_2 responses in *arcp-1* mutants is affected at different CO_2 concentrations. In *arcp-1* mutants, unlike *npr-1* animals, acclimation to 21% O_2 fails to suppress locomotory arousal at 1%, 3% or 5% CO_2. n = 53 - 67 animals for *npr-1*, n = 62 - 72 animals for *arcp-1; npr-1*. Mann-Whitney *u* test. **G)** Animals defective only in *arcp-1a* do not show the CO_2 phenotype observed in JU1249 and *db1082* animals. n = 97 animals for *npr-1* and n = 98 animals for *arcp-1; npr-1*. Mann-Whitney *u* test. **H-I)** The bordering and aggregation phenotypes of *arcp-1; npr-1* animals are rescued by expressing *arcp-1b* from its own promoter (*arcp-1p*), by BAG-specific expression using the *flp-17* promoter (*BAGp*), and by cell-specific expression in URX, AQR and PQR from the *gcy-32* promoter (*URX-AQR-PQRp*). Bars plot mean ± SEM. For each assay, 50-60 animals were transferred to a bacterial lawn and behaviors were scored after 6 hours. $n \geq 8$ assays. One-way ANOVA with Holm-Šidák's test. For D-G, 20-30 animals per assay were tested in at least 4 trials for each genotype. Left panel shows mean speed traces (solid lines) ± SEM (shaded areas). Black bars indicate the time intervals used for statistical comparisons. Bar graphs plot mean speed ± SEM for these intervals. * $P < 0.05$; ** $P < 0.01$; *** $P < 0.001$; **** $P < 0.0001$; ns, not significant.

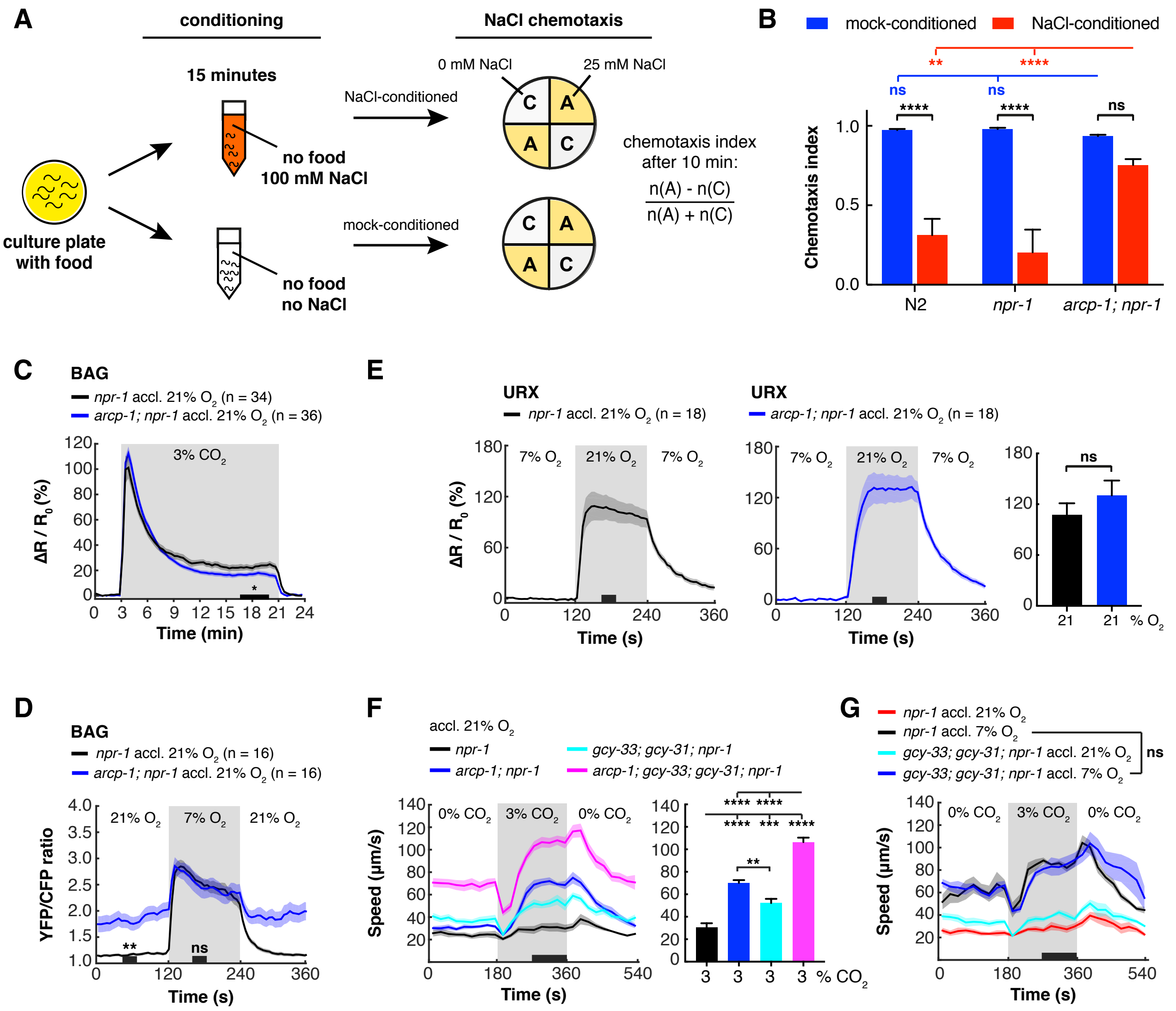
A
conditioning
15 minutes
culture plate
with food
no food
100 mM NaCl
no food
no NaCl
NaCl chemotaxis
0 mM NaCl
25 mM NaCl
NaCl-conditioned
mock-conditioned
chemotaxis index
after 10 min:
n(A) - n(C)
n(A) + n(C)
B
mock-conditioned
NaCl-conditioned
Chemotaxis index
N2
npr-1
arcp-1; npr-1
C
BAG
npr-1 accl. 21% O2 (n = 34)
arcp-1; npr-1 accl. 21% O2 (n = 36)
3% CO2
ΔR / R0 (%)
Time (min)
D
BAG
npr-1 accl. 21% O2 (n = 16)
arcp-1; npr-1 accl. 21% O2 (n = 16)
YFP/CFP ratio
21% O2
7% O2
Time (s)
E
URX
npr-1 accl. 21% O2 (n = 18)
arcp-1; npr-1 accl. 21% O2 (n = 18)
F
accl. 21% O2
npr-1
arcp-1; npr-1
gcy-33; gcy-31; npr-1
arcp-1; gcy-33; gcy-31; npr-1
Speed (µm/s)
0% CO2
% CO2
G
npr-1 accl. 21% O2
npr-1 accl. 7% O2
gcy-33; gcy-31; npr-1 accl. 21% O2
gcy-33; gcy-31; npr-1 accl. 7% O2
ns

Figure S6, related to Figures 3 and 4. Sensory responses of *arcp-1* mutants to gustatory and O_2 stimuli. A) Overview of salt-based associative learning assay. Worms were washed off their culture plate and conditioned for 15 minutes in the absence of food in a buffer with NaCl (conditioned) or without (mock-conditioned). After training, worms were placed in the center of a quadrant assay plate and their chemotaxis behavior quantified after 10 min. **B)** Chemotaxis index of mock- and NaCl-conditioned *npr-1* and *arcp-1; npr-1* animals. The N2 reference strain was used as a control for gustatory plasticity. *arcp-1; npr-1* animals show a chemotaxis index similar to that of N2 and *npr-1* animals after mock-conditioning, but are defective in the learned aversive response to NaCl after conditioning with salt in the absence of food. Two-way ANOVA with post-hoc Tukey's test. n = 8 assays with 50-100 animals per trial. **C)** Tonic Ca^{2+} response of BAG neurons in response to 3% CO_2, measured using YC3.60. Mean traces of Ca^{2+} activity are plotted as % change in R/R_0, where R is the fluorescence emission ratio at a given time point and R_0 is its initial value. n = number of animals. Mann-Whitney *u* test. **D)** O_2-evoked Ca^{2+} activity in BAG neurons of *npr-1* and *arcp-1; npr-1* animals, as YFP/CFP ratio of the cameleon sensor YC2.60. n = number of animals. Two-way ANOVA with Šidák test. **E)** Mean traces of URX Ca^{2+} activity evoked in *npr-1* and *arcp-1; npr-1* animals by 21% O_2, measured using YC2.60. n = number of animals. Mann-Whitney *u* test. **F)** *gcy-33; gcy-31; npr-1* mutants show increased locomotory arousal at 3% CO_2. *arcp-1; gcy-33; gcy-31; npr-1* quadruple mutants show an additive phenotype, indicating that these genes act in at least partly separate pathways to control CO_2 aversion. n = 10 assays with 20-30 animals per trial. One-way ANOVA with Tukey's test. **G)** *gcy-33; gcy-31; npr-1* animals show normal O_2-dependent modulation of CO_2 escape behavior. Mutant animals respond similarly to *npr-1* control animals when acclimated at 7% O_2. n = 5 assays with 20-30 animals per trial. Mann-Whitney *u* test.

For C-G, solid lines plot mean and shaded areas indicate SEM. Black bars depict time intervals used for statistical comparisons. Bar graphs plot mean ± SEM for these intervals.

* $P < 0.05$; ** $P < 0.01$; *** $P < 0.001$; **** $P < 0.0001$; ns, not significant.

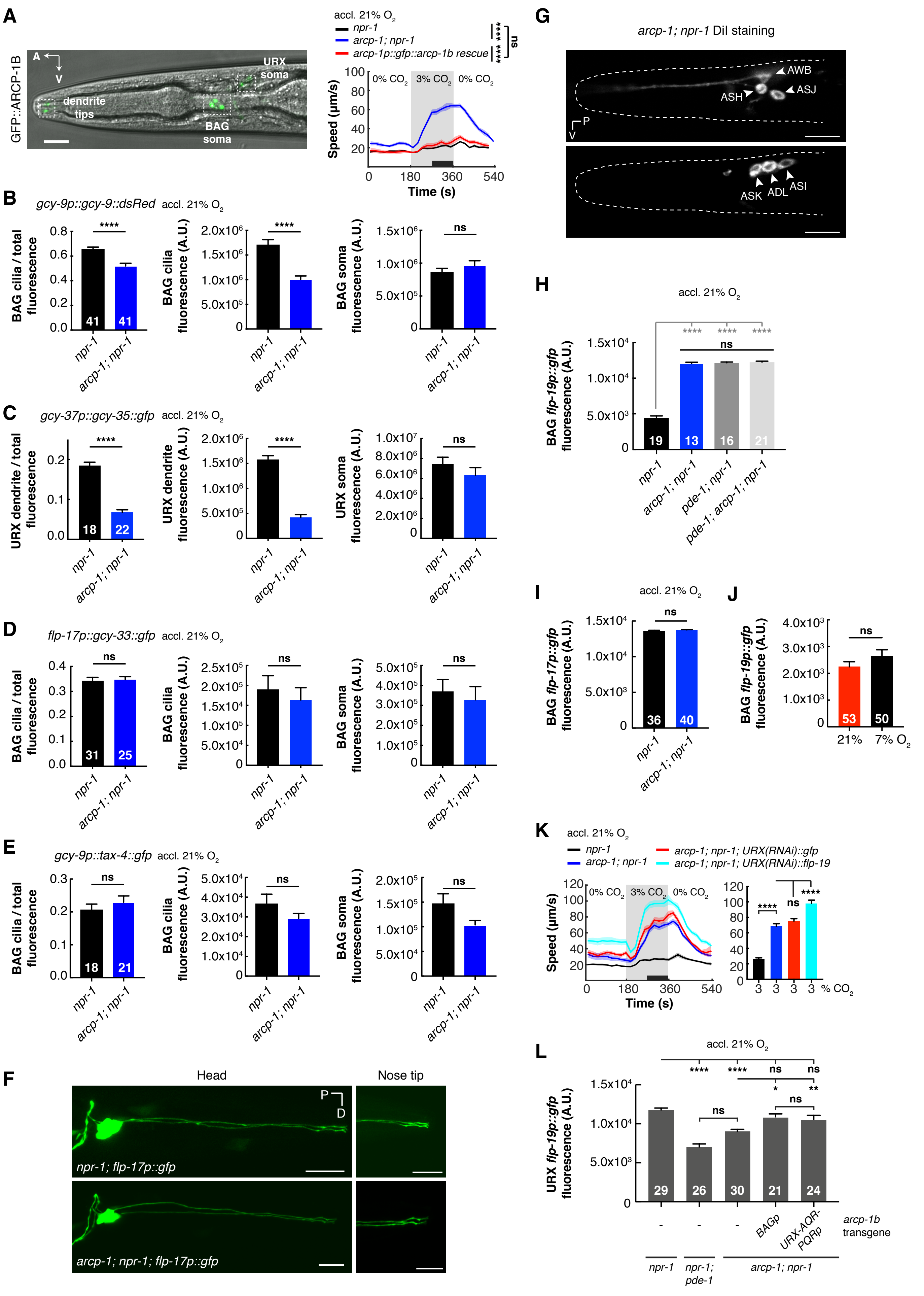
A
GFP::ARCP-1B
dendrite tips
BAG soma
URX soma
accl. 21% O2
npr-1
arcp-1; npr-1
arcp-1p::gfp::arcp-1b rescue
0% CO2 3% CO2 0% CO2
Speed (µm/s)
Time (s)
B
gcy-9p::gcy-9::dsRed accl. 21% O2
BAG cilia / total fluorescence
BAG cilia fluorescence (A.U.)
BAG soma fluorescence (A.U.)
C
gcy-37p::gcy-35::gfp accl. 21% O2
URX dendrite / total fluorescence
URX dendrite fluorescence (A.U.)
URX soma fluorescence (A.U.)
D
flp-17p::gcy-33::gfp accl. 21% O2
E
gcy-9p::tax-4::gfp accl. 21% O2
F
Head
Nose tip
npr-1; flp-17p::gfp
arcp-1; npr-1; flp-17p::gfp
G
arcp-1; npr-1 DiI staining
AWB ASJ ASH ASK ADL ASI
H
accl. 21% O2
BAG flp-19p::gfp fluorescence (A.U.)
npr-1
arcp-1; npr-1
pde-1; npr-1
pde-1; arcp-1; npr-1
I
BAG flp-17p::gfp fluorescence (A.U.)
J
BAG flp-19p::gfp fluorescence (A.U.)
21% 7% O2
K
arcp-1; npr-1; URX(RNAi)::gfp
arcp-1; npr-1; URX(RNAi)::flp-19
% CO2
L
URX flp-19p::gfp fluorescence (A.U.)
BAGp
URX-AQR-PQRp
arcp-1b transgene
npr-1; pde-1

Figure S7, related to Figures 5 and 6. Ciliary enrichment of molecular signaling components and regulation of neuropeptide expression in CO_2 and O_2 sensors. A) GFP-tagged ARCP-1B is enriched at the sensory endings of BAG neurons. The *arcp-1p::gfp::arcp-1b* transgene expresses a functional GFP-ARCP-1B protein that rescues the locomotory arousal of *arcp-1; npr-1* animals at 3% CO_2. One-way ANOVA with Tukey test. n ≥ 6 assays with 20-30 animals per trial. **B-E)** Ciliary enrichment of different tagged signaling molecules in the gas sensors, measured as the ratio of fluorescence at the sensory endings versus the total fluorescence in BAG (dendritic ending + soma). GCY-9: BAG CO_2 receptor; TAX-4: cGMP-gated ion channel subunit; GCY-33: BAG O_2 sensor; GCY-35: URX O_2 sensor. n (in bars) = number of animals. Mann-Whitney *u* test. **F)** Maximum intensity projections of confocal Z-stacks of *npr-1* and *arcp-1; npr-1* animals expressing a *flp-17p::gfp* transgene in BAG neurons. *arcp-1* mutants are not overtly defective in dendritic morphology. Scale bar = 10 µm. D = dorsal; P = posterior. **G)** DiI staining of amphid sensory neurons in *arcp-1* mutants shows normal dye filling of ciliated neurons. ARCP-1 is expressed in AWB neurons (see Figure 3C). Scale bar = 10 µm. V = ventral; P = posterior. **H)** *pde-1; arcp-1* double mutants do not show additive phenotypes, suggesting these genes act in the same pathway to regulate *flp-19* expression in BAG. n (in bars) = number of animals. One-way ANOVA with Tukey test. **I)** Expression of a fluorescent reporter for *flp-17* (*flp-17p::gfp*) is not altered by loss of *arcp-1*. n (in bars) = number of animals. Mann-Whitney *u* test. **J)** Previous O_2 experience does not influence BAG expression of a *flp-19* neuropeptide reporter (*flp-19p::gfp)* in *npr-1* animals. n (in bars) = number of animals. Mann-Whitney *u* test. **K)** CO_2-evoked locomotory arousal of *arcp-1; npr-1* mutants following knockdown of *flp-19* expression in URX neurons by expressing sense and antisense sequences from the *gcy-32* promoter. Knockdown of *flp-19* in the mutant background does not prevent locomotory arousal at 3% CO_2, and enhances baseline locomotion in the absence of CO_2; knockdown of *gfp* does not confer any phenotype. One-way ANOVA with Dunnett test. n = 11 - 12 assays with 20-30 animals per trial. **L)** Mean fluorescence ± SEM of a *flp-19p::gfp* reporter in URX, indicating that PDE-1 and ARCP-1 promote *flp-19* expression in these neurons. Cell-specific expression of *arcp-1b* in URX. AQR and PQR, using the *gcy-32* promoter, as well as expression in BAG, using the *flp-17* promoter (*BAGp*), rescues this phenotype. n (in bars) = number of animals. Kruskal-Wallis with Dunn test. For A and K, solid lines depict mean; shaded areas show SEM. Bar graphs plot mean ± SEM. * $P < 0.05$; ** $P < 0.01$; **** $P < 0.0001$; ns, not significant.

Table S1: Strain list, related to STAR Methods.

Strain	Genotype	Notes	Figures
AX1796	*glb-5(Haw) V; npr-1(g320) X*	*glb-5(Haw); npr-1(215F)*	1A-D
LSJ1	*C. elegans* Bristol strain	*glb-5(Haw); npr-1(215F)*	1G-H, S1A-B, S2C-D
RGD1	*Caenorhabditis angaria* wild isolate		S1A-C
VX80	*Caenorhabditis latens* wild isolate		S1A-B, S1D
DF5081	*Caenorhabditis japonica* wild isolate		S1A-B
JU1904	*Caenorhabditis wallacei* wild isolate		S1A-B
JU1373	*Caenorhabditis tropicalis* wild isolate		S1A-B
HK105	*Caenorhabditis briggsae* wild isolate		S1A-B
JU1422	*Caenorhabditis nigoni* wild isolate		S1A-B
JU800	*Caenorhabditis sinica* wild isolate		S1A-B
ED3011	*C. elegans* wild isolate		1B-C, S2A-B
ED3073	*C. elegans* wild isolate		1B-C, S2A-B
EG4946	*C. elegans* wild isolate		1B-C, S2A-B
JU258	*C. elegans* wild isolate		1B-C, S2A-B
JU561	*C. elegans* wild isolate		1B-C, S2A-B
JU1088	*C. elegans* wild isolate		1B-C, S2A-B
JU1248	*C. elegans* wild isolate		1B-C, S2A-B
JU1543	*C. elegans* wild isolate		1B-C, S2A-B
JU2825	*C. elegans* wild isolate		1B-C, 2A, S2A-B, S3A-C, S4
MY16	*C. elegans* wild isolate		1B-E, S2A-B
JU1249	*C. elegans* wild isolate		1B-D, 1F-H, 2B, 2F-G, 2K, 4C, S2A-D, S3A-C, S4, S5A-B
AX613	*npr-1(g320) X*	*npr-1(215F)*	2G, 2K, 4C
JU3221	*arcp-1(mfP22) III; npr-1(215F) X; mfEx94 [arcp-1p::arcp-1b::sl2::gfp; myo-2p::dsRed2]* in JU1249 background		2G, 2K
AX204	*npr-1(ad609) X*		2G, 2I-J, 3D, 4D-E, 5F, 6C-G, S5C-I, S6B, S6F-G, S7A, S7K
AX6574<br>AX7324	*arcp-1(db1082) III; npr-1(ad609) X* 4x outcrossed<br>*arcp-1(db1082) III; npr-1(ad609) X* 5x outcrossed		2F-J, 3D, 4D-E, 5F, 6C-E, S5A-F, S5H-I, S6B, S6F, S7A, S7K

AX6723	*arcp-1(db1082) III; npr-1(ad609) X; dbEx975 [arcp-1p::arcp-1b::sl2::gfp; unc-122p::rfp]*	*arcp-1p::arcp-1b*	2G, 2J
AX7094	*arcp-1(db1082) III; npr-1(ad609) X; dbEx1050 [arcp-1p::arcp-1b::sl2::mKate; lin-44p::gfp]*	*arcp-1p::arcp-1b*	2I, S5H-I
AX6720	*arcp-1(db1082) III; npr-1(ad609) X; dbEx974 [arcp-1p::arcp-1a::sl2::gfp; unc-122p::rfp]*	*arcp-1p::arcp-1a*	2J
N2	*C. elegans* Bristol strain		S3C, S6B
MY10	*C. elegans* wild isolate		S3C
JU1247	*C. elegans* wild isolate		S3C
AX6901	*arcp-1(db1082) III; npr-1(ad609) X; dbEx1002 [fosmid arcp-1p::arcp-1::sl2::gfp; unc-122p::rfp]*		3C
AX6766	*arcp-1(db1082) III; npr-1(ad609) X; dbEx984 [gcy-32p::arcp-1b::sl2::gfp; unc-122p::rfp]*	*URX-AQR-PQRp::arcp-1b*	3D, 4D
AX6805	*arcp-1(db1082) III; npr-1(ad609) X; dbEx990 [flp-17p::arcp-1b::sl2::gfp; unc-122p::rfp]*	*BAGp::arcp-1b*	3D, 4D, S5H-I
AX6931	*arcp-1(gk856856) III; npr-1(ad609) X*		S5B-C
AX6929	*arcp-1(gk863317) III; npr-1(ad609) X*		S5B-C
AX6927	*arcp-1(gk852871) III; npr-1(ad609) X*		S5B-C, S5G
AX7023	*arcp-1(db1082) III; npr-1(ad609) X; dbEx1035 [gcy-32p::arcp-1b::sl2::mKate; lin-44p::gfp]*	*URX-AQR-PQRp::arcp-1b*	S5H-I
AX7095	*arcp-1(db1082) III; npr-1(ad609) X; dbEx990 [flp-17p::arcp-1b::sl2::gfp; unc-122p::rfp]; dbEx1035 [gcy-32p::arcp-1b::sl2::mKate; lin-44p::gfp]*	*BAGp + URX-AQR-PQRp*	S5H-I
AX7179	*gcy-9(n4470) npr-1(ad609) X*		4E
AX7238	*arcp-1(db1082) III; gcy-9(n4470) npr-1(ad609) X*		4E
AX7116	*arcp-1(db1082) III; npr-1(ad609) X; dbIs20 [arcp-1p::gfp::arcp-1b; unc-122p::rfp]*	GFP-ARCP-1B	5B-C, S7A
AX6969	*malt-1(db1194) II; npr-1(ad609) X; dbIs16 [rab-3p::malt-1::gfp; unc-122p::rfp]*	(1) GFP control	5B
AX7082	*eif-3.L(db1015) II; npr-1(ad609) X; dbIs19 [rab-3p::eif-3.L::gfp; unc-122p::rfp]*	(2) GFP control	5B
AX7419	*npr-1(ad609) X dbEx1075 [flp-17p::pde-1b::gfp; unc-122p::rfp]*	PDE-1B-GFP	5D-E
AX7422	*arcp-1(db1082) III; npr-1(ad609) X; dbEx1075 [flp-17p::pde-1b::gfp; unc-122p::rfp]*		5E
AX2272	*pde-1(ok2924) I; npr-1(ad609) X*		5F
AX7453	*pde-1(ok2924) I; arcp-1(db1082) III; npr-1(ad609) X*		5F
AX6881	*npr-1(ad609) X dbEx [flp-17p::YC3.60]*		4A-B, S6C
AX6893	*arcp-1(db1082) III; npr-1(ad609) X; dbEx [flp-17p::YC3.60]*		4A-B, S6C
AX7842	*arcp-1(db1082) III; npr-1(ad609) X; dbEx [flp-17p::YC3.60]; dbEx1035 [gcy-32p::arcp-1b::sl2::mKate; lin-44::gfp]*	*URX-AQR-PQRp::arcp-1b*	4B
AX7845	*arcp-1(db1082) III; npr-1(ad609) X; dbEx [flp-17p::YC3.60] dbEx1172 [gcy-33p::arcp-1b::sl2::mKate; unc-122p::gfp]*	*BAGp::arcp-1b*	4B
AX3516	*npr-1(ad609) X; dbEx614 [gcy-37p::YC2.60; unc-122p::rfp]*		S6E
AX6877	*arcp-1(db1082) III; npr-1(ad609) X; dbEx614 [gcy-37p::YC2.60; unc-122p::rfp]*		S6E
AX3432	*npr-1(ad609) X; dbEx623 [flp-17p::YC2.60; F15E11.1::mCherry]*		S6D
AX7182	*arcp-1(db1082) III; npr-1(ad609) X; dbEx623 [flp-17p::YC2.60; F15E11.1::mCherry]*		S6D
AX7656	*gcy-33(ok232) V; gcy-31(ok296) npr-1(ad609) X*		S6F-G
AX7657	*arcp-1(db1082) III; gcy-33(ok232) V; gcy-31(ok296) npr-1(ad609) X*		S6F
AX7362	*npr-1(ad609) X; wzIs132 [gcy-9p::gcy-9::dsRed]*		S7B

AX7361	*arcp-1(db1082) III; npr-1(ad609) X; wzIs132 [gcy-9p::gcy-9::dsRed]*		S7B
AX7366	*npr-1(ad609) X; wzEx156 [gcy-9p::tax-4::gfp]*		S7E
AX7365	*arcp-1(db1082) III; npr-1(ad609) X; wzEx156 [gcy-9p::tax-4::gfp]*		S7E
AX2997	*gcy-33(ok232) V; npr-1(ad609) X; dbEx [flp-17p::gcy-33::gfp; unc-122p::rfp]*		S7D
AX7315	*arcp-1(db1082) III; gcy-33(ok232) V; npr-1(ad609) X; dbEx [flp-17p::gcy-33::gfp; unc-122p::rfp]*		S7D
AX6516	*npr-1(ad609) X; dbEx1053 [gcy-37p::gcy-35::HA::gfp::sl2::mCherry]*		S7C
AX7278	*arcp-1(db1082) III; npr-1(ad609) X; dbEx1053 [gcy-37p::gcy-35::HA::gfp::sl2::mCherry]*		S7C
AX7019	*arcp-1(db1082) III; npr-1(ad609) X; dbEx1033 [flp-17p::gfp; unc-122p::rfp]*		S7F
AX7021	*npr-1(ad609) X; dbEx1033 [flp-17p::gfp; unc-122p::rfp]*		S7F
AX7268	*npr-1(ad609) X; ynIs34 [flp-19p::gfp]*		6A, S7H, S7J, S7L
AX7271	*arcp-1(db1082) III; npr-1(ad609) X; ynIs34 [flp-19p::gfp]*		6A, S7H, S7L
AX7279	*pde-1(ok2924) I; npr-1(ad609) X; ynIs34 [flp-19p::gfp]*		6A, S7H, S7L
AX7272	*arcp-1(db1082) III; npr-1(ad609) X; ynIs34 [flp-19p::gfp]; dbEx1063 [flp-17p::arcp-1b::sl2::mKate; unc-122p::gfp]*	*BAGp::arcp-1b*	6A, S7L
AX7273	*arcp-1(db1082) III; npr-1(ad609) X; ynIs34 [flp-19p::gfp] dbEx1035 [gcy-32p::arcp-1b::sl2::mKate; lin-44::gfp]*	*URX-AQR-PQRp::arcp-1b*	6A, S7L
AX7550	*pde-1(ok2924) I; arcp-1(db1082) III; npr-1(ad609) X; ynIs34 [flp-19p::gfp]*		S7H
AX7722	*ynIs34 [flp-19p::gfp]* backcrossed 10x in JU2825 background		6B
AX7724	*ynIs34 [flp-19p::gfp]* backcrossed 10x in JU1249 background		6B
AX7726	*dbEx1063 [flp-17p::arcp-1b::sl2::mKate; unc-122p::gfp]; ynIs34 [flp-19p::gfp]* backcrossed 10x in JU1249 background		6B
AX7210	*npr-1(ad609) X; ynIs64 [flp-17p::gfp]*		S7I
AX7208	*arcp-1(db1082) III; npr-1(ad609) X; ynIs64 [flp-17p::gfp]*		S7I
AX7321	*flp-19(ok2460) npr-1(ad609) X*		6C
AX7322	*arcp-1(db1082) III; flp-19(ok2460) npr-1(ad609) X*		6C
AX7754	*arcp-1(db1082) III; npr-1(ad609) X; dbEx1171 [gcy-33p::gfp (sas); unc-122p::rfp]*	*BAG(RNAi)::gfp*	6D-E
AX7760	*arcp-1(db1082) III; npr-1(ad609) X; dbEx1173 [gcy-33p::flp-19 (sas); unc-122p::rfp]*	*BAG(RNAi)::flp-19*	6D-E
AX7788	*arcp-1(db1082) III; npr-1(ad609) X; dbEx1178 [gcy-32p::gfp (sas); unc-122p::rfp]*	*URX(RNAi)::gfp*	S7K
AX7678	*arcp-1(db1082) III; npr-1(ad609) X; dbEx1153 [gcy-32p::flp-19 (sas); unc-122p::gfp]*	*URX(RNAi)::flp-19*	S7K
AX7793	*npr-1(ad609) X; dbEx1173 [gcy-33p::flp-19 (sas); unc-122p::rfp]*	*BAG(RNAi)::flp-19*	6F
AX7437	*npr-1(ad609) X; dbEx1077 [flp-17p::flp-19::sl2::mKate; unc-122p::gfp]*	*BAGp::flp-19*	6G

Table S2, related to STAR Methods. List of primers used in this study.

PRIMER	SOURCE	IDENTIFIER
npr-1 genotyping Fw<br>CTCCACCATTTTGCGTATCTTTGT	(Duveau and Félix, 2012)	N/A
npr-1 genotyping Rev<br>AGGAAGAGAGAATATGCGGCTAC	(Duveau and Félix, 2012)	N/A
glb-5 genotyping Fw<br>GGTGACTCGTTGGATGATGA	(McGrath et al., 2009)	N/A
glb-5 genotyping Rev1<br>GTGATGAGCTCCCATTTCGT	(McGrath et al., 2009)	N/A
glb-5 genotyping Rev2<br>TTCTGCTCCAGAAGACACGA	(McGrath et al., 2009)	N/A
nath-10 genotyping Fw<br>GCCGGGAACGAGGAAAAGTCAAATG	(Duveau and Félix, 2012)	N/A
nath-10 genotyping Rev<br>TTCGGACTCACTGTTCCACTTTTA	(Duveau and Félix, 2012)	N/A
arcp-1 genotyping Fw<br>AAACGCAGTTGCAGATTGAA	This paper	N/A
arcp-1 genotyping Rev1<br>TCGATCGATGAAAGACAACG	This paper	N/A
arcp-1 genotyping Rev2<br>GAATTGGTGCTTACCGGATT	This paper	N/A
arcp-1a cDNA cloning Fw<br>GGGGACAAGTTTGTACAAAAAAGCAGGCTTTTCAGAAAA**ATGCGACCACCTCCACTTGTTC**	This paper	N/A
arcp-1a cDNA cloning Rev<br>GGGGACCACTTTGTACAAGAAAGCTGGGT**TCAATGACTTCCATTGTGACGTGG**	This paper	N/A
arcp-1b cDNA cloning Fw<br>GGGGACAAGTTTGTACAAAAAAGCAGGCTTTTCAGAAAA**ATGCATGAGCGTCTACCATTTGATC**	This paper	N/A
arcp-1b cDNA cloning Rev<br>GGGGACCACTTTGTACAAGAAAGCTGGGT**TCAATGACTTCCATTGTGACGTGG**	This paper	N/A
pde-1b cDNA cloning Fw<br>GGGGACAAGTTTGTACAAAAAAGCAGGCTTTTCAGAAAA**ATGATGATCGTCGGGGATACC**	This paper	N/A
pde-1b cDNA cloning Rev<br>GGGGACCACTTTGTACAAGAAAGCTGGGT**AATTCGTTGTGACGCCATTTTC**	This paper	N/A
flp-19 cDNA cloning Fw<br>GGGGACAAGTTTGTACAAAAAAGCAGGCTTTTCAGAAAA**ATGTCCTTCCAGCTAACGCTGT**	This paper	N/A
flp-19 cDNA cloning Rev<br>GGGGACCACTTTGTACAAGAAAGCTGGGT**TTATCCGAACCTGACTGAGCTGG**	This paper	N/A

III_663310 pyrosequencing Fw<br>GTGACGTACTAGCAACGAGTCGATTTTGGGGATGGA	This paper	N/A
III_663310 pyrosequencing Rev<br>CACTAGGCAGGTAGGCATTTTT	This paper	N/A
III_663310 pyrosequencing Sequencing<br>CCAAACTTTTATAGAGATCA	This paper	N/A
Universal biotinylated primer for pyrosequencing<br>[Btn]TAGCAGGATACGACTATC	(Richaud et al., 2018)	N/A
Oligo-dT_{30} VN for RNA-Seq<br>AAGCAGTGGTATCAACGCAGAGTACT_{30}VN where "N" is any base and "V" is either A, C or G	(Picelli et al., 2014)	N/A
TSO for RNA-Seq<br>AAGCAGTGGTATCAACGCAGAGTACATrGrG+G with "rG' riboguanosine and "+G" LNA-modified guanosine	(Picelli et al., 2014)	N/A
IS PCR primer for RNA-Seq<br>AAGCAGTGGTATCAACGCAGAGT	(Picelli et al., 2014)	N/A
